# Co-cultivation With 5-Azacytidine Induced New Metabolites From the Zoanthid-Derived Fungus *Cochliobolus lunatus*

**DOI:** 10.3389/fchem.2019.00763

**Published:** 2019-11-08

**Authors:** Jing-Shuai Wu, Xiao-Hui Shi, Ya-Hui Zhang, Jia-Yin Yu, Xiu-Mei Fu, Xin Li, Kai-Xian Chen, Yue-Wei Guo, Chang-Lun Shao, Chang-Yun Wang

**Affiliations:** ^1^Key Laboratory of Marine Drugs, The Ministry of Education of China, School of Medicine and Pharmacy, Ocean University of China, Qingdao, China; ^2^Laboratory for Marine Drugs and Bioproducts, Qingdao National Laboratory for Marine Science and Technology, Qingdao, China; ^3^Open Studio for Druggability Research of Marine Natural Products, Pilot National Laboratory for Marine Science and Technology (Qingdao), Qingdao, China; ^4^Institute of Evolution and Marine Biodiversity, Ocean University of China, Qingdao, China

**Keywords:** chemical epigenetic manipulation, DNA methyltransferase inhibitor, *Cochliobolus lunatus*, α-pyrones, DP4+ probability calculation, the fragment molecules

## Abstract

The zoanthid-derived fungus *Cochliobolus lunatus* (TA26-46) has been proven to be a source of bioactive 14-membered resorcylic acid lactones (RALs). In the present study, chemical epigenetic manipulation was applied to this fungal strain with a DNA methyltransferase inhibitor resulting in the significant changes of the secondary metabolites. Cultivation of *C. lunatus* (TA26-46) with 10 μM 5-azacytidine in Czapek-Dox liquid medium led to the isolation of new types of metabolites, including two α-pyrones, cochliobopyrones A (**1**) and B (**2**), along with three isocoumarins (**3**–**5**) and one chromone (**6**). The planar structures of the new compounds (**1**–**2**) were elucidated by comprehensive analyses of NMR and HRESIMS data. Their challenging relative configurations were established by a combination of acetonide reaction, coupling constants and NOESY correlations analysis, and DP4+ probability calculation. Their absolute configurations were determined by comparing with the ECD calculation data of the fragment molecules, 6-(1,2-dihydroxypropyl)-4-methoxy-2*H*-pyran-2-ones. It is the first time to obtain α*-pyrone* compounds with the epoxy ring or bromine atom on the seven-numbered side chain. It could be concluded that chemical epigenetic agents could induce *C. lunatus* to produce new types of secondary metabolites differing from its original products (RALs).

## Introduction

Marine fungi have been proved to be able to produce a wealth of structurally novel and biologically potent secondary metabolites (Jin et al., 2016; Blunt et al., 2018). Nevertheless, more and more genome sequencing results have revealed that most biosynthetic gene clusters of marine fungi are silent or expressed at low levels under standard laboratory conditions, indicating that the capacity of marine fungi to provide compounds is far more than we anticipated (Rutledge and Challis, 2015). It is a useful alternative to activate the silent gene clusters of marine fungi to increase chemical diversity and novelty of secondary metabolites, especially by cultivation-dependent strategies, such as OSMAC, co-culture and chemical epigenetics (Rutledge and Challis, 2015). In recent years, chemical epigenetic manipulation has been widely used as an effective and handy approach to harvest cryptic secondary metabolites from microbes (Cichewicz, 2010; Cherblanc et al., 2013). Consequently, this approach has proven to be able to induce new metabolic pathway to produce new type of compounds. However, the epigenetic perturbation with chemical modifying agents has only been applied to limited marine-derived fungi to discover new secondary metabolites. For example, a new cyclodepsipeptide of hybrid biosynthetic product EGM-556 was obtained from a Floridian marine sediment-derived fungus *Microascus* sp. treated with a histone deacetylase inhibitor, SAHA (Vervoort et al., 2010). A series of new bisabolene-type sesquiterpenoids were harvested from a sea sediment-derived fungus *Aspergillus sydowii* stimulated by a DNA methyltransferase inhibitor, 5-azacytidine (Chung et al., 2013). Therefore, chemical epigenetic manipulation exhibited tremendous potential for excavating cryptic secondary metabolites from marine-derived fungi.

In our previous studies, a series of 14-membered resorcylic acid lactones (RALs) were isolated from the zoanthid-derived fungus *Cochliobolus lunatus* (TA26-46) cultured in rice medium (Shao et al., 2011; Liu et al., 2014; Xu et al., 2019). To explore the biosynthetic potential of this fungus, chemical epigenetic manipulation was conducted by using DNA methyltransferase inhibitors and histone deacetylase inhibitors to treat this fungus cultured on starch solid medium, resulting in the production of new metabolites, including diethylene glycol phthalate esters (Chen et al., 2016) and brominated RALs (Zhang et al., 2014). In the present study, in order to further mine the metabolic potential of *C. lunatus* (TA26-46), the fungal strain was treated with different epigenetic modifiers in Czapek-Dox liquid medium. By using DNA methyltransferase inhibitor, 5-azacytidine, besides of the original 14-membered RALs, six induced metabolites were obtained from the culture, including two new α-pyrones, three known isocoumarins, and one known chromone. Herein, we reported the chemical epigenetic manipulation of the strain, and the isolation, structure elucidation, and bioactivity evaluation of the induced metabolites.

## Materials and Methods

### General Experimental Procedure

Optical rotations were measured on a JASCO P-1020 digital polarimeter. UV spectra were recorded on a HITACHI UH 5300 UV spectrophotometer. ECD data were acquired on a J-815-150S Circular Dichroism spectrometer. IR spectra were recorded on a Nicolet-Nexus-470 spectrometer using KBr pellets. NMR spectra were acquired by a JEOL JEM-ECP NMR spectrometer (500 MHz for ^1^H and 125 MHz for ^13^C), using TMS as an internal standard. HRESIMS were measured on Agilent 1290 Infinity II UHPLC/6530 Q-TOF MS for compounds **1** and **2**, and Thermo MAT95XP high resolution mass spectrometer for compound **3**. Samples were analyzed and prepared on a Hitachi L-2000 HPLC system coupled with a Hitachi L-2455 photodiode array detector and using a semi-prepared C_18_ column (Kromasil 250 × 10 mm, 5 μm). Silica gel (Qing Dao Hai Yang Chemical Group Co.; 300–400 mesh) and Sephadex LH-20 (Amersham Biosciences) were used for column chromatography (CC). Precoated silica gel plates (Yan Tai Zi Fu Chemical Group Co.; G60, F-254) were used for thin-layer chromatography.

### Fungal Material

The fungus *C. lunatus* (TA26-46) was isolated from a piece of fresh inner part tissue of the zoanthid, collected from the Weizhou coral reef in the South China Sea in April 2010. The fungal strain was deposited at the Key Laboratory of Marine Drugs, the Ministry of Education of China, School of Medicine and Pharmacy, Ocean University of China, Qingdao, People's Republic of China. The GenBank (NCBI) access number was JF819163.

### Cultivation, Extraction, and Isolation

In an analysis-scale fermentation, *C. lunatus* (TA26-46) was cultivated in the Czapek-Dox medium (sucrose 30 g/L, sodium nitrate 3 g/L, K_2_HPO_4_ 1 g/L, FeSO_4_ · 7H_2_O 0.01 g/L, MgSO_4_ · 7H_2_O 0.5 g/L, KCl 0.5 g/L, artificial sea salt, 30 g/L) with addition of different chemical epigenetic agents (5-azacytidine, 2′-deoxy-5-azacytidine, SAHA, SBHA, sodium butyrate, and nicotinamide) with different concentrations. The ethyl acetate (EtOAc) extracts of the cultures were analyzed by HPLC. The HPLC condition was as follow: C18 column (Kromasil 250 × 10 mm, 10 μm); a gradient of 5–100% MeOH in H_2_O for 60 min at the flow rate of 2 mL/min, and recorded at 210 nm.

In a large-scale fermentation, sixty 1 L Erlenmeyer flasks of the fungal strain were cultivated in the Czapek-Dox medium with addition of 10 μM 5-azacytidine for 30 days at room temperature. The fermentation broth and mycelia were extracted repeatedly with equal amount of EtOAc for three times, which was evaporated in vacuo to afford an EtOAc extract (13 g). The crude extract was isolated on silica gel column chromatography (CC) using a step gradient elution with petroleum ether/EtOAc (10:1 to 1:4, v/v) to provide five fractions (Fr.1–Fr.5). Fr.2 was subjected to Sephadex LH-20 CC eluted with CH_2_Cl_2_/MeOH (1:1) and semi-preparative HPLC with MeOH/H_2_O (60: 40) to give **3** (2.5 mg, RT = 25.5 min) and RAL2 (3.5 mg). Fr.3 was separated by column chromatography on Sephadex LH-20 CC eluted with CH_2_Cl_2_/MeOH (1:1) to obtain **6** (4.2 mg). Fr.4 were separated on ODS CC eluted with MeOH/H_2_O (40% → 70%) to yield five fractions (Fr.4-1–Fr.4-5). Fr.4-2 was subjected to Sephadex LH-20 eluted with CH_2_Cl_2_/MeOH (1:1) and further semi-preparative HPLC with MeOH/H_2_O (30:70) to afford RAL1 (9.3mg). Fr.4-3 was separated by semi-preparative HPLC with MeOH/H_2_O (30:70) to give **1** (3.7 mg, RT = 37.1 min) and **2** (2.2 mg, RT = 38.7 min). Fr.4-4 was purified by semi-preparative HPLC with MeOH/H_2_O (65:45) to obtain **4** (7.6 mg, RT = 20.3 min) and **5** (5.6 mg, RT = 26.9 min).

*Cochliobopyrone A (****1****)*: C_13_H_19_O_6_Br; yellowish oil; ${\left[\alpha \right]}_{\text{D}}^{20}$ −12.5 (*c* 0.03, MeOH); UV (MeOH) λ_max_ (log ε) 271 (1.06) nm; IR (KBr) ν_max_ 3748, 3673, 3650, 2360, 2338, 1734, 1699, 1650, 1540, 1457 cm^−1^; ^1^H NMR (500 MHz, DMSO-*d*_6_) and ^13^C NMR (125 MHz, DMSO-*d*_6_), see Table 1; HRESIMS *m/z*: 373.0262 [M + Na]^+^ (calcd for C_13_H_19_O_6_BrNa, 373.0257).

**Table 1:** ^1^H NMR and ^13^C NMR Data for **1** and **2**^a^.

**Position**	**1**		**2**	
	**δ_**C**_, type**	**δ_**H**_ (*J* in Hz)**	**δ_**C**_, type**	**δ_**H**_ (*J* in Hz)**
2	163.7, C		163.4, C	
3	87.7, CH	5.54, d (2.0)	88.6, CH	5.58, d (2.0)
4	171.5, C		171.2, C	
5	99.4, CH	6.13, d (2.0)	99.2, CH	6.17, d (2.0)
6	167.4, C		164.8, C	
7	70.5, CH	4.75, d (6.8)	70.4, CH	4.12, t (5.8)
8	71.4, CH	4.04, m	57.0, CH	3.07, dd (5.8, 2.0)
9	62.9, CH	4.12, m	58.9, CH	2.92, dd (4.0, 2.0)
10	67.0, CH	3.86, m	68.0, CH	3.41, m
11	38.8, CH_2_	1.57, m	36.5, CH_2_	1.37, m
		1.36, m		1.31, m
12	19.0, CH_2_	1.36, m	18.3, CH_2_	1.37, m
		1.27, m		1.31, m
13	14.5, CH_3_	0.89, t (7.0)	14.5, CH_3_	0.83, t (6.7)
14	56.8, CH_3_	3.81, s	56.8, CH_3_	3.83, s
7-OH		5.84, d (6.8)		6.04, d (5.8)
8-OH		5.22, d (8.4)		
10-OH		4.70, d (6.1)		4.71, d (5.1)

a *500 MHz for ^1^H NMR and 125 MHz for ^13^C NMR in DMSO-d_6_*.

*Cochliobopyrone B (****2****)*: C_13_H_18_O_6_; white powder; ${\left[\alpha \right]}_{\text{D}}^{20}$ −19.5 (*c* 0.01, MeOH); UV (MeOH) λ_max_ (log ε) 271 (1.95) nm; IR (KBr) ν_max_ 3748, 2360, 2338, 1715, 1700, 1650, 1540, 1509, 1457 cm^−1^; ^1^H NMR (500 MHz, DMSO-*d*_6_) and ^13^C NMR (125 MHz, DMSO-*d*_6_), see Table 1; HRESIMS *m/z*: 293.0998 [M + Na]^+^ (calcd for C_13_H_18_O_6_Na, 293. 0996).

*6-Hydroxy-8-methoxy-3-methylisocoumarin (****3****)*: C_11_H_10_O_4_; white powder; UV (MeOH) λ_max_ (log ε) 238 (1.91), 251 (0.75), 319 (0.43) nm; IR (KBr) ν_max_ 3749, 3674, 2360, 1701, 1368, 1178 cm^−1^; ^1^H NMR (500 MHz, DMSO-*d*_6_): 6.39 (1H, d, *J* = 1.5 Hz, H-7), 6.29 (1H, d, *J* = 1.5 Hz, H-5), 6.24 (1H, s, H-4), 3.78 (3H, s, H-10), 2.10 (3H, s, H-9). ^13^C NMR (125 MHz, DMSO-*d*_6_): 165.1 (C, C-1), 163.6 (C, C-8), 158.3 (C, C-6), 155.0 (C, C-3), 142.2 (C, C-4a), 103.5 (CH, C-4), 102.9 (CH, C-5), 100.4 (C, C-8a), 99.2 (CH, C-7), 56.8 (CH_3_, C-10), 19.4 (CH_3_, C-9); HRESIMS *m/z*: 207.0651 [M + H]^+^ (calcd for C_11_H_11_O_4_, 207. 0652) and *m/z* 229.0469 [M + Na]^+^ (calcd for C_11_H_10_O_4_Na, 229. 0471).

### Preparation of the Acetonide Derivatives 1a

The reaction mixture, including compound **1** (1.5 mg), 2,2-dimethoxypropane (0.7 mL), and *p*-TsOH (0.1 mg) was stirred 3 h at room temperature. The reaction was stopped by adding 1 mL saturated aqueous NaHCO_3_, and then extracted with equal amount of EtOAc for 3 times. The extract was concentrated under vacuum condition. The crude mixture was subjected to semi-preparative HPLC with MeOH/H_2_O (40:60) to obtain **1a** (1.3 mg).

*Compound* ***1a***: C_16_H_23_O_6_Br; white powder; ^1^H NMR (500 MHz, DMSO-*d*_6_): 6.16 (1H, d, *J* = 2.3 Hz, H-5), 5.53 (1H, d, *J* = 2.3 Hz, H-3), 4.61 (1H, dd, *J* = 8.3, 3.9 Hz, H-9), 4.55 (1H, d, *J* = 2.9 Hz, H-7), 4.14 (1H, dd, *J* = 8.3, 2.9 Hz, H-8), 3.83 (3H, s, H-14), 3.79 (1H, m, H-10), 1.50 (1H, m, H-11a), 1.41 (1H, m, H-11b), 1.35 (1H, m, H-12a), 1.38 (1H, m, H-12b), 1.22 (3H, s, H-16), 1.16 (3H, s, H-17), 0.88 (3H, t, *J* = 7.0 Hz, H-14); ESIMS *m/z*: 391.1 [M + H]^+^, *m/z* 413.1 [M + Na]^+^.

### Quantum Chemical Calculation of NMR Chemical Shifts of Compound 1a

The quantum chemical calculation of NMR chemical shifts of **1a** was performed with DP4+ method (Grimblat et al., 2015). Monte Carlo conformational searches were used to find the conformations using Merck Molecular Force Field (MMFF) by the Spartan's 10 software. The conformers with Boltzmann-population (over 5%) were initially optimized at B3LYP/6-31G(d,p) level in gas. After that, density functional theory (DFT) at the mPWLPW91-SCRF(DMSO)/6-311+G(d,p) level with the PCM solvent continuum model in Gaussian 09 software were used to ^1^H and ^13^C NMR chemical shifts calculations by gauge-independent atomic orbital (GIAO) method. The calculated NMR data of **1a-1** and **1a-2** were averaged by their relative Gibbs free energy and Boltzmann distribution theory. Meanwhile, TMS were calculated by the same protocol and used as reference. The calculated and experimental data were analyzed by the improved probability DP4+ method for isomeric compounds. A significant higher DP4+ probability score suggested the correctness of their configurations.

### ECD Calculation of 1F-1–1F-4

Monte Carlo conformational searches were performed through the Spartan's 10 software with the Merck Molecular Force Field (MMFF). The conformers with Boltzmann-population (over 5%) were initially optimized at B3LYP/6-311+G (d) level. The ECD calculation of all conformers of **1F**-**1**–**1F**-**4** was performed by using time-dependent density functional theory (TD-DFT) at the B3LYP/6-311++G (2d, p) level. Rotatory strengths for a total of 60 excited states were calculated. ECD spectra were generated from dipole-length rotational strengths using Gaussian band shapes with sigma = 0.3 eV by SpecDis 1.6 (University of Würzburg, Würzburg, Germany), and plotted GraphPad Prism 7 (University of California San Diego, USA).

### DNA Topo I Inhibition Bioassay

The topoisomerase I inhibition activity was assayed *in vitro* by assessing the relaxation of supercoiled pBR322 plasmid DNA with camptothecin as the positive control (Hsiang et al., 1989). The 20 μL reaction mixture solution, containing 5 mM dithiothreitol, 0.01% bovine serum albumin, 72 mM KCl, 5 mM MgCl_2_, 5 mM spermidine, 35 mM Tris-HCl (pH 8.0), 1.0 U calf thymus DNA Topo I, 0.5 μg pBR322 plasmid DNA, and 0.2 μL various concentrations of tested compounds, was incubated 30 min at 37°C. The reactions were terminated via adding dye solution (1% SDS, 0.02% bromophenol blue and 50% glycerol). The reaction mixtures were added to 1% agarose gel (1 μL Gelred) and subjected to electrophoresis at the condition of 150 V, 120 mA for 1 h in Tris-borate-EDTA buffer (89 μM). It was observed under ultraviolet condition, and then photographed using the Gel imaging system.

### AChE Inhibition Bioassay

The AChE inhibition activity was determined according to the modified Ellman's method (Ellman, 1958; Ellman et al., 1961). Huperzine A was used as a positive control.

### Antibacterial Bioassay

Antibacterial activity against MRSA was evaluated using a broth microdilution method according to standards and guidelines recommended by Clinical and Laboratory Standards Institute (CLSI) (2012). Methicillin-resistant *Staphylococcus aureus* (ATCC 33591 and ATCC43300) purchased from American Type Culture Collection (ATCC), were used. Vancomycin was used as a positive control.

## Results

### Chemical Epigenetic Manipulation on *C. lunatus* (TA26-46)

**Figure 1:**
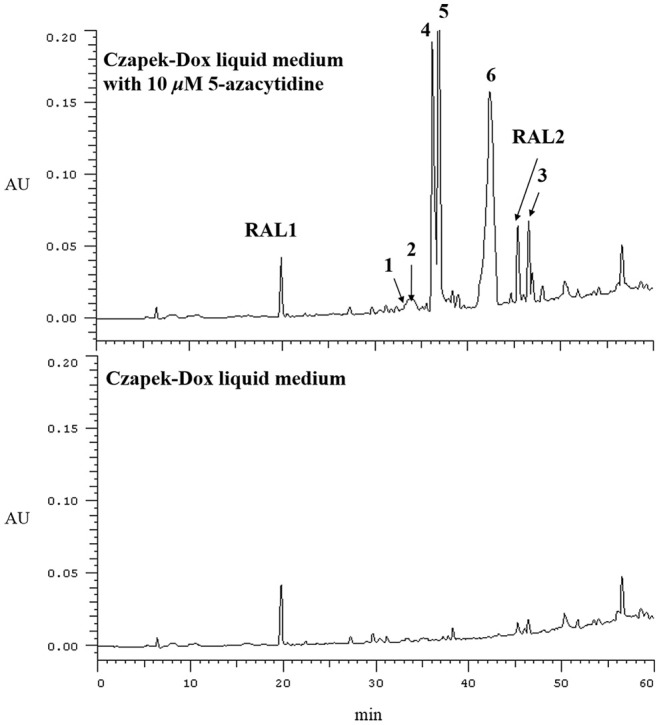
HPLC profiles of EtOAc extracts of *C. lunatus* (TA26-46) cultured in Czapek-Dox liquid medium with 10 μM 5-azacytidine.

**Figure 2:**
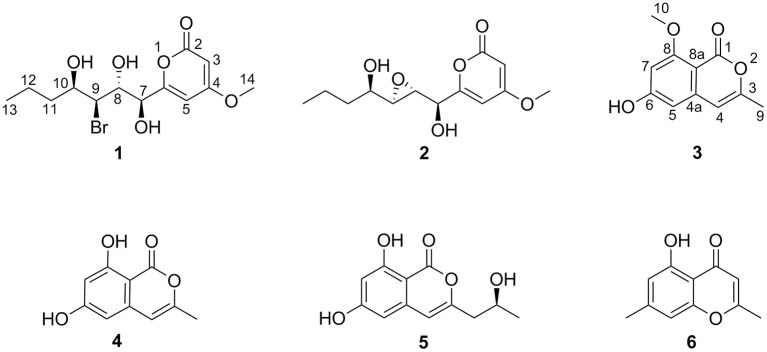
The chemical structures of compounds **1**–**6**.

In our previous reports, Chen et al. (2016) employed 10 μM 5-azacytidine to treat *C. lunatus* (TA26-46), cultivated in starch solid medium to obtain diethylene glycol phthalate esters. Zhang et al. (2014) treated this fungus with 0.01 M sodium butyrate and 10 μM SAHA to harvest brominated RALs. In present study, the chemical epigenetic manipulation on *C. lunatus* (TA26-46) was conducted in Czapek-Dox liquid medium with two DNA methyltransferase inhibitors (5-azacytidine and 2′-deoxy-5-azacytidine) and four histone deacetylase inhibitors (SAHA, SBHA, sodium butyrate and nicotinamide). The fungal culture in the same medium without inhibitor was used as the control. As a result, it was founded that 5- azacytidine and SAHA could result in the significant changes of the secondary metabolites. Further optimized experiments with different concentrations (1, 10, 50, 100, 500, and 1,000 μM) demonstrated that the culture treated with 10 μM 5-azacytidine displayed the most remarkable difference in the secondary metabolites of this fungus (Figure 1). In the HPLC profiles, besides the 14-membered RALs, much more peaks emerged in the culture treated with 10 μM 5-azacytidine compared to the control. Therefore, a scaled-up fermentation with 10 μM 5-azacytidine was carried out. The EtOAc extract of culture was subjected to column chromatography and semi-preparative HPLC, resulting in the isolation of two new α-pyrones, cochliobopyrones A **(1**) and B (**2**), along with three known isocoumarins, 6-hydroxy-8-methoxy-3-methylisocoumarin (**3**), 3-methyl-6,8-dihydroxyisocoumarin (**4**) (Kumagai et al., 1994), (+)-orthosporin (**5**) (Ichihara et al., 1989), and one known chromone altechromone A (**6**) (Kimura et al., 1992) (Figure 2). Two known RALs, cochliomycin E and LL-Z1640-2 (Liu et al., 2014), corresponding to the other two peaks in the HPLC profile (RAL1 and RAL2 in Figure 1), were also obtained from the treated culture.

### Structure Elucidation

**Figure 3:**
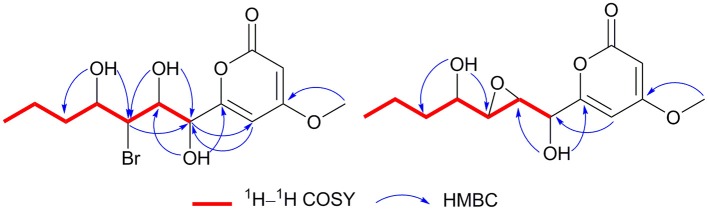
^1^H–^1^H COSY and key HMBC correlations of compounds **1** and **2**.

Cochliobopyrone A (**1**) was obtained as a yellowish oil. Its molecular formula was determined as C_13_H_19_O_6_Br with four degrees of unsaturation on the basis of the HRESIMS. The ratio of [M + Na]^+^ isotopic peaks (1:1) clearly suggested the presence of one bromine atom. In the ^1^H NMR spectrum, two olefinic protons (δ_H_ 6.13 and δ_H_ 5.54), four methine protons (δ_H_ 4.75, δ_H_ 4.12, δ_H_ 4.04, and δ_H_ 3.86), four methylene protons, and two methyl protons (δ_H_ 3.81 and δ_H_ 0.89) were observed (Table 1 and Supporting Information 1). The ^13^C NMR spectroscopic data (Table 1 and Supporting Information 1) displayed the presence of 13 carbons, containing one ester carbonyl carbon, four olefinic carbons, four methine carbons, two methylene carbons and two methyl carbons (one oxygenated). As the presence of one ester carbonyl carbon and four olefinic carbons in the molecule occupied three degrees of unsaturation, **1** should contain one ring. It could be deduced that a disubstituted α-pyrone moiety exists in **1** based on the mutual W-form coupling between two olefinic protons (δ_H_ 6.13, d, *J* = 2.0 Hz and δ_H_ 5.54, d, *J* = 2.0 Hz). The HMBC correlation from H-14 to C-4 indicated that the 4-methoxy was anchored at C-4. These spectroscopic and structure features of α-pyrone moiety were almost identical to the reported 4-methoxy-6-subtituted α-pyrones, nodulisporipyrones B–C from fungus *Nodulisporium* sp. (Zhao et al., 2015). The analysis of contiguous sequence of ^1^H–^1^H COSY correlations from H-7 to H-13 allowed us to establish a spin system from C-7 to C-13 belonging to a seven numbered aliphatic side-chain (Figure 3). Combining with the ^1^H NMR and HSQC, the oxygenated protons at δ_H_ 5.84, δ_H_ 5.22 and δ_H_ 4.70 were deduced as three hydroxyl groups on the side-chain. The HMBC correlations from 10-OH to C-11/C-9, from 8-OH to C-9/C-7, and from 7-OH to C-8 confirmed that these three hydroxyls located at C-7, C-8 and C-10 on the side-chain, respectively. The bromine atom was anchored at C-9 based on the HMBC correlations from H-9 (methine proton) to C-7/C-11, and from 10-OH/8-OH to C-9. The spectroscopic characteristics of the seven numbered aliphatic side-chain in **1** was similar to the side-chain of the reported furan lactone toblerol E from methylobacteria *Methylobacterium extorquens* AM1 (Ueoka et al., 2018), with only difference of bromine substitute in **1** instead of chloride substitute in toblerol E. The HMBC correlations from 7-OH to C-6/C-8, from H-5 to C-7, and from H-7 to C-5 determined that the side-chain was attached to C-6 in **1** (Figure 3). On the basis of the above evidences, the planar structure of **1** was established.

**Figure 4:**
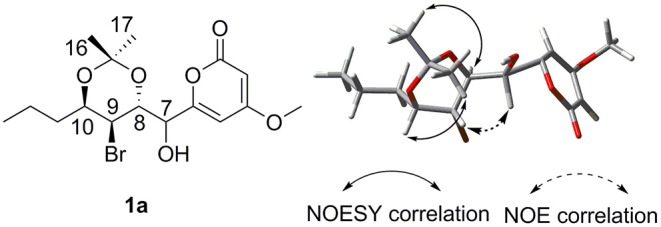
The structure of compound **1a** and its NOESY/NOE correlations.

**Figure 5:**
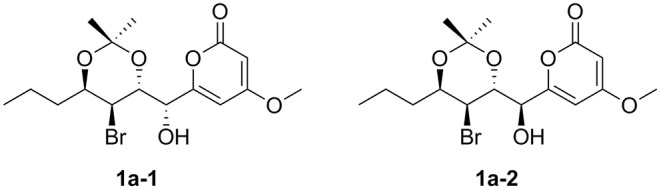
The structures of **1a-1** and **1a-2**.

To assign the relative configuration of **1**, we prepared its acetonide derivative **1a**. The NOESY correlations between H-8 and H-16 and between H-10 and H-17 in **1a** suggested the *anti*-relationship of H-8 and H-10 (Figure 4). The coupling constants of ^3^*J*_H−8−H−9_ = 8.4 Hz and ^3^*J*_H−9−H−10_ = 3.9 Hz indicated the *anti*-relationship between H-8 and H-9 and the *syn*-relationship between H-9 and H-10. The presented NOE correlation between H-7 and H-9 speculated their *syn*-relationship (Figure 4). Two possible diastereoisomers **1a-1** and **1a-2** were deduced for **1a** (Figure 5). The quantum chemical calculations of the NMR shifts for **1a-1** and **1a-2** were performed with DP4+ method. As a result, **1a-2** was identified as the most likely candidate with 100% DP4+ (carbon data) probability (Supporting Informations 2, 3), which was consistent with the aforementioned assumption. Therefore, the relative configuration of **1a** was unambiguously determined as 7S^*^,8R^*^,9S^*^,10R^*^.

**Figure 6:**
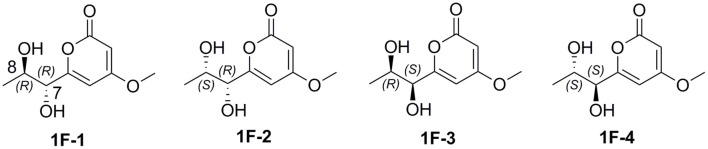
The structures of **1F-1–1F-4**.

**Figure 7:**
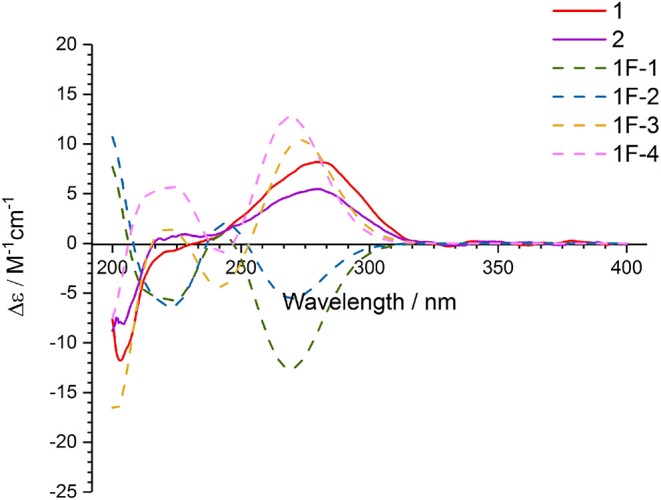
The experimental ECD spectra of **1**–**2** and calculated ECD spectra of **1F-1**–**1F-4**.

In order to determine the absolute configuration of **1**, the Mosher and X-ray methods were attempted but failed. Four fragment molecules of **1**, **1F-1**–**1F-4** were designed with absolute configurations of C-7 and C-8 as 7*R*,8*R*, 7*R*,8*S*, 7*S*,8*R* and 7*S*,8*S*, respectively (Figure 6). Their optimized conformers were subjected to the time-dependent density functional theory (TD-DFT) calculation of the electronic circular dichroism (ECD) spectra at the B3LYP/6-311++G (2d, p) level with PCM in MeOH. **1F-1** and **1F-2** displayed the negative Cotton effects at 270 nm, while **1F-3** and **1F-4** exhibited the positive Cotton effects (Figure 7 and Supporting Information 4). The calculated results suggested that the Cotton effects at 270 nm may be induced by C-7 rather than C-8. The experimental ECD spectra of **1** showed the positive Cotton effect at 270 nm, similar to those of **1F-3** and **1F-4**, suggesting the *S* configuration at C-7 in **1**. Thus, the absolute configuration of **1** was confidently assigned as 7*S*,8*R*,9*S*,10*R*.

Cochliobopyrone B (**2**) was obtained as a white powder. On the basis of the HRESIMS, its molecular formula was established as C_13_H_18_O_6_ (five degrees of unsaturation), less one bromine and one hydrogen compared to **1**. The ^1^H NMR and ^13^C NMR spectroscopic data of **2** were closely related to those of **1** (Table 1). According to one additional degree of unsaturation and the absence of one hydroxyl signal compared to **1**, an epoxy ring could be deduced in **2**. The epoxidation occurred at C-8 and C-9 based on the HMBC correlations from 10-OH to C-9 and from 7-OH to C-8 (Figure 3).

**Figure 8:**
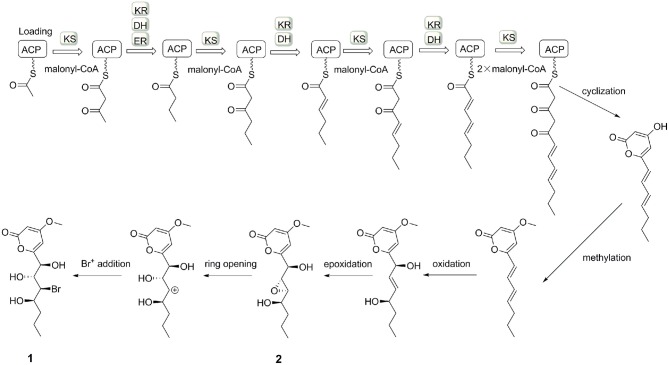
The possible biosynthesis of induced compounds **1** and **2**.

The small coupling constant ^3^*J*_H−8−H−9_ = 2.1 Hz of **2** suggested a *trans*-epoxide (Ueoka et al., 2018). The absolute configuration of C-7 in **2** was designed as *S* according to the positive Cotton effect at 270 nm in its experimental ECD spectrum (Figure 7). Considerations on the same biogenetic pathway as the co-isolated compound **1**, the absolute configuration of C-10 could be proposed as *R*. Based on the plausible biosynthetic pathway from **2** to **1**, the opening of epoxy ring in **2** and addition at C-9 with Br^+^ resulted in the production of **1** (Figure 8). Therefore, the absolute configuration of **2** could be tentatively assigned as 7*S*,8*S*,9*S*,10*R*.

6-Hydroxy-8-methoxy-3-methylisocoumarin (**3**) was obtained as a white powder. Its molecular formula was defined as C_11_H_10_O_4_ by HRESIMS, more than 14 Da compared to the known compound **4**. The ^1^H NMR and ^13^C NMR data both revealed the existence of an additional oxygenated methyl group in **3** (Table S1). The HMBC correlations from H-4/H-5 to C-8a, and from H-10 to C-8 were observed, suggesting that the methoxy group was attached to C-8. It should be pointed that the structure of **3** was listed in SciFinder Scholar with the CAS Registry Number of 2090467-41-5, but with no reference and experimental data. Thus, it was the first time to report the spectroscopic data for **3**.

### The Induced Pathway of *C. lunatus* (TA26-46)

In our previous study, *C. lunatus* (TA26-46) was proven to produce a series of RALs, which suggested the presence of HR-PKS and NR-PKS enzymes (Zhou et al., 2010, 2012). When *C. lunatus* (TA26-46) was subjected to chemical epigenetic manipulation with DNA methyltransferase inhibitor 5-azacytidine, new types of polyketides were harvested, including two new α-pyrones, three known isocoumarins, and one known chromone. It could be assumed that DNA methyltransferase inhibitor 5-azacytidine may induce other PKS pathways in this strain to produce new types of polyketides. The production of two α-pyrones, **1** and **2**, may be induced through a type III PKS pathway (Figure 8), similar to that of the reported α-pyrones (Jeong et al., 2005; Zhou et al., 2012; Ueoka et al., 2018). Our study provides a successful case for chemical epigenetic manipulation to activate the silent metabolic pathway and acquire cryptic metabolites for the sake of enhancing chemical diversity and novelty of secondary metabolites from marine-derived fungi.

### Bioassays of Compounds

All of the isolated compounds (**1**–**6**) were evaluated for their topoisomerase I (Topo I) inhibitory, acetylcholinesterase inhibitory, and antibacterial activities. None of them showed any obvious antibacterial or acetylcholinesterase inhibitory activity. Compounds **1** and **2** exhibited Topo I inhibitory activities with the MIC values of 100 and 50 μM, respectively. Natural products with α-pyrone moiety have been expected as the valuable precursors of potential antitumor agents targeting Topo I (Holla et al., 2017; Sang et al., 2017). In this study, only two α-pyrones were obtained via chemical epigenetic manipulation from *C. lunatus* (TA26-46). More strategies and approaches are prospected to be applied to this fungal strain to mine more α-pyrone derivatives to investigate their Topo I inhibitory activities systematically.

## Conclusion

In summary, the zoanthid-derived fungus *C. lunatus* (TA26-46) was effectively induced by chemical epigenetic manipulation with 10 μM 5-azacytidine to produce new types of metabolites. Six induced metabolites were obtained from the culture with DNA methyltransferase inhibitor, 5-azacytidine, including two new α-pyrones, three known isocoumarins, and one known chromone. Based on the above results, it could be concluded that chemical epigenetic manipulation should be a feasible strategy to activate the silent metabolic pathway and tap new secondary metabolites from marine derived-fungi.

## Data Availability Statement

All datasets generated for this study are included in the article/Supplementary Material.

## Author Contributions

C-YW and C-LS conceived of and proposed the idea. J-SW designed the study. J-SW, X-HS, Y-HZ, and J-YY performed the experiments. J-SW and Y-HZ participated in data analysis. X-MF, XL, K-XC, and Y-WG contributed to writing, revising and proof-reading the manuscript. All authors read and approved the final manuscript.

### Conflict of Interest

The authors declare that the research was conducted in the absence of any commercial or financial relationships that could be construed as a potential conflict of interest.
