# Supplementary material for: Co-cultivation With 5-Azacytidine Induced New Metabolites From the Zoanthid-Derived Fungus Cochliobolus lunatus
Source: Front Chem. 2019 Nov 8;7:763. doi: 10.3389/fchem.2019.00763 (PMC6857680; doi:10.3389/fchem.2019.00763)
Supplement: Supplementary file 1 [file Table_1.docx]

Co-cultivation with 5-azacytidine induced new metabolites from the zoanthid-derived fungus *Cochliobolus lunatus*

Jing-Shuai Wu ^1,2^, Xiao-Hui Shi ^1,2^, Ya-Hui Zhang ^1,2^, Jia-Yin Yu ^1,2^, Xiu-Mei Fu ^1,2^, Xin Li ^1,2^, Kai-Xian Chen ^3^, Yue-Wei Guo ^3^, Chang-Lun Shao ^1,2*^, Chang-Yun Wang ^1,2,4*^

^1^ Key Laboratory of Marine Drugs, The Ministry of Education of China, School of Medicine and Pharmacy, Ocean University of China, Qingdao 266003, People’s Republic of China

^2^ Laboratory for Marine Drugs and Bioproducts, Qingdao National Laboratory for Marine Science and Technology, Qingdao 266237, People’s Republic of China

^3^ Open Studio for Druggability Research of Marine Natural Products, Pilot National Laboratory for Marine Science and Technology (Qingdao), Qingdao, 266237, People’s Republic of China

^4^ Institute of Evolution & Marine Biodiversity, Ocean University of China, Qingdao 266003, People’s Republic of China

^*^**Corresponding authors:**

*E-mail address:* changyun@ouc.edu.cn (C.-Y. Wang) or shaochanglun@163.com (C.-L. Shao)

**List of supporting information**

**Supporting information 1.** 1D, 2D NMR spectra and HRESIMS of compounds **1**–**3**.

**Figure S1.** ^1^H NMR (500 MHz, DMSO-*d*_6_) spectrum of compound **1**

**Figure S2.** ^13^C NMR (125 MHz, DMSO-*d*_6_) spectrum of compound **1**

**Figure S3.** HSQC (DMSO-*d*_6_) spectrum of compound **1**

**Figure S4.** ^1^H–^1^H COSY (DMSO-*d*_6_) spectrum of compound **1**

**Figure S5.** HMBC (DMSO-*d*_6_) spectrum of compound **1**

**Figure S6.** HRESIMS spectrum of compound **1**

**Figure S7.** ^1^H NMR (500 MHz, DMSO-*d*_6_) spectrum of compound **1a**

**Figure S8.** ^13^C NMR (125 MHz, DMSO-*d*_6_) spectrum of compound **1a**

**Figure S9.** ^1^H–^1^H COSY (DMSO-*d*_6_) spectrum of compound **1a**

**Figure S10.** NOESY (DMSO-*d*_6_) spectrum of compound **1a**

**Figure S11.** NOE (500 MHz, DMSO-*d*_6_) spectrum of compound **1a**

**Figure S12.** ESIMS spectrum of compound **1a**

**Figure S13.** ^1^H NMR (500 MHz, DMSO-*d*_6_) spectrum of compound **2**

**Figure S14.** ^13^C NMR (125 MHz, DMSO-*d*_6_) spectrum of compound **2**

**Figure S15.** HSQC (DMSO-*d*_6_) spectrum of compound **2**

**Figure S16.** ^1^H–^1^H COSY (DMSO-*d*_6_) spectrum of compound **2**

**Figure S17.** HMBC (DMSO-*d*_6_) spectrum of compound **2**

**Figure S18.** NOESY (DMSO-*d*_6_) spectrum of compound **2**

**Figure S19.** HRESIMS spectrum of compound **2**

**Figure S20.** ^1^H NMR (500 MHz, DMSO-*d*_6_) spectrum of compound **3**

**Figure S21.** ^13^C NMR (125 MHz, DMSO-*d*_6_) spectrum of compound **3**

**Figure S22.** HSQC (DMSO-*d*_6_) spectrum of compound **3**

**Figure S23.** HMBC (DMSO-*d*_6_) spectrum of compound **3**

**Figure S24.** HRESIMS spectrum of compound **3**

**Table S1.** ^1^H NMR and ^13^C NMR data for compound **3**

**Supporting information 2.** The NMR calculation results of **1a-1** and **1a-2** with DP4+ method

**Supporting information 3.** The DP4+ probability scores of **1a-1** and **1a-2**

**Supporting information 4.** The ECD calculation results of **1F-1**–**1F-4**

**Supporting information 1.** 1D, 2D NMR spectra and HRESIMS of compounds **1**–**3**.

**
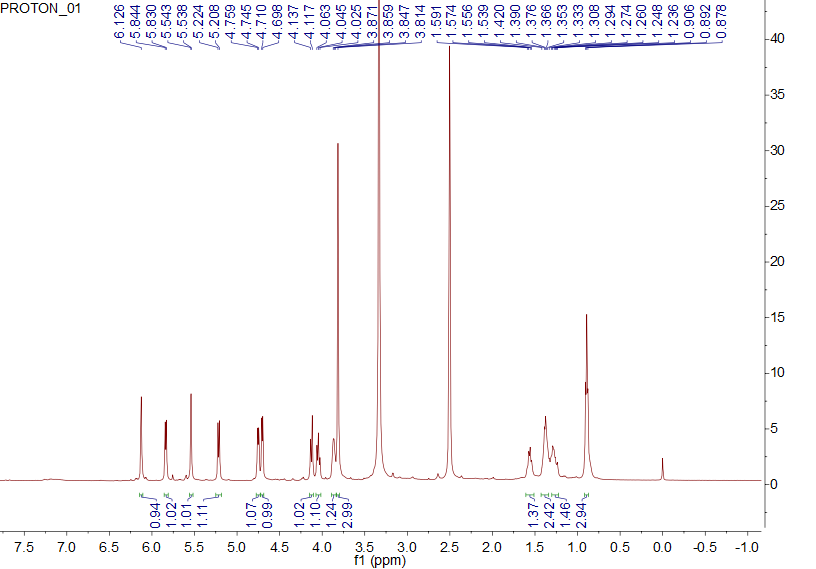
**

**Figure S1.** ^1^H NMR (500 MHz, DMSO-*d*_6_) spectrum of compound **1**

**
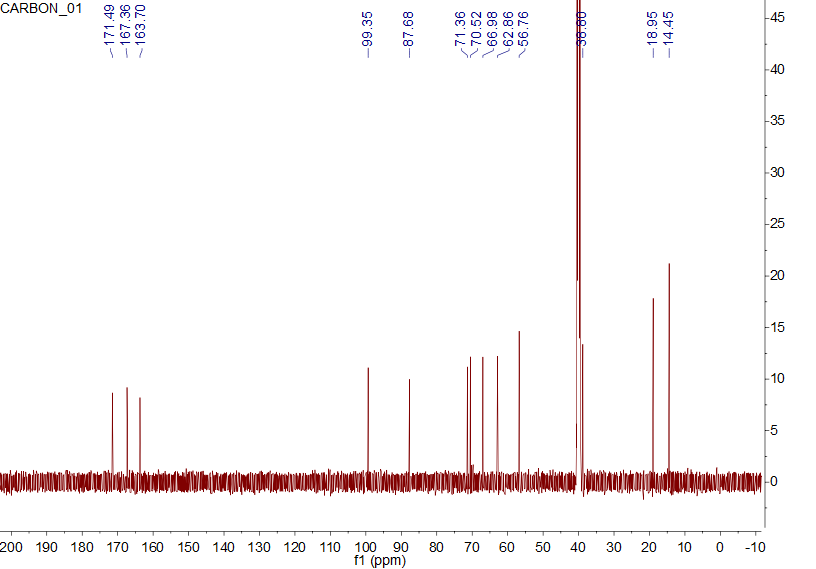
**

**Figure S2.** ^13^C NMR (125 MHz, DMSO-*d*_6_) spectrum of compound **1**

**
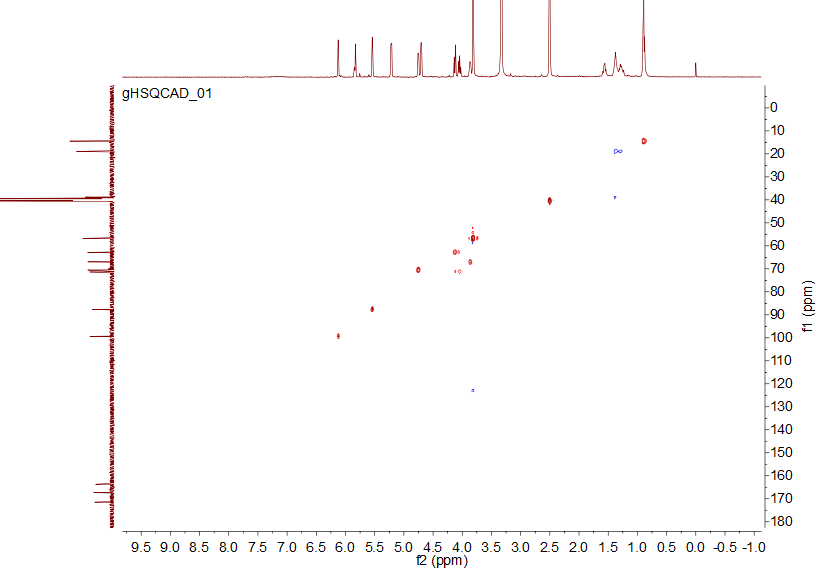
**

**Figure S3.** HSQC (DMSO-*d*_6_) spectrum of compound **1**

**
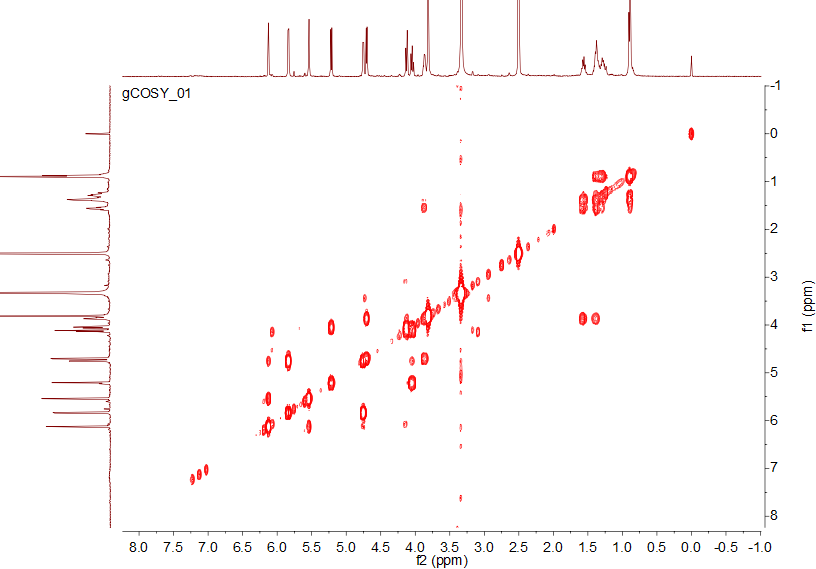
**

**Figure S4.** ^1^H–^1^H COSY (DMSO-*d*_6_) spectrum of compound **1**

**
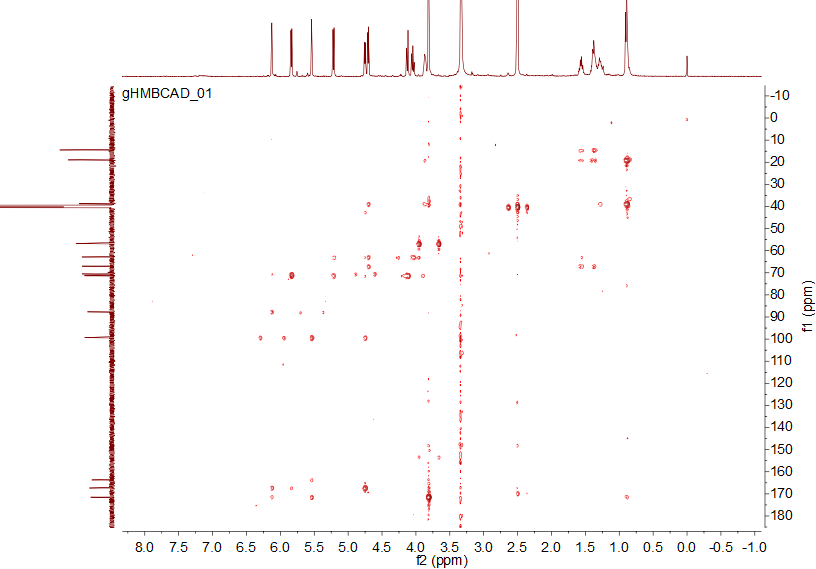
**

**Figure S5.** HMBC (DMSO-*d*_6_) spectrum of compound **1**

**Figure S6.** HRESIMS spectrum of compound **1**


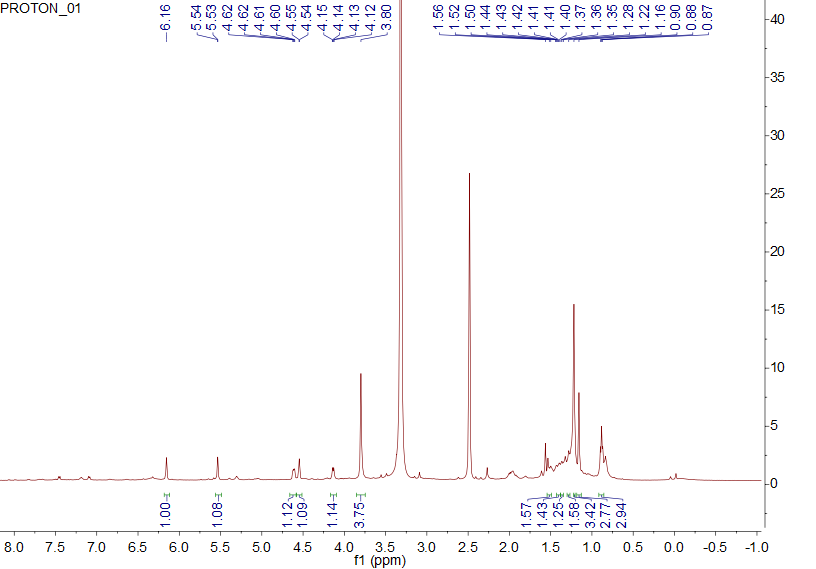


**Figure S7.** ^1^H NMR (500 MHz, DMSO-*d*_6_) spectrum of compound **1a**

**
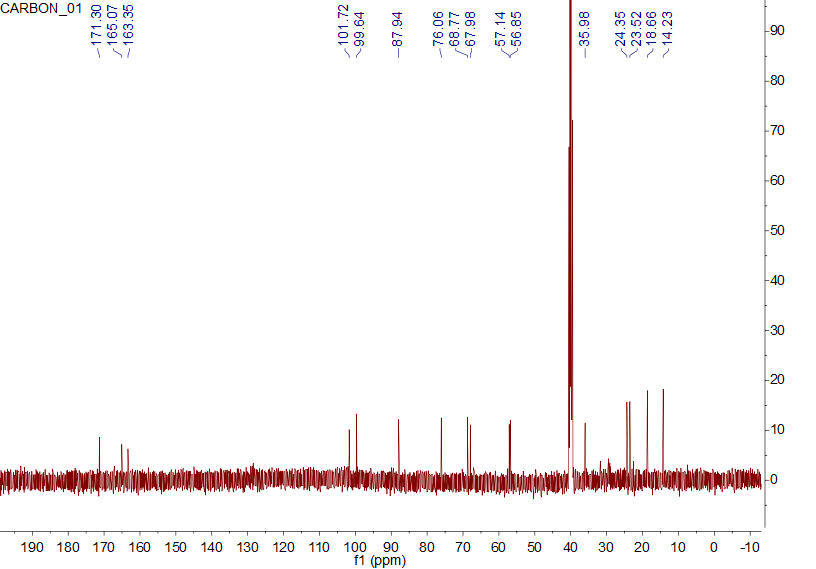
**

**Figure S8.** ^13^C NMR (125 MHz, DMSO-*d*_6_) spectrum of compound **1a**


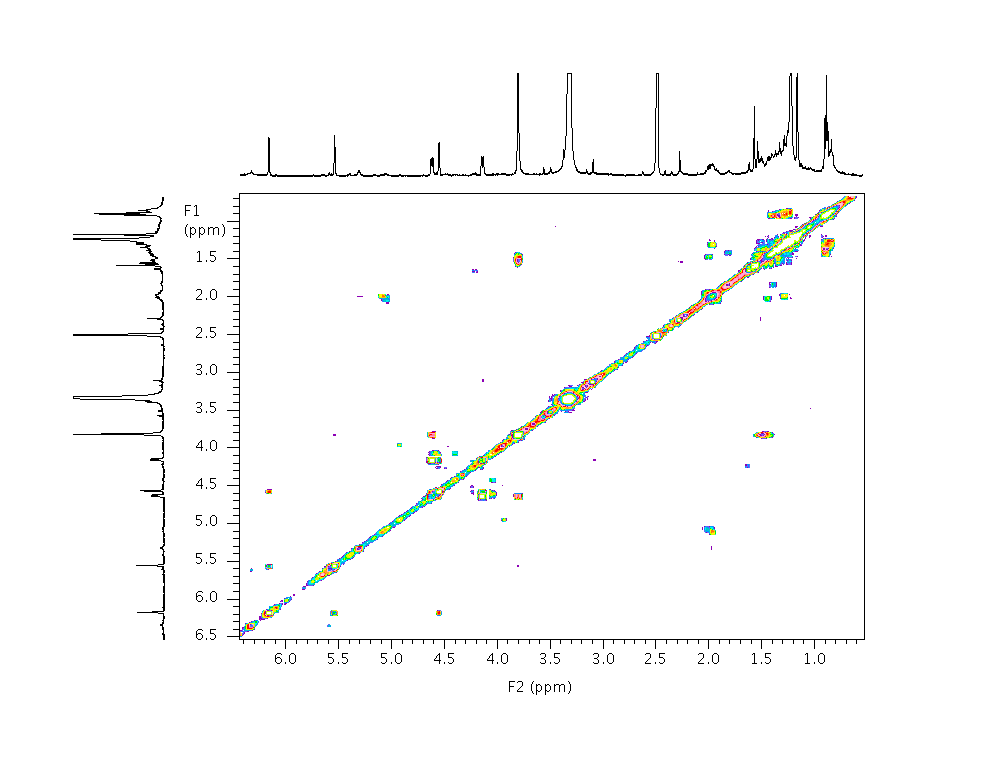


**Figure S9.** ^1^H–^1^H COSY (DMSO-*d*_6_) spectrum of compound **1a**


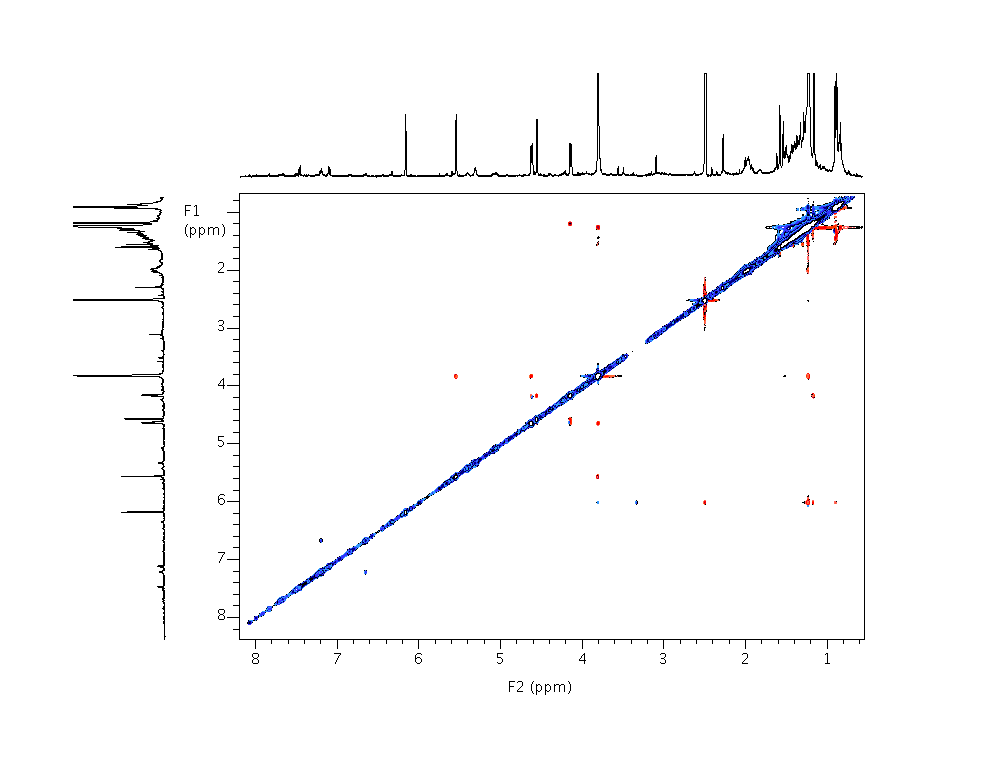


**Figure S10.** NOESY (DMSO-*d*_6_) spectrum of compound **1a**

**
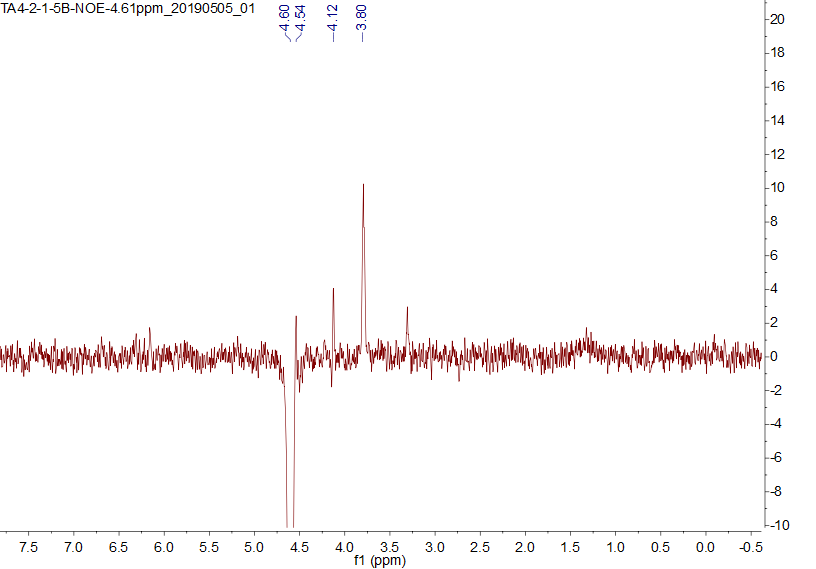
**

**Figure S11.** NOE (500 MHz, DMSO-*d*_6_) spectrum of compound **1a**

**
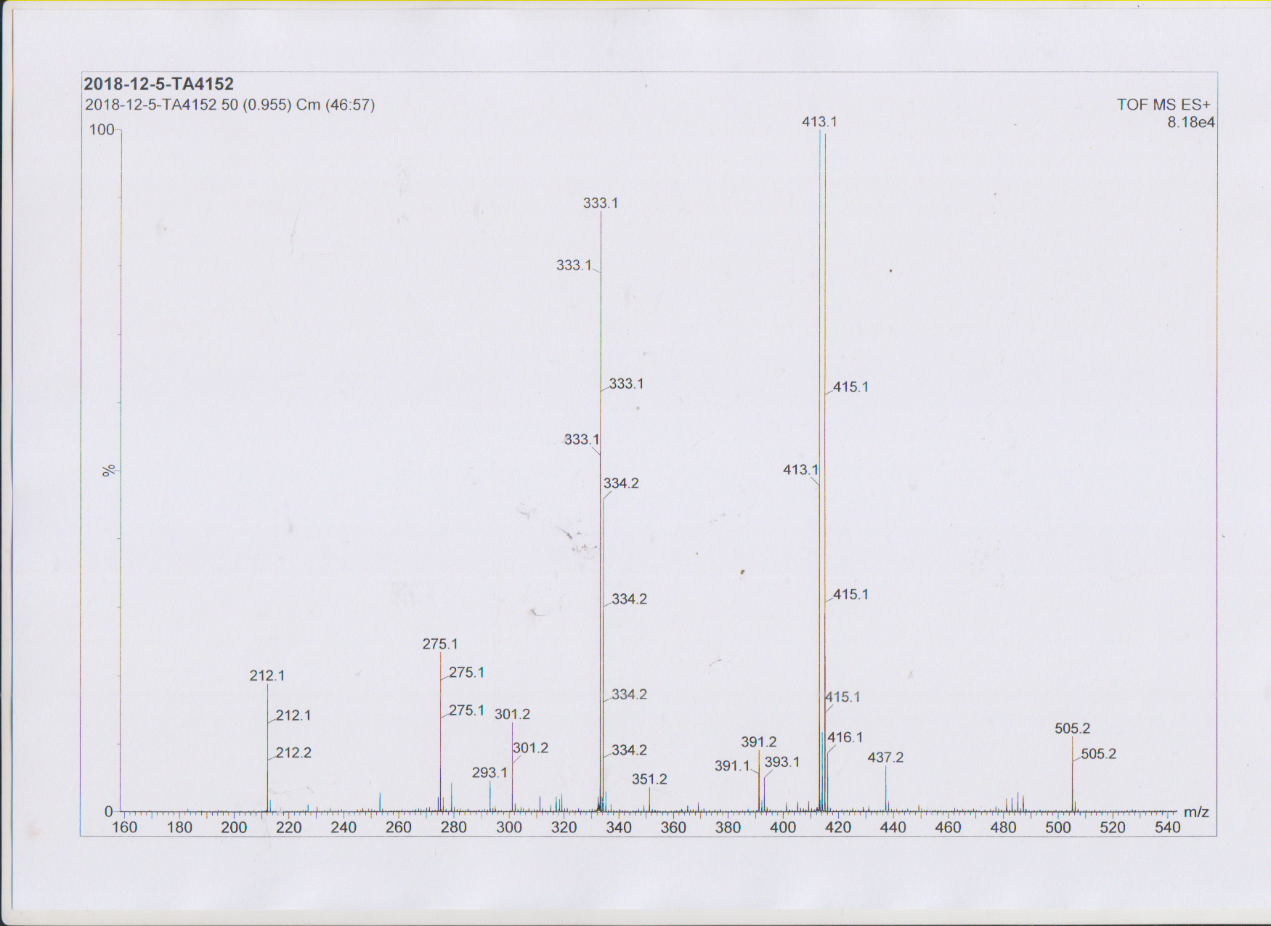
**

**Figure S12.** ESIMS spectrum of compound **1a**


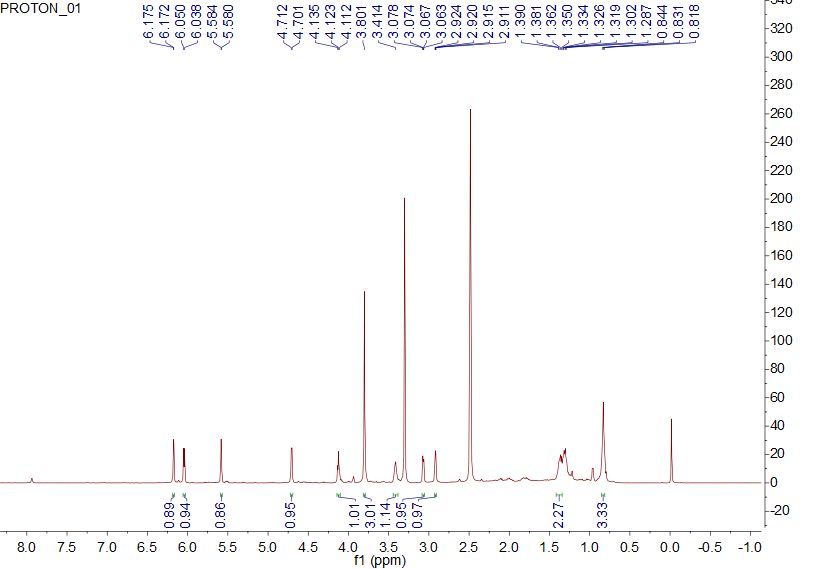


**Figure S13.** ^1^H NMR (500 MHz, DMSO-*d*_6_) spectrum of compound **2**

**
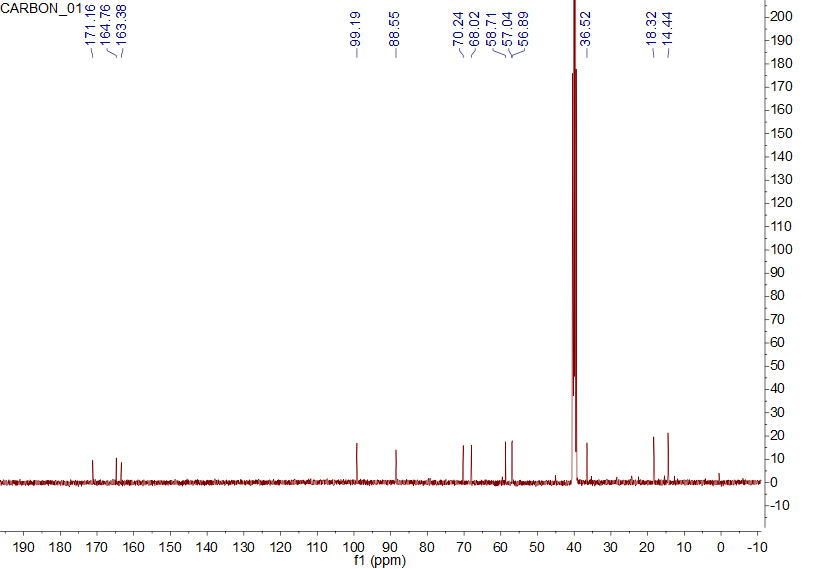
**

**Figure S14.** ^13^C NMR (125 MHz, DMSO-*d*_6_) spectrum of compound **2**

**
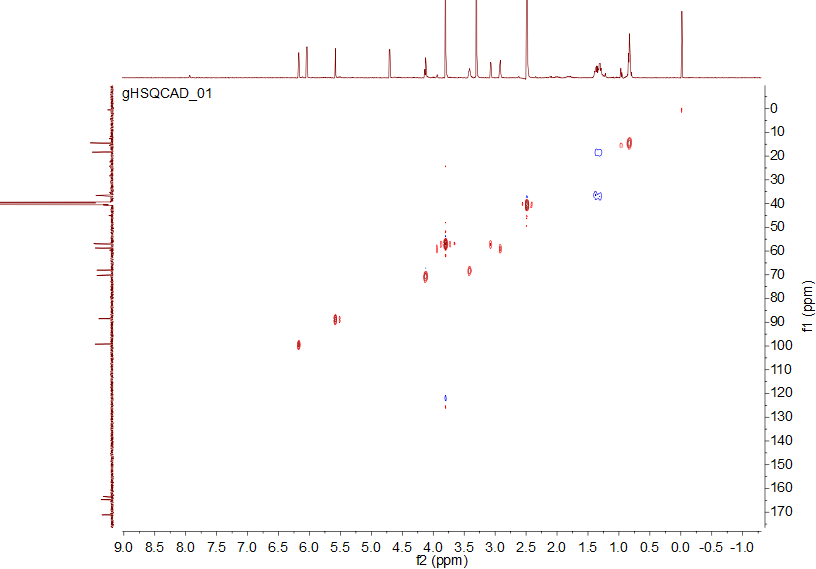
**

**Figure S15.** HSQC (DMSO-*d*_6_) spectrum of compound **2**

**
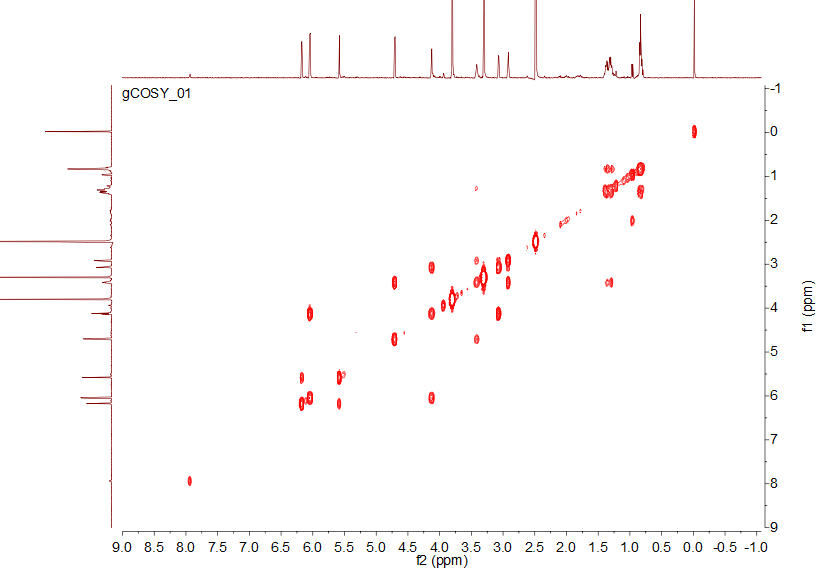
**

**Figure S16.** ^1^H–^1^H COSY (DMSO-*d*_6_) spectrum of compound **2**

**
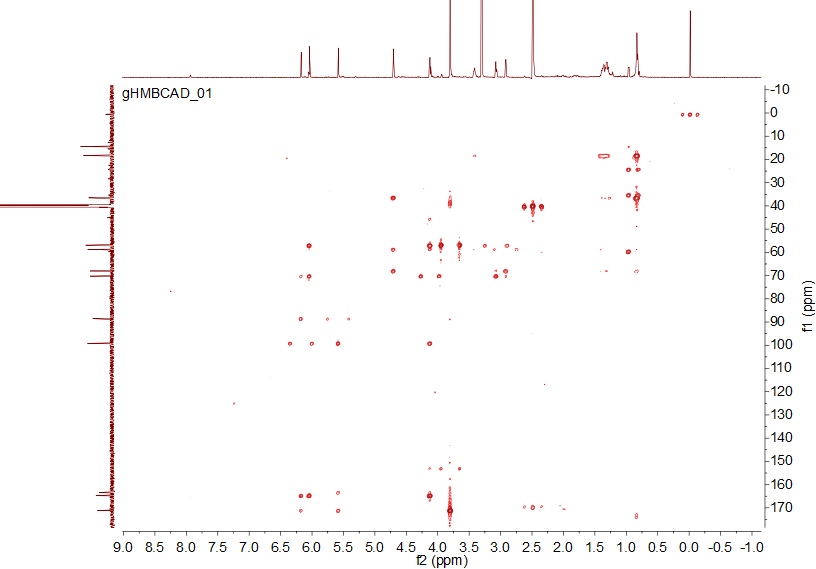
**

**Figure S17.** HMBC (DMSO-*d*_6_) spectrum of compound **2**

**
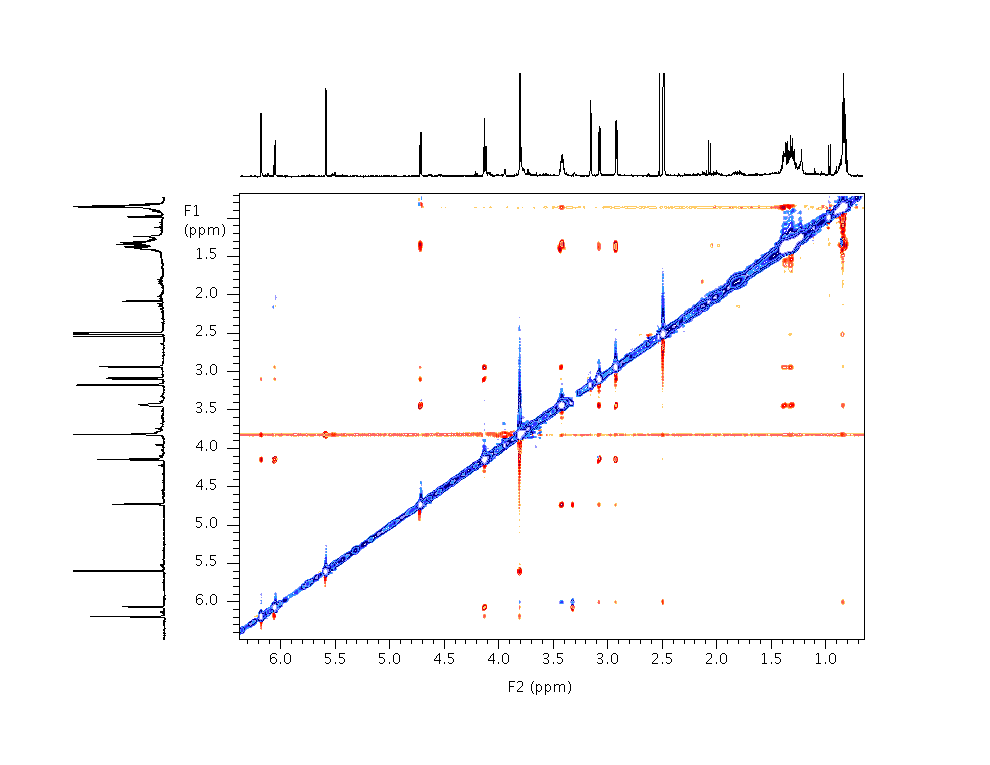
**

**Figure S18.** NOESY (DMSO-*d*_6_) spectrum of compound **2**

**Figure S19.** HRESIMS spectrum of compound **2**

**
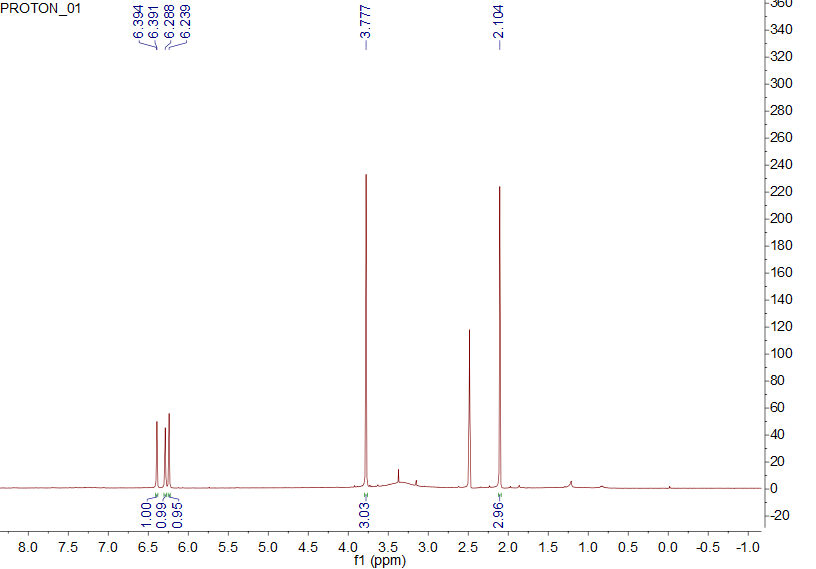
**

**Figure S20.** ^1^H NMR (500 MHz, DMSO-*d*_6_) spectrum of compound **3**

**
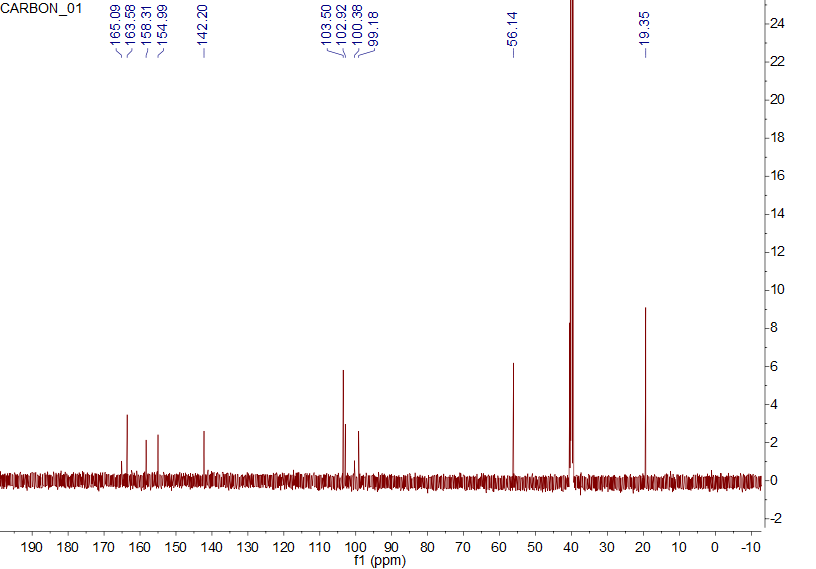
**

**Figure S21.** ^13^C NMR (125 MHz, DMSO-*d*_6_) spectrum of compound **3**

**
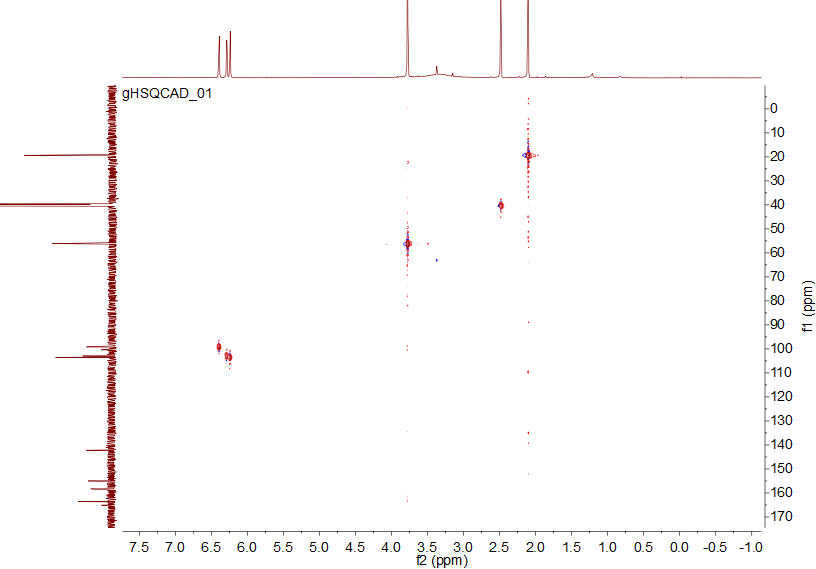
**

**Figure S22.** HSQC (DMSO-*d*_6_) spectrum of compound **3**

**
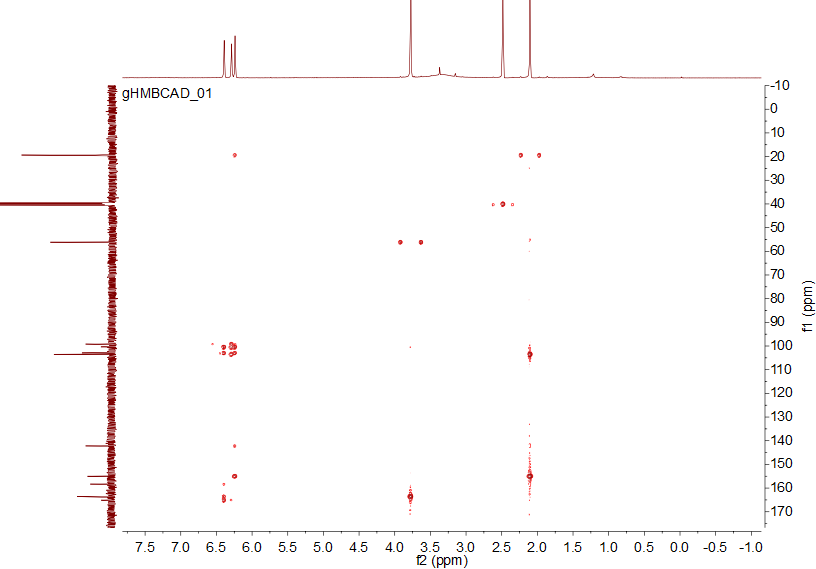
**

**Figure S23.** HMBC (DMSO-*d*_6_) spectrum of compound **3**

**Figure S24.** HRESIMS spectrum of compound **3**

**Table S1.** ^1^H NMR and ^13^C NMR Data for compound **3**

| position | *δ*_C_*^a^*, type | *δ*_H_*^a^* (*J* in Hz) |
| --- | --- | --- |
| 1 | 165.1, C |  |
| 3 | 155.0, C |  |
| 4 | 103.5, CH | 6.24, s |
| 4a | 142.2, C |  |
| 5 | 102.9, CH | 6.29, d, (1.5) |
| 6 | 158.3, C |  |
| 7 | 99.2, CH | 6.39, d, (1.5) |
| 8 | 163.6, C |  |
| 8a | 100.4, C |  |
| 9 | 19.4, CH_3_ | 2.10, s |
| 10 | 56.8, CH_3_ | 3.78, s |
| *^a^* 500 MHz for ^1^H NMR and 125 MHz for ^13^C NMR in DMSO-*d*_6_. | | |
