# Supplementary material for: Co-cultivation With 5-Azacytidine Induced New Metabolites From the Zoanthid-Derived Fungus Cochliobolus lunatus
Source: Front Chem. 2019 Nov 8;7:763. doi: 10.3389/fchem.2019.00763 (PMC6857680; doi:10.3389/fchem.2019.00763)
Supplement: Supplementary file 2 [file Table_2.DOCX]

**Supporting information 2.** The NMR calculation results of **1a-1** and **1a-2** with DP4+ method

1. The ^1^H NMR calculation results

Table 1 The ^1^H NMR calculation results

| position | *δ*_H_ | *Cald-***1a-1** | *Cal-***1a-2** |
| --- | --- | --- | --- |
| 3 | 5.53 | 5.01 | 5.64 |
| 5 | 6.16 | 5.86 | 6.23 |
| 7 | 4.55 | 4.36 | 4.56 |
| 8 | 4.14 | 3.81 | 4.21 |
| 9 | 4.61 | 4.34 | 4.55 |
| 10 | 3.79 | 3.32 | 3.75 |
| 11 | 1.50 | 1.35 | 1.45 |
|  | 1.41 | 1.20 | 1.46 |
| 12 | 1.35 | 1.22 | 1.36 |
|  | 1.38 | 1.14 | 1.33 |
| 13 | 0.88 | 0.89 | 0.85 |
| 14 | 3.83 | 3.57 | 3.86 |
| 16 | 1.22 | 1.12 | 1.24 |
| 17 | 1.16 | 1.17 | 1.15 |

2. The ^13^C NMR calculation results

Table 2 The ^13^C NMR calculation results

| position | *δ*_C_ | *Cald-***1a-1** | *Cal-***1a-2** | |
| --- | --- | --- | --- | --- |
| 2 | 1*63.4* | 164.2185 | 163.8843 | |
| 3 | 87.9 | 88.85145 | 90.20794 | |
| 4 | 171.3 | 175.9147102.1674 | 175.7881 | |
| 5 | 99.6 | 102.1674 | 103.0021 | |
| 6 | 165.4 | 174.4943 | 169.2887 | |
| 7 | 68.0 | 72.15426 | 73.26026 | |
| 8 | 76.1 | 76.82837 | 79.25392 |  |
| 9 | 57.1 | 69.52434 | 66.60138 |  |
| 10 | 68.8 | 72.96954 | 72.36592 |  |
| 11 | 30.0 | 37.82692 | 36.7455 |  |
| 12 | 18.7 | 22.4508 | 21.47331 |  |
| 13 | 14.5 | 14.41381 | 14.06977 |  |
| 14 | 56.8 | 54.77449 | 54.64573 |  |
| 15 | 101.7 | 105.8187 | 106.0631 |  |
| 16 | 23.5 | 21.66043 | 22.90615 |  |
| 17 | 24.4 | 23.28771 | 23.079 |  |

3. For **1a-1**

Table S3.1. Gibbs free energies*^a^* and equilibrium populations*^b^* of low-energy conformers of **1a-1**.

| Conformers | In gas | |
| --- | --- | --- |
|  | G | *P* (%) |
| **1a-1-1** | -2288090.43212646 | 59.72 |
| **1a-1-2** | -2288089.93764858 | 25.90 |
| **1a-1-3** | -2288089.30888356 | 8.95 |
| **1a-1-4** | -2288088.80624805 | 3.83 |
| **1a-1-5** | -2288088.29043483 | 1.60 |
| *^a^*B3LYP/6-31G(d,p), in kcal/mol. *^b^*From G values at 298.15K. | | |

Table S3.2. Cartesian coordinates for the low-energy reoptimized MMFF conformers of **1a-1** at B3LYP/6-31G(d,p) level of theory in gas.

**1a-1-1**


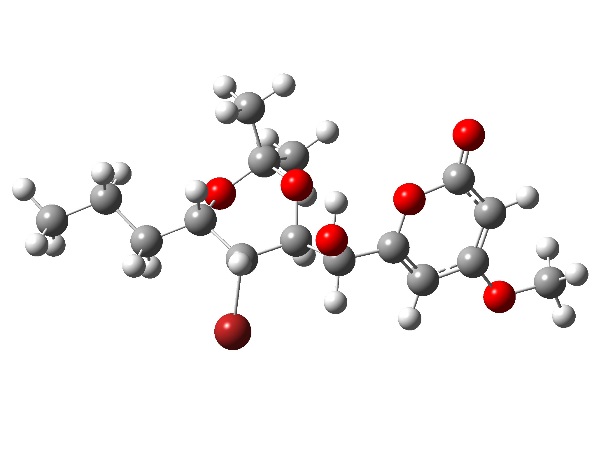
E = =-3649.2276675 a.u

| **1a-1-1** | | Standard Orientation  (Ångstroms) | | | |
| --- | --- | --- | --- | --- | --- |
| Center number | Atomic number | Atomic Type | X | Y | Z |
| 1. | 8. | 0. | -2.239326 | -0.730396 | 0.892533 |
| 2. | 6. | 0. | -2.203065 | -0.519765 | -0.439134 |
| 3. | 6. | 0. | -3.280787 | -0.101738 | -1.138108 |
| 4. | 6. | 0. | -4.505210 | 0.127869 | -0.424985 |
| 5. | 6. | 0. | -4.568576 | -0.071256 | 0.929825 |
| 6. | 6. | 0. | -3.416020 | -0.511199 | 1.670570 |
| 7. | 8. | 0. | -3.323166 | -0.712347 | 2.857718 |
| 8. | 6. | 0. | -0.847190 | -0.832104 | -1.045045 |
| 9. | 6. | 0. | 0.294948 | -0.187170 | -0.247521 |
| 10. | 6. | 0. | 1.682880 | -0.632058 | -0.738333 |
| 11. | 6. | 0. | 2.695518 | 0.500938 | -0.466247 |
| 12. | 6. | 0. | 4.135190 | 0.083417 | -0.189699 |
| 13. | 6. | 0. | 5.108268 | 1.267335 | -0.144549 |
| 14. | 6. | 0. | 6.542085 | 0.836903 | 0.176815 |
| 15. | 8. | 0. | -0.789524 | -0.444720 | -2.405323 |
| 16. | 8. | 0. | 2.238588 | 1.281413 | 0.641222 |
| 17. | 8. | 0. | -5.525887 | 0.539328 | -1.202442 |
| 18. | 8. | 0. | 0.177906 | 1.220797 | -0.466387 |
| 19. | 35. | 0. | 2.096066 | -2.361010 | 0.160727 |
| 20. | 6. | 0. | -6.788453 | 0.787112 | -0.585271 |
| 21. | 6. | 0. | 1.032631 | 2.000929 | 0.392976 |
| 22. | 6. | 0. | 1.294341 | 3.308425 | -0.351625 |
| 23. | 6. | 0. | 0.375921 | 2.224978 | 1.751666 |
| 24. | 1. | 0. | -3.227058 | 0.037921 | -2.207932 |
| 25. | 1. | 0. | -5.463010 | 0.090436 | 1.514020 |
| 26. | 1. | 0. | -0.697604 | -1.918514 | -1.015920 |
| 27. | 1. | 0. | 0.185378 | -0.431474 | 0.811926 |
| 28. | 1. | 0. | 1.666239 | -0.885802 | -1.798371 |
| 29. | 1. | 0. | 2.684433 | 1.119923 | -1.377098 |
| 30. | 1. | 0. | 4.441543 | -0.629826 | -0.965268 |
| 31. | 1. | 0. | 4.158338 | -0.463519 | 0.758797 |
| 32. | 1. | 0. | 5.090497 | 1.792219 | -1.109858 |
| 33. | 1. | 0. | 4.757042 | 1.985051 | 0.604673 |
| 34. | 1. | 0. | 6.925567 | 0.134756 | -0.572214 |
| 35. | 1. | 0. | 7.218864 | 1.696797 | 0.203559 |
| 36. | 1. | 0. | 6.596260 | 0.340451 | 1.151993 |
| 37. | 1. | 0. | -0.691754 | 0.520986 | -2.396854 |
| 38. | 1. | 0. | -7.179014 | -0.119205 | -0.109663 |
| 39. | 1. | 0. | -6.712832 | 1.586490 | 0.160201 |
| 40. | 1. | 0. | -7.456256 | 1.096959 | -1.388921 |
| 41. | 1. | 0. | 1.908638 | 3.969092 | 0.264618 |
| 42. | 1. | 0. | 1.812191 | 3.125340 | -1.296365 |
| 43. | 1. | 0. | 0.348435 | 3.809880 | -0.572592 |
| 44. | 1. | 0. | -0.538149 | 2.811781 | 1.633523 |
| 45. | 1. | 0. | 0.124422 | 1.273624 | 2.225430 |
| 46. | 1. | 0. | 1.061928 | 2.763690 | 2.410299 |

**1a-1-2**


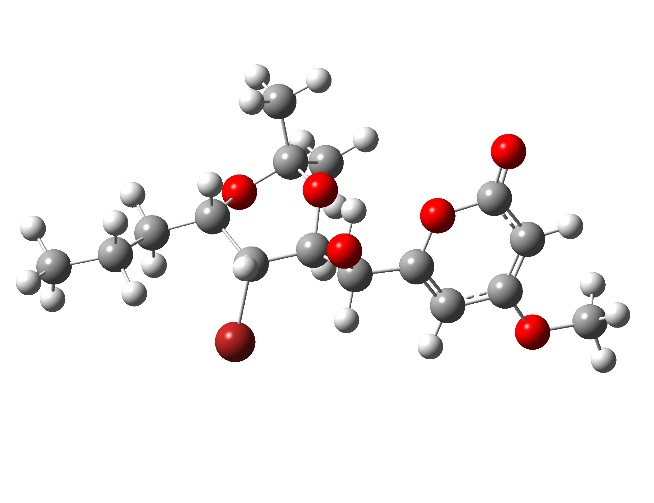
 E =-3649.2270143 a.u

| **1a-1-2** | | Standard Orientation  (Ångstroms) | | | |
| --- | --- | --- | --- | --- | --- |
| Center number | Atom number | Type | X | Y | Z |
| 1. | 8. | 0. | -2.202877 | -0.809095 | 0.852278 |
| 2. | 6. | 0. | -2.112775 | -0.570790 | -0.472242 |
| 3. | 6. | 0. | -3.185180 | -0.245363 | -1.226436 |
| 4. | 6. | 0. | -4.464028 | -0.147716 | -0.581649 |
| 5. | 6. | 0. | -4.583237 | -0.377814 | 0.764458 |
| 6. | 6. | 0. | -3.436829 | -0.719310 | 1.564173 |
| 7. | 8. | 0. | -3.391661 | -0.933860 | 2.751745 |
| 8. | 6. | 0. | -0.700739 | -0.740305 | -1.001724 |
| 9. | 6. | 0. | 0.325376 | 0.003231 | -0.134981 |
| 10. | 6. | 0. | 1.776110 | -0.308454 | -0.540719 |
| 11. | 6. | 0. | 2.661366 | 0.912905 | -0.207332 |
| 12. | 6. | 0. | 4.116986 | 0.647897 | 0.162976 |
| 13. | 6. | 0. | 4.944253 | 0.010358 | -0.960737 |
| 14. | 6. | 0. | 6.413405 | -0.172323 | -0.568052 |
| 15. | 8. | 0. | -0.604083 | -0.332610 | -2.353586 |
| 16. | 8. | 0. | 2.066863 | 1.635316 | 0.873579 |
| 17. | 8. | 0. | -5.474925 | 0.179798 | -1.410243 |
| 18. | 8. | 0. | 0.090204 | 1.395362 | -0.357241 |
| 19. | 35. | 0. | 2.289248 | -1.992061 | 0.393509 |
| 20. | 6. | 0. | -6.787174 | 0.296096 | -0.861750 |
| 21. | 6. | 0. | 0.817943 | 2.243637 | 0.553515 |
| 22. | 6. | 0. | 1.005239 | 3.574640 | -0.171851 |
| 23. | 6. | 0. | 0.062770 | 2.398476 | 1.869947 |
| 24. | 1. | 0. | -3.085421 | -0.080999 | -2.289426 |
| 25. | 1. | 0. | -5.520216 | -0.313665 | 1.298749 |
| 26. | 1. | 0. | -0.447285 | -1.807363 | -0.970905 |
| 27. | 1. | 0. | 0.176154 | -0.258627 | 0.915316 |
| 28. | 1. | 0. | 1.845461 | -0.562330 | -1.598287 |
| 29. | 1. | 0. | 2.645702 | 1.535014 | -1.116063 |
| 30. | 1. | 0. | 4.138679 | 0.012709 | 1.054741 |
| 31. | 1. | 0. | 4.551710 | 1.613826 | 0.445474 |
| 32. | 1. | 0. | 4.517744 | -0.965286 | -1.220423 |
| 33. | 1. | 0. | 4.881403 | 0.633481 | -1.863602 |
| 34. | 1. | 0. | 6.507914 | -0.820589 | 0.309837 |
| 35. | 1. | 0. | 6.987796 | -0.627258 | -1.381031 |
| 36. | 1. | 0. | 6.882676 | 0.787330 | -0.323829 |
| 37. | 1. | 0. | -0.597060 | 0.637798 | -2.332718 |
| 38. | 1. | 0. | -7.116694 | -0.652525 | -0.423907 |
| 39. | 1. | 0. | -6.827320 | 1.082662 | -0.100132 |
| 40. | 1. | 0. | -7.435971 | 0.560050 | -1.696743 |
| 41. | 1. | 0. | 1.528748 | 4.279419 | 0.478383 |
| 42. | 1. | 0. | 1.584955 | 3.443753 | -1.088970 |
| 43. | 1. | 0. | 0.033117 | 3.996275 | -0.440652 |
| 44. | 1. | 0. | -0.892533 | 2.899964 | 1.697483 |
| 45. | 1. | 0. | -0.128324 | 1.425059 | 2.326827 |
| 46. | 1. | 0. | 0.656001 | 2.993179 | 2.569227 |

**1a-1-3**


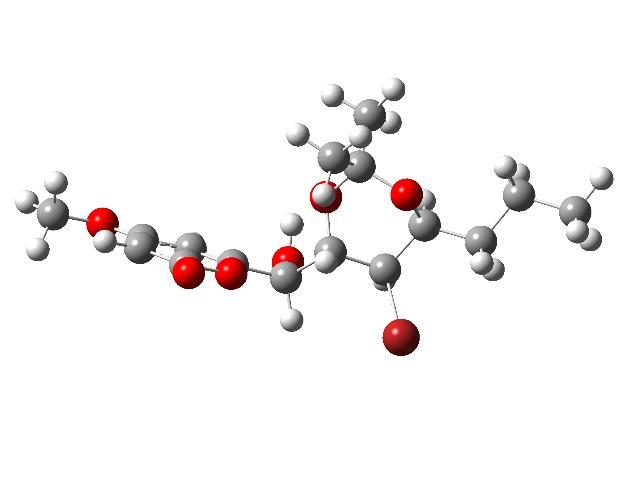
 E =-3649.230015 a.u

| **1a-1-3** | | Standard Orientation  (Ångstroms) | | | |
| --- | --- | --- | --- | --- | --- |
| Center number | Atom number | Type | X | Y | Z |
| 1. | 8. | 0. | 2.612709 | 0.941783 | 1.063775 |
| 2. | 6. | 0. | 2.200707 | -0.293703 | 0.721203 |
| 3. | 6. | 0. | 2.958267 | -1.126835 | -0.027516 |
| 4. | 6. | 0. | 4.241293 | -0.665100 | -0.477791 |
| 5. | 6. | 0. | 4.680628 | 0.589031 | -0.142558 |
| 6. | 6. | 0. | 3.873997 | 1.469863 | 0.661093 |
| 7. | 8. | 0. | 4.133687 | 2.590623 | 1.028501 |
| 8. | 6. | 0. | 0.815611 | -0.615104 | 1.246316 |
| 9. | 6. | 0. | -0.289088 | -0.119400 | 0.292215 |
| 10. | 6. | 0. | -1.683422 | -0.597789 | 0.735245 |
| 11. | 6. | 0. | -2.740698 | 0.416164 | 0.251100 |
| 12. | 6. | 0. | -4.124448 | -0.135451 | -0.072210 |
| 13. | 6. | 0. | -5.163717 | 0.961254 | -0.331812 |
| 14. | 6. | 0. | -6.543260 | 0.394436 | -0.678352 |
| 15. | 8. | 0. | 0.622583 | -0.110636 | 2.551622 |
| 16. | 8. | 0. | -2.247013 | 1.089151 | -0.909597 |
| 17. | 8. | 0. | 4.920201 | -1.561000 | -1.219769 |
| 18. | 8. | 0. | -0.273916 | 1.302413 | 0.334202 |
| 19. | 35. | 0. | -1.913527 | -2.452160 | 0.033029 |
| 20. | 6. | 0. | 6.212489 | -1.196440 | -1.704366 |
| 21. | 6. | 0. | -1.108636 | 1.915280 | -0.665176 |
| 22. | 6. | 0. | -1.502105 | 3.278308 | -0.100754 |
| 23. | 6. | 0. | -0.369527 | 2.029389 | -1.995443 |
| 24. | 1. | 0. | 2.620405 | -2.122434 | -0.284365 |
| 25. | 1. | 0. | 5.638115 | 0.985228 | -0.448930 |
| 26. | 1. | 0. | 0.720316 | -1.703553 | 1.308420 |
| 27. | 1. | 0. | -0.090082 | -0.487686 | -0.720809 |
| 28. | 1. | 0. | -1.730424 | -0.720077 | 1.817094 |
| 29. | 1. | 0. | -2.840985 | 1.138844 | 1.075737 |
| 30. | 1. | 0. | -4.444758 | -0.770579 | 0.763397 |
| 31. | 1. | 0. | -4.037264 | -0.792669 | -0.943920 |
| 32. | 1. | 0. | -5.243855 | 1.604895 | 0.555081 |
| 33. | 1. | 0. | -4.807840 | 1.600606 | -1.146912 |
| 34. | 1. | 0. | -6.935732 | -0.226528 | 0.134992 |
| 35. | 1. | 0. | -7.266548 | 1.194788 | -0.864016 |
| 36. | 1. | 0. | -6.499499 | -0.229726 | -1.577810 |
| 37. | 1. | 0. | 0.733380 | 0.850821 | 2.488955 |
| 38. | 1. | 0. | 6.154090 | -0.321994 | -2.361715 |
| 39. | 1. | 0. | 6.898098 | -0.985842 | -0.876359 |
| 40. | 1. | 0. | 6.571373 | -2.056837 | -2.268802 |
| 41. | 1. | 0. | -2.117635 | 3.816817 | -0.825279 |
| 42. | 1. | 0. | -2.065006 | 3.168952 | 0.829297 |
| 43. | 1. | 0. | -0.605849 | 3.866303 | 0.112423 |
| 44. | 1. | 0. | 0.511707 | 2.664508 | -1.879312 |
| 45. | 1. | 0. | -0.055506 | 1.044266 | -2.348871 |
| 46. | 1. | 0. | -1.027621 | 2.465407 | -2.751358 |

**1a-1-4**


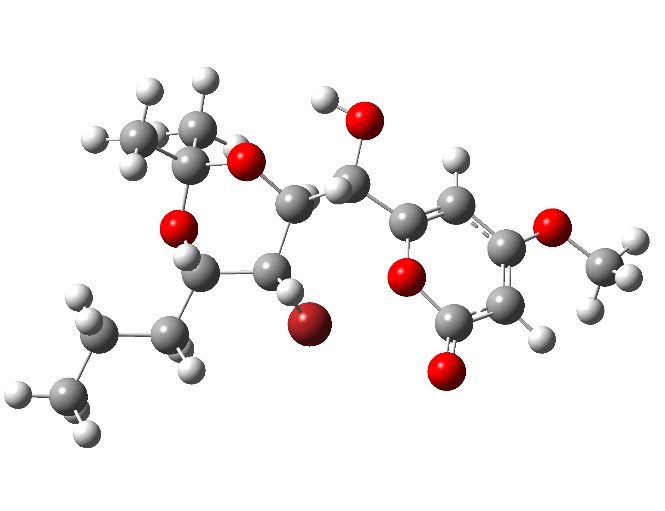
 E = -3649.2263331 a.u

| **1a-1-4** | | Standard Orientation  (Ångstroms) | | | |
| --- | --- | --- | --- | --- | --- |
| Center number | Atom number | Type | X | Y | Z |
| 1. | 8. | 0. | 2.601451 | 0.839395 | 1.137347 |
| 2. | 6. | 0. | 2.103846 | -0.357857 | 0.773916 |
| 3. | 6. | 0. | 2.834740 | -1.266848 | 0.089951 |
| 4. | 6. | 0. | 4.183221 | -0.928473 | -0.269596 |
| 5. | 6. | 0. | 4.709624 | 0.285968 | 0.086188 |
| 6. | 6. | 0. | 3.931226 | 1.246654 | 0.823361 |
| 7. | 8. | 0. | 4.264181 | 2.344286 | 1.201067 |
| 8. | 6. | 0. | 0.658380 | -0.541049 | 1.192406 |
| 9. | 6. | 0. | -0.315959 | 0.029022 | 0.142702 |
| 10. | 6. | 0. | -1.781788 | -0.294590 | 0.489913 |
| 11. | 6. | 0. | -2.688700 | 0.817995 | -0.076149 |
| 12. | 6. | 0. | -4.110721 | 0.435534 | -0.473231 |
| 13. | 6. | 0. | -4.970720 | -0.073036 | 0.690933 |
| 14. | 6. | 0. | -6.413726 | -0.360968 | 0.265779 |
| 15. | 8. | 0. | 0.411256 | 0.010865 | 2.469041 |
| 16. | 8. | 0. | -2.057707 | 1.391428 | -1.222637 |
| 17. | 8. | 0. | 4.828957 | -1.892063 | -0.954212 |
| 18. | 8. | 0. | -0.156210 | 1.442088 | 0.144951 |
| 19. | 35. | 0. | -2.157016 | -2.120797 | -0.223502 |
| 20. | 6. | 0. | 6.178743 | -1.650404 | -1.350674 |
| 21. | 6. | 0. | -0.853660 | 2.099405 | -0.928830 |
| 22. | 6. | 0. | -1.132475 | 3.517884 | -0.437195 |
| 23. | 6. | 0. | -0.023588 | 2.082923 | -2.209425 |
| 24. | 1. | 0. | 2.427082 | -2.231192 | -0.184341 |
| 25. | 1. | 0. | 5.718245 | 0.590128 | -0.154090 |
| 26. | 1. | 0. | 0.458010 | -1.614541 | 1.265271 |
| 27. | 1. | 0. | -0.078860 | -0.393787 | -0.840305 |
| 28. | 1. | 0. | -1.915182 | -0.398865 | 1.566144 |
| 29. | 1. | 0. | -2.746209 | 1.570500 | 0.725634 |
| 30. | 1. | 0. | -4.061075 | -0.320963 | -1.263560 |
| 31. | 1. | 0. | -4.566831 | 1.329987 | -0.913549 |
| 32. | 1. | 0. | -4.526891 | -0.988207 | 1.099437 |
| 33. | 1. | 0. | -4.969136 | 0.668137 | 1.501930 |
| 34. | 1. | 0. | -6.447513 | -1.121507 | -0.521900 |
| 35. | 1. | 0. | -7.009657 | -0.727128 | 1.107584 |
| 36. | 1. | 0. | -6.902072 | 0.540049 | -0.122048 |
| 37. | 1. | 0. | 0.627746 | 0.953866 | 2.402287 |
| 38. | 1. | 0. | 6.243712 | -0.783628 | -2.017498 |
| 39. | 1. | 0. | 6.822132 | -1.489622 | -0.478751 |
| 40. | 1. | 0. | 6.497263 | -2.547101 | -1.881848 |
| 41. | 1. | 0. | -1.635175 | 4.090202 | -1.220496 |
| 42. | 1. | 0. | -1.765461 | 3.506216 | 0.453516 |
| 43. | 1. | 0. | -0.193939 | 4.014889 | -0.178875 |
| 44. | 1. | 0. | 0.911240 | 2.627069 | -2.056042 |
| 45. | 1. | 0. | 0.205198 | 1.057534 | -2.509665 |
| 46. | 1. | 0. | -0.583166 | 2.554903 | -3.021017 |

**1a-1-5**


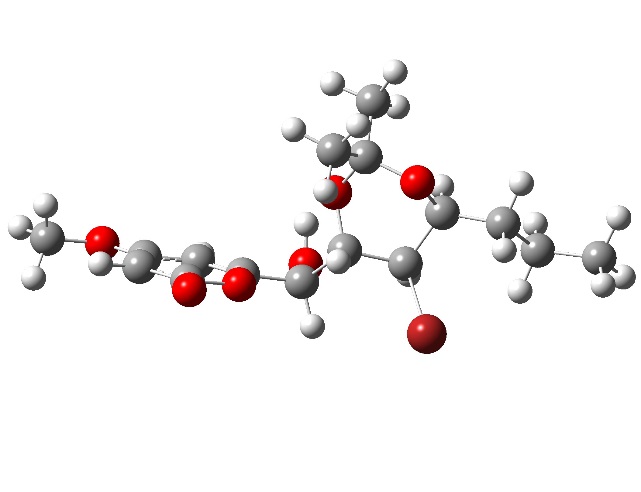
 E =-3649.2293557 a.u

| **1a-1-5** | | Standard Orientation  (Ångstroms) | | | |
| --- | --- | --- | --- | --- | --- |
| Center number | Atom number | Type | X | Y | Z |
| 1. | 8. | 0. | 1.940351 | -0.406275 | 1.414268 |
| 2. | 6. | 0. | 1.974743 | 0.773884 | 0.759604 |
| 3. | 6. | 0. | 3.001504 | 1.138792 | -0.036420 |
| 4. | 6. | 0. | 4.086270 | 0.213056 | -0.204255 |
| 5. | 6. | 0. | 4.060002 | -1.008119 | 0.417586 |
| 6. | 6. | 0. | 2.956653 | -1.392445 | 1.258090 |
| 7. | 8. | 0. | 2.798974 | -2.429080 | 1.857070 |
| 8. | 6. | 0. | 0.744231 | 1.600888 | 1.029409 |
| 9. | 6. | 0. | -0.468512 | 1.162926 | 0.178228 |
| 10. | 6. | 0. | -0.951010 | -0.289008 | 0.401295 |
| 11. | 6. | 0. | -2.487415 | -0.307690 | 0.389680 |
| 12. | 6. | 0. | -3.151058 | -1.651014 | 0.110458 |
| 13. | 6. | 0. | -4.667969 | -1.629137 | 0.333943 |
| 14. | 6. | 0. | -5.328868 | -2.972578 | 0.013662 |
| 15. | 8. | 0. | 1.039916 | 2.953843 | 0.750426 |
| 16. | 8. | 0. | -2.951051 | 0.635247 | -0.580101 |
| 17. | 8. | 0. | 5.073389 | 0.661155 | -1.004616 |
| 18. | 8. | 0. | -1.503841 | 2.074275 | 0.566244 |
| 19. | 35. | 0. | -0.144598 | -1.415316 | -1.028202 |
| 20. | 6. | 0. | 6.194938 | -0.190194 | -1.234167 |
| 21. | 6. | 0. | -2.700333 | 1.994001 | -0.231353 |
| 22. | 6. | 0. | -3.814682 | 2.558786 | 0.647131 |
| 23. | 6. | 0. | -2.549409 | 2.756829 | -1.544786 |
| 24. | 1. | 0. | 3.005719 | 2.101052 | -0.527469 |
| 25. | 1. | 0. | 4.845164 | -1.743547 | 0.317445 |
| 26. | 1. | 0. | 0.472404 | 1.458859 | 2.088220 |
| 27. | 1. | 0. | -0.215992 | 1.305017 | -0.877868 |
| 28. | 1. | 0. | -0.560945 | -0.724935 | 1.321358 |
| 29. | 1. | 0. | -2.785555 | 0.023889 | 1.397177 |
| 30. | 1. | 0. | -2.684669 | -2.405410 | 0.757196 |
| 31. | 1. | 0. | -2.923892 | -1.941778 | -0.920111 |
| 32. | 1. | 0. | -4.880117 | -1.355005 | 1.376713 |
| 33. | 1. | 0. | -5.104983 | -0.840495 | -0.288374 |
| 34. | 1. | 0. | -4.926042 | -3.775166 | 0.641749 |
| 35. | 1. | 0. | -6.410380 | -2.932796 | 0.177825 |
| 36. | 1. | 0. | -5.160952 | -3.256154 | -1.031217 |
| 37. | 1. | 0. | 0.182067 | 3.404699 | 0.738253 |
| 38. | 1. | 0. | 6.714080 | -0.418991 | -0.296845 |
| 39. | 1. | 0. | 5.888411 | -1.123866 | -1.718604 |
| 40. | 1. | 0. | 6.859475 | 0.365529 | -1.895580 |
| 41. | 1. | 0. | -4.757833 | 2.553559 | 0.095707 |
| 42. | 1. | 0. | -3.931861 | 1.960525 | 1.553910 |
| 43. | 1. | 0. | -3.582901 | 3.585478 | 0.942886 |
| 44. | 1. | 0. | -2.383750 | 3.819623 | -1.350039 |
| 45. | 1. | 0. | -1.714472 | 2.363949 | -2.129322 |
| 46. | 1. | 0. | -3.458474 | 2.644849 | -2.140908 |

4. For **1a-2**

Table S4.1. Gibbs free energies*^a^* and equilibrium populations*^b^* of low-energy conformers of **1a-2**.

| Conformers | In gas | |
| --- | --- | --- |
|  | G | *P* (%) |
| **1a-2-1** | -2288089.12627815 | 55.42 |
| **1a-2-2** | -2288088.5458314 | 20.79 |
| **1a-2-3** | -2288087.95408947 | 9.29 |
| **1a-2-4** | -2288088.0689238 | 7.65 |
| **1a-2-5** | -2288087.88945594 | 6.86 |
| *^a^*B3LYP/6-31G(d,p), in kcal/mol. *^b^*From G values at 298.15K. | | |

Table S4.2. Cartesian coordinates for the low-energy reoptimized MMFF conformers of **1a-2** at B3LYP/6-31G(d,p) level of theory in gas.

**1a-2-1**


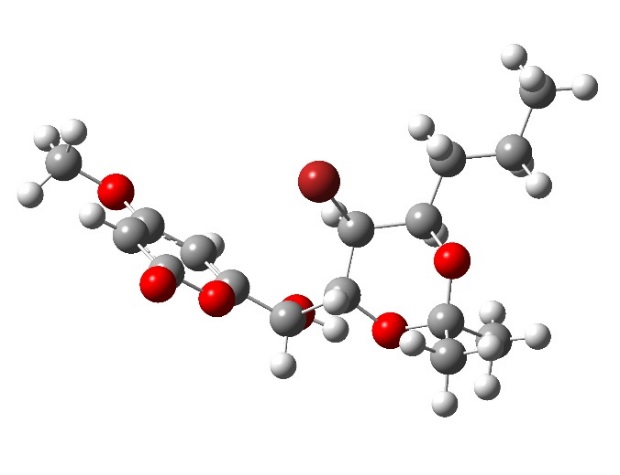
 E =-3649.2276865 a.u

| **1a-2-1** | | Standard Orientation  (Ångstroms) | | | |
| --- | --- | --- | --- | --- | --- |
| Center number | Atomic number | Atomic Type | X | Y | Z |
| 1. | 8. | 0. | -2.277144 | -1.304763 | 0.810705 |
| 2. | 6. | 0. | -1.947935 | -1.055474 | -0.473149 |
| 3. | 6. | 0. | -2.689204 | -0.271346 | -1.283838 |
| 4. | 6. | 0. | -3.866981 | 0.340320 | -0.734673 |
| 5. | 6. | 0. | -4.212670 | 0.128187 | 0.573923 |
| 6. | 6. | 0. | -3.414035 | -0.711495 | 1.429319 |
| 7. | 8. | 0. | -3.600270 | -0.979888 | 2.591843 |
| 8. | 6. | 0. | -0.675226 | -1.759043 | -0.859052 |
| 9. | 6. | 0. | 0.552667 | -1.226825 | -0.089083 |
| 10. | 6. | 0. | 0.862305 | 0.261987 | -0.366816 |
| 11. | 6. | 0. | 2.385712 | 0.449137 | -0.469749 |
| 12. | 6. | 0. | 2.923562 | 1.853247 | -0.219670 |
| 13. | 6. | 0. | 4.412915 | 1.992679 | -0.556394 |
| 14. | 6. | 0. | 4.952058 | 3.398134 | -0.276226 |
| 15. | 8. | 0. | -0.476843 | -1.620228 | -2.252722 |
| 16. | 8. | 0. | 3.024313 | -0.446535 | 0.442133 |
| 17. | 8. | 0. | -4.547925 | 1.106589 | -1.609593 |
| 18. | 8. | 0. | 1.632533 | -2.025100 | -0.584943 |
| 19. | 35. | 0. | 0.026717 | 1.342166 | 1.080869 |
| 20. | 6. | 0. | -5.727220 | 1.766279 | -1.151924 |
| 21. | 6. | 0. | 2.886305 | -1.821139 | 0.091505 |
| 22. | 6. | 0. | 2.940992 | -2.608979 | 1.397085 |
| 23. | 6. | 0. | 3.964302 | -2.256812 | -0.898944 |
| 24. | 1. | 0. | -2.403361 | -0.109108 | -2.312829 |
| 25. | 1. | 0. | -5.083133 | 0.568634 | 1.038347 |
| 26. | 1. | 0. | -0.779432 | -2.818754 | -0.577431 |
| 27. | 1. | 0. | 0.407823 | -1.383162 | 0.983736 |
| 28. | 1. | 0. | 0.371966 | 0.608180 | -1.277147 |
| 29. | 1. | 0. | 2.639426 | 0.162422 | -1.503098 |
| 30. | 1. | 0. | 2.745317 | 2.111035 | 0.829160 |
| 31. | 1. | 0. | 2.332898 | 2.559125 | -0.817912 |
| 32. | 1. | 0. | 4.976527 | 1.254785 | 0.024769 |
| 33. | 1. | 0. | 4.573509 | 1.742619 | -1.614417 |
| 34. | 1. | 0. | 4.421159 | 4.154480 | -0.865549 |
| 35. | 1. | 0. | 4.835717 | 3.662536 | 0.780607 |
| 36. | 1. | 0. | 6.016155 | 3.472081 | -0.522100 |
| 37. | 1. | 0. | 0.398349 | -1.997519 | -2.427070 |
| 38. | 1. | 0. | -5.498894 | 2.458013 | -0.333596 |
| 39. | 1. | 0. | -6.107814 | 2.323079 | -2.008116 |
| 40. | 1. | 0. | -6.479353 | 1.042614 | -0.818689 |
| 41. | 1. | 0. | 2.131771 | -2.307304 | 2.065835 |
| 42. | 1. | 0. | 2.858539 | -3.679834 | 1.195653 |
| 43. | 1. | 0. | 3.888250 | -2.414648 | 1.906479 |
| 44. | 1. | 0. | 4.950466 | -2.155556 | -0.439891 |
| 45. | 1. | 0. | 3.932925 | -1.642875 | -1.802729 |
| 46. | 1. | 0. | 3.813511 | -3.300397 | -1.187685 |

**1a-2-2**


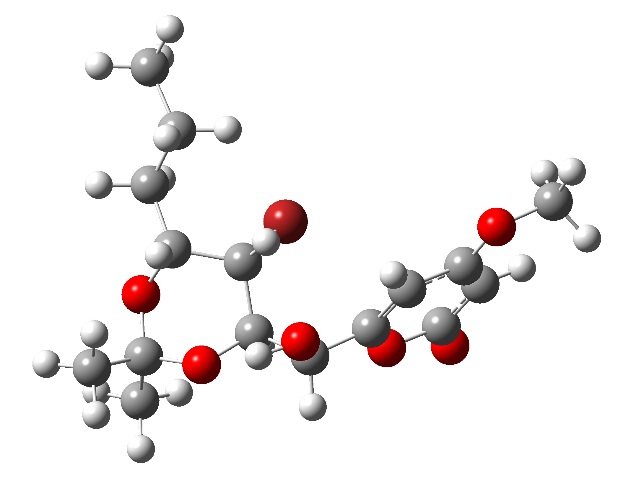
 E =-3649.2272526 a.u

| **1a-2-2** | | Standard Orientation  (Ångstroms) | | | |
| --- | --- | --- | --- | --- | --- |
| Center number | Atom number | Type | X | Y | Z |
| 1. | 8. | 0. | 2.053326 | -1.573728 | -0.655640 |
| 2. | 6. | 0. | 1.721190 | -1.155469 | 0.582770 |
| 3. | 6. | 0. | 2.488374 | -0.311812 | 1.304703 |
| 4. | 6. | 0. | 3.698822 | 0.175913 | 0.704988 |
| 5. | 6. | 0. | 4.049406 | -0.209241 | -0.562224 |
| 6. | 6. | 0. | 3.222434 | -1.110483 | -1.322836 |
| 7. | 8. | 0. | 3.408681 | -1.525982 | -2.441293 |
| 8. | 6. | 0. | 0.413690 | -1.750786 | 1.029985 |
| 9. | 6. | 0. | -0.781052 | -1.257830 | 0.185246 |
| 10. | 6. | 0. | -1.018608 | 0.266274 | 0.279305 |
| 11. | 6. | 0. | -2.533211 | 0.539377 | 0.324089 |
| 12. | 6. | 0. | -3.009572 | 1.916819 | -0.124357 |
| 13. | 6. | 0. | -2.434089 | 3.077421 | 0.697112 |
| 14. | 6. | 0. | -3.008246 | 4.431943 | 0.270720 |
| 15. | 8. | 0. | 0.208986 | -1.439137 | 2.394486 |
| 16. | 8. | 0. | -3.200437 | -0.432899 | -0.481855 |
| 17. | 8. | 0. | 4.404367 | 1.012717 | 1.491360 |
| 18. | 8. | 0. | -1.902563 | -1.937328 | 0.758450 |
| 19. | 35. | 0. | -0.108795 | 1.126170 | -1.269202 |
| 20. | 6. | 0. | 5.617318 | 1.558766 | 0.975869 |
| 21. | 6. | 0. | -3.137973 | -1.756475 | 0.041428 |
| 22. | 6. | 0. | -3.221152 | -2.700689 | -1.154274 |
| 23. | 6. | 0. | -4.247791 | -2.006172 | 1.060863 |
| 24. | 1. | 0. | 2.199508 | -0.013577 | 2.301917 |
| 25. | 1. | 0. | 4.944586 | 0.132856 | -1.061421 |
| 26. | 1. | 0. | 0.471446 | -2.839939 | 0.876612 |
| 27. | 1. | 0. | -0.636410 | -1.548193 | -0.859350 |
| 28. | 1. | 0. | -0.522943 | 0.690955 | 1.152195 |
| 29. | 1. | 0. | -2.817485 | 0.402709 | 1.379873 |
| 30. | 1. | 0. | -4.103812 | 1.913124 | -0.054714 |
| 31. | 1. | 0. | -2.755977 | 2.043153 | -1.181909 |
| 32. | 1. | 0. | -2.638164 | 2.913476 | 1.764348 |
| 33. | 1. | 0. | -1.344026 | 3.097063 | 0.583050 |
| 34. | 1. | 0. | -2.790352 | 4.637013 | -0.783004 |
| 35. | 1. | 0. | -4.096492 | 4.460350 | 0.395920 |
| 36. | 1. | 0. | -2.582972 | 5.247791 | 0.863534 |
| 37. | 1. | 0. | -0.686837 | -1.747606 | 2.597284 |
| 38. | 1. | 0. | 5.429761 | 2.153248 | 0.074879 |
| 39. | 1. | 0. | 6.013253 | 2.199977 | 1.763272 |
| 40. | 1. | 0. | 6.339637 | 0.767482 | 0.746544 |
| 41. | 1. | 0. | -2.389212 | -2.530545 | -1.841400 |
| 42. | 1. | 0. | -3.199926 | -3.739828 | -0.816858 |
| 43. | 1. | 0. | -4.150568 | -2.522739 | -1.701080 |
| 44. | 1. | 0. | -4.195144 | -1.281403 | 1.877404 |
| 45. | 1. | 0. | -4.154211 | -3.008991 | 1.485961 |
| 46. | 1. | 0. | -5.221968 | -1.916833 | 0.574655 |

**1a-2-3**


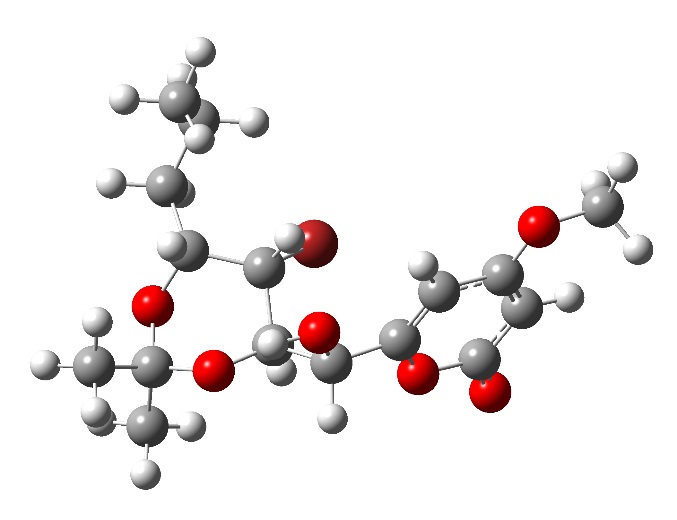
 E =-3649.2257739 a.u

| **1a-2-3** | | Standard Orientation  (Ångstroms) | | | |
| --- | --- | --- | --- | --- | --- |
| Center number | Atom number | Type | X | Y | Z |
| 1. | 8. | 0. | 2.007124 | -1.698527 | -0.329391 |
| 2. | 6. | 0. | 1.647367 | -1.028136 | 0.784104 |
| 3. | 6. | 0. | 2.419653 | -0.078586 | 1.352929 |
| 4. | 6. | 0. | 3.668752 | 0.240761 | 0.720037 |
| 5. | 6. | 0. | 4.047564 | -0.401118 | -0.429488 |
| 6. | 6. | 0. | 3.213796 | -1.411646 | -1.028275 |
| 7. | 8. | 0. | 3.422571 | -2.048039 | -2.033055 |
| 8. | 6. | 0. | 0.303334 | -1.479528 | 1.288334 |
| 9. | 6. | 0. | -0.842496 | -1.133197 | 0.312759 |
| 10. | 6. | 0. | -1.015118 | 0.385290 | 0.076653 |
| 11. | 6. | 0. | -2.515813 | 0.719248 | 0.021629 |
| 12. | 6. | 0. | -2.927371 | 1.990027 | -0.717788 |
| 13. | 6. | 0. | -2.305302 | 3.292345 | -0.188515 |
| 14. | 6. | 0. | -2.678069 | 3.646801 | 1.256000 |
| 15. | 8. | 0. | 0.064262 | -0.887732 | 2.550657 |
| 16. | 8. | 0. | -3.206063 | -0.370818 | -0.592783 |
| 17. | 8. | 0. | 4.378056 | 1.195169 | 1.354177 |
| 18. | 8. | 0. | -2.008372 | -1.638077 | 0.971165 |
| 19. | 35. | 0. | -0.039014 | 0.858913 | -1.592733 |
| 20. | 6. | 0. | 5.630129 | 1.588370 | 0.794636 |
| 21. | 6. | 0. | -3.216313 | -1.560043 | 0.190904 |
| 22. | 6. | 0. | -3.311663 | -2.725243 | -0.789427 |
| 23. | 6. | 0. | -4.360982 | -1.552560 | 1.202320 |
| 24. | 1. | 0. | 2.106628 | 0.423371 | 2.256767 |
| 25. | 1. | 0. | 4.971610 | -0.194073 | -0.949786 |
| 26. | 1. | 0. | 0.324324 | -2.578185 | 1.363369 |
| 27. | 1. | 0. | -0.677961 | -1.641064 | -0.641976 |
| 28. | 1. | 0. | -0.516965 | 0.965946 | 0.853175 |
| 29. | 1. | 0. | -2.828619 | 0.802998 | 1.073298 |
| 30. | 1. | 0. | -4.021035 | 2.046934 | -0.661338 |
| 31. | 1. | 0. | -2.668316 | 1.861316 | -1.772835 |
| 32. | 1. | 0. | -1.215560 | 3.243527 | -0.296231 |
| 33. | 1. | 0. | -2.631947 | 4.104803 | -0.848610 |
| 34. | 1. | 0. | -3.765495 | 3.693612 | 1.385745 |
| 35. | 1. | 0. | -2.290976 | 2.917451 | 1.976111 |
| 36. | 1. | 0. | -2.268996 | 4.622741 | 1.535672 |
| 37. | 1. | 0. | -0.849912 | -1.118844 | 2.773905 |
| 38. | 1. | 0. | 5.499490 | 2.000474 | -0.212087 |
| 39. | 1. | 0. | 6.023884 | 2.358084 | 1.458279 |
| 40. | 1. | 0. | 6.326921 | 0.743718 | 0.755080 |
| 41. | 1. | 0. | -2.454293 | -2.732578 | -1.466168 |
| 42. | 1. | 0. | -3.348281 | -3.673255 | -0.247208 |
| 43. | 1. | 0. | -4.216681 | -2.625636 | -1.393973 |
| 44. | 1. | 0. | -4.324591 | -2.451394 | 1.823628 |
| 45. | 1. | 0. | -5.317879 | -1.524236 | 0.675967 |
| 46. | 1. | 0. | -4.295304 | -0.680181 | 1.857843 |

**1a-2-4**


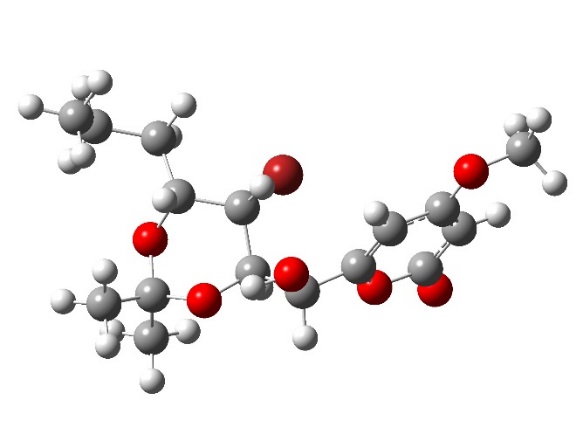
 E =-3649.2265993 a.u

| **1a-2-4** | | Standard Orientation  (Ångstroms) | | | |
| --- | --- | --- | --- | --- | --- |
| Center number | Atom number | Type | X | Y | Z |
| 1. | 8. | 0. | 2.245231 | -1.467918 | -0.527828 |
| 2. | 6. | 0. | 1.868049 | -0.981758 | 0.672387 |
| 3. | 6. | 0. | 2.606268 | -0.094176 | 1.371404 |
| 4. | 6. | 0. | 3.835025 | 0.365944 | 0.787185 |
| 5. | 6. | 0. | 4.231000 | -0.088050 | -0.443304 |
| 6. | 6. | 0. | 3.434979 | -1.036271 | -1.179400 |
| 7. | 8. | 0. | 3.662075 | -1.513713 | -2.265104 |
| 8. | 6. | 0. | 0.546979 | -1.556872 | 1.105720 |
| 9. | 6. | 0. | -0.616420 | -1.122715 | 0.187912 |
| 10. | 6. | 0. | -0.863952 | 0.403159 | 0.182131 |
| 11. | 6. | 0. | -2.379208 | 0.667634 | 0.173002 |
| 12. | 6. | 0. | -2.828429 | 2.025619 | -0.357470 |
| 13. | 6. | 0. | -4.327314 | 2.306770 | -0.169235 |
| 14. | 6. | 0. | -4.761873 | 2.518868 | 1.285353 |
| 15. | 8. | 0. | 0.289425 | -1.164708 | 2.440185 |
| 16. | 8. | 0. | -3.023069 | -0.347362 | -0.599906 |
| 17. | 8. | 0. | 4.509370 | 1.250180 | 1.548642 |
| 18. | 8. | 0. | -1.755930 | -1.776396 | 0.756464 |
| 19. | 35. | 0. | 0.091098 | 1.170210 | -1.387862 |
| 20. | 6. | 0. | 5.736937 | 1.774036 | 1.044821 |
| 21. | 6. | 0. | -2.966041 | -1.644304 | -0.011625 |
| 22. | 6. | 0. | -2.998443 | -2.644801 | -1.163343 |
| 23. | 6. | 0. | -4.108200 | -1.858472 | 0.979562 |
| 24. | 1. | 0. | 2.281517 | 0.258939 | 2.339218 |
| 25. | 1. | 0. | 5.141302 | 0.231322 | -0.930026 |
| 26. | 1. | 0. | 0.614406 | -2.652970 | 1.020879 |
| 27. | 1. | 0. | -0.429544 | -1.473300 | -0.831109 |
| 28. | 1. | 0. | -0.397340 | 0.885906 | 1.041285 |
| 29. | 1. | 0. | -2.685600 | 0.576154 | 1.226060 |
| 30. | 1. | 0. | -2.571179 | 2.069689 | -1.419618 |
| 31. | 1. | 0. | -2.233178 | 2.802410 | 0.139934 |
| 32. | 1. | 0. | -4.577438 | 3.203898 | -0.748103 |
| 33. | 1. | 0. | -4.898290 | 1.484332 | -0.614096 |
| 34. | 1. | 0. | -4.603339 | 1.625244 | 1.899065 |
| 35. | 1. | 0. | -4.205795 | 3.341244 | 1.750282 |
| 36. | 1. | 0. | -5.827013 | 2.764506 | 1.344163 |
| 37. | 1. | 0. | -0.610396 | -1.470148 | 2.629202 |
| 38. | 1. | 0. | 5.577791 | 2.316355 | 0.106242 |
| 39. | 1. | 0. | 6.103043 | 2.460511 | 1.808135 |
| 40. | 1. | 0. | 6.469935 | 0.975558 | 0.884654 |
| 41. | 1. | 0. | -2.146017 | -2.496505 | -1.830003 |
| 42. | 1. | 0. | -3.910973 | -2.504343 | -1.748132 |
| 43. | 1. | 0. | -2.977141 | -3.666609 | -0.776629 |
| 44. | 1. | 0. | -4.087998 | -1.098109 | 1.764485 |
| 45. | 1. | 0. | -4.022044 | -2.840876 | 1.451334 |
| 46. | 1. | 0. | -5.066090 | -1.798902 | 0.457727 |

**1a-2-5**


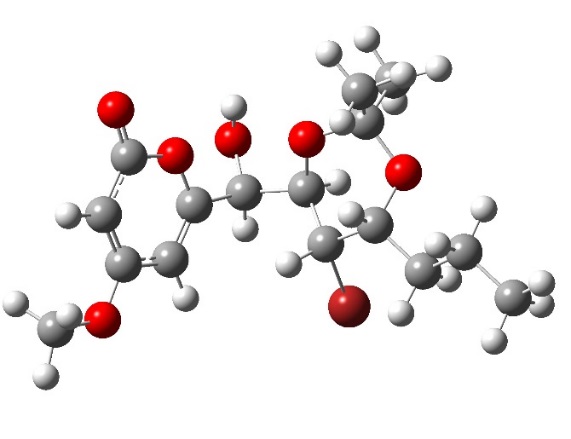
 E =-3649.2256650 a.u

| **1a-2-5** | | Standard Orientation  (Ångstroms) | | | |
| --- | --- | --- | --- | --- | --- |
| Center number | Atom number | Type | X | Y | Z |
| 1. | 8. | 0. | -2.474420 | 1.432028 | -0.103808 |
| 2. | 6. | 0. | -2.049299 | 0.325696 | -0.743635 |
| 3. | 6. | 0. | -2.587412 | -0.894285 | -0.510767 |
| 4. | 6. | 0. | -3.641898 | -1.004243 | 0.459139 |
| 5. | 6. | 0. | -4.089320 | 0.108727 | 1.120946 |
| 6. | 6. | 0. | -3.517877 | 1.406004 | 0.865720 |
| 7. | 8. | 0. | -3.813721 | 2.461374 | 1.372403 |
| 8. | 6. | 0. | -0.929144 | 0.608714 | -1.726272 |
| 9. | 6. | 0. | 0.468668 | 0.682341 | -1.076528 |
| 10. | 6. | 0. | 0.905178 | -0.550325 | -0.273136 |
| 11. | 6. | 0. | 2.010638 | -0.126679 | 0.722501 |
| 12. | 6. | 0. | 3.088366 | -1.159185 | 1.033597 |
| 13. | 6. | 0. | 4.013832 | -0.734299 | 2.179510 |
| 14. | 6. | 0. | 5.115104 | -1.761111 | 2.457990 |
| 15. | 8. | 0. | -1.176585 | 1.791475 | -2.455361 |
| 16. | 8. | 0. | 2.654840 | 1.053085 | 0.237626 |
| 17. | 8. | 0. | -4.109249 | -2.256489 | 0.625348 |
| 18. | 8. | 0. | 0.478905 | 1.801659 | -0.195781 |
| 19. | 35. | 0. | 1.434176 | -1.955405 | -1.590494 |
| 20. | 6. | 0. | -5.167613 | -2.463715 | 1.560612 |
| 21. | 6. | 0. | 1.809292 | 2.203598 | 0.182223 |
| 22. | 6. | 0. | 2.420479 | 3.129102 | -0.865905 |
| 23. | 6. | 0. | 1.670544 | 2.877954 | 1.544752 |
| 24. | 1. | 0. | -2.243000 | -1.772793 | -1.041218 |
| 25. | 1. | 0. | -4.881942 | 0.085426 | 1.854999 |
| 26. | 1. | 0. | -0.901980 | -0.213199 | -2.447135 |
| 27. | 1. | 0. | 1.177496 | 0.833144 | -1.898542 |
| 28. | 1. | 0. | 0.074172 | -1.010822 | 0.260825 |
| 29. | 1. | 0. | 1.477068 | 0.108033 | 1.656334 |
| 30. | 1. | 0. | 3.668378 | -1.337366 | 0.121698 |
| 31. | 1. | 0. | 2.595582 | -2.108993 | 1.276559 |
| 32. | 1. | 0. | 4.460518 | 0.234690 | 1.932520 |
| 33. | 1. | 0. | 3.418965 | -0.578413 | 3.090099 |
| 34. | 1. | 0. | 5.747651 | -1.911548 | 1.576133 |
| 35. | 1. | 0. | 5.761940 | -1.437091 | 3.279431 |
| 36. | 1. | 0. | 4.693218 | -2.735083 | 2.731198 |
| 37. | 1. | 0. | -1.160611 | 2.512145 | -1.806853 |
| 38. | 1. | 0. | -6.060809 | -1.897165 | 1.275632 |
| 39. | 1. | 0. | -5.384734 | -3.531257 | 1.531547 |
| 40. | 1. | 0. | -4.860452 | -2.176717 | 2.572323 |
| 41. | 1. | 0. | 2.450083 | 2.643040 | -1.843825 |
| 42. | 1. | 0. | 1.832977 | 4.047178 | -0.943083 |
| 43. | 1. | 0. | 3.444599 | 3.385803 | -0.583308 |
| 44. | 1. | 0. | 0.978323 | 3.720681 | 1.475175 |
| 45. | 1. | 0. | 2.644321 | 3.242918 | 1.880122 |
| 46. | 1. | 0. | 1.276675 | 2.181726 | 2.288754 |
