# Supplementary material for: Co-cultivation With 5-Azacytidine Induced New Metabolites From the Zoanthid-Derived Fungus Cochliobolus lunatus
Source: Front Chem. 2019 Nov 8;7:763. doi: 10.3389/fchem.2019.00763 (PMC6857680; doi:10.3389/fchem.2019.00763)
Supplement: Supplementary file 4 [file Table_4.DOCX]

**Supporting information 4.** The ECD calculation results of **1F-1**–**1F-4**

1. For **1F-1**

Table S1.1. Gibbs free energies*^a^* and equilibrium populations*^b^* low-energy conformers of **1F-1**.

| Conformers | ∆*G* | *P* (%) |
| --- | --- | --- |
| **1F-1-1** | 0 | 50.54 |
| **1F-1-2** | 0.0001236 | 44.33 |
| **1F-1-3** | 0.0027134 | 2.85 |
| **1F-1-4** | 0.0035226 | 1.21 |
| **1F-1-5** | 0.0036288 | 1.08 |

*^a^* B3LYP/6-311+G (d), in kcal/mol. *^b^*From ∆*G* values at 298.15K

Table S1.2. Cartesian coordinates for the low-energy reoptimized MMFF conformers of **1F-1** at B3LYP/6-311++G (2d, p) level of theory.

**1F-1-1**


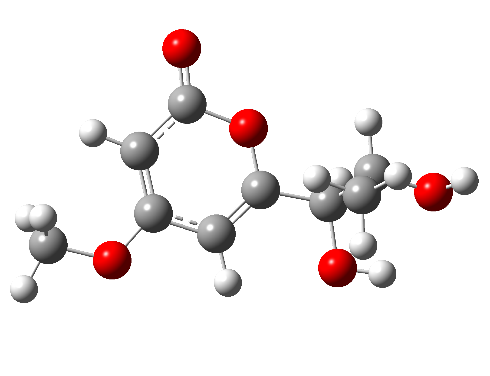
 E = -726.5173073 a.u

| **1F-1-1** | | Standard Orientation  (Ångstroms) | | | |
| --- | --- | --- | --- | --- | --- |
| No. | Atom | Type | X | Y | Z |
| 1 | 8 | 0 | 0.074481 | 1.279794 | -0.39724 |
| 2 | 6 | 0 | 0.335929 | -0.042574 | -0.441734 |
| 3 | 6 | 0 | -0.613181 | -0.9803 | -0.258339 |
| 4 | 6 | 0 | -1.957631 | -0.544379 | -0.01679 |
| 5 | 6 | 0 | -2.254533 | 0.790831 | 0.032581 |
| 6 | 6 | 0 | -1.237367 | 1.789865 | -0.150567 |
| 7 | 8 | 0 | -1.352355 | 2.984569 | -0.120874 |
| 8 | 6 | 0 | 1.783121 | -0.315854 | -0.732734 |
| 9 | 6 | 0 | 2.737635 | 0.317759 | 0.301513 |
| 10 | 6 | 0 | 2.500811 | -0.149811 | 1.732265 |
| 11 | 8 | 0 | 1.980299 | -1.713824 | -0.801088 |
| 12 | 8 | 0 | -2.840872 | -1.544732 | 0.145672 |
| 13 | 8 | 0 | 4.030957 | -0.089246 | -0.174999 |
| 14 | 6 | 0 | -4.208378 | -1.215012 | 0.382329 |
| 15 | 1 | 0 | -0.365879 | -2.029887 | -0.304905 |
| 16 | 1 | 0 | -3.248544 | 1.173968 | 0.210351 |
| 17 | 1 | 0 | 2.020826 | 0.150564 | -1.701035 |
| 18 | 1 | 0 | 2.639927 | 1.406998 | 0.236545 |
| 19 | 1 | 0 | 3.270097 | 0.253567 | 2.399071 |
| 20 | 1 | 0 | 1.534595 | 0.195033 | 2.107754 |
| 21 | 1 | 0 | 2.528012 | -1.239282 | 1.790687 |
| 22 | 1 | 0 | 2.937702 | -1.83967 | -0.848824 |
| 23 | 1 | 0 | 4.695951 | 0.149142 | 0.479322 |
| 24 | 1 | 0 | -4.726878 | -2.166171 | 0.477306 |
| 25 | 1 | 0 | -4.31889 | -0.640517 | 1.306214 |
| 26 | 1 | 0 | -4.625435 | -0.649381 | -0.45539 |

**1F-1-2**

**
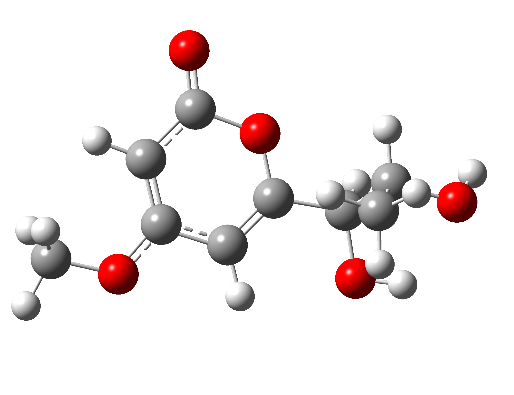
** E **= -**726.5171837 a.u

| **1F-1-2** | | Standard Orientation  (Ångstroms) | | | |
| --- | --- | --- | --- | --- | --- |
| No. | Atom | Type | X | Y | Z |
| 1 | 8 | 0 | 0.062753 | 1.302815 | -0.381947 |
| 2 | 6 | 0 | 0.337185 | -0.016478 | -0.448931 |
| 3 | 6 | 0 | -0.601992 | -0.967078 | -0.281947 |
| 4 | 6 | 0 | -1.949935 | -0.548748 | -0.028289 |
| 5 | 6 | 0 | -2.259598 | 0.782655 | 0.045155 |
| 6 | 6 | 0 | -1.253618 | 1.795161 | -0.12489 |
| 7 | 8 | 0 | -1.381417 | 2.988114 | -0.078324 |
| 8 | 6 | 0 | 1.789358 | -0.262427 | -0.735884 |
| 9 | 6 | 0 | 2.735877 | 0.296377 | 0.36044 |
| 10 | 6 | 0 | 2.475601 | -0.267754 | 1.745811 |
| 11 | 8 | 0 | 2.005211 | -1.646725 | -0.904444 |
| 12 | 8 | 0 | -2.822723 | -1.560148 | 0.118874 |
| 13 | 8 | 0 | 4.070797 | -0.096621 | -0.006028 |
| 14 | 6 | 0 | -4.192722 | -1.248392 | 0.366946 |
| 15 | 1 | 0 | -0.344168 | -2.01311 | -0.348986 |
| 16 | 1 | 0 | -3.257089 | 1.15214 | 0.232022 |
| 17 | 1 | 0 | 2.026449 | 0.277745 | -1.66907 |
| 18 | 1 | 0 | 2.651068 | 1.387704 | 0.373754 |
| 19 | 1 | 0 | 3.244278 | 0.085656 | 2.434781 |
| 20 | 1 | 0 | 1.500164 | 0.048389 | 2.120714 |
| 21 | 1 | 0 | 2.50088 | -1.358841 | 1.729574 |
| 22 | 1 | 0 | 2.962228 | -1.772143 | -0.827221 |
| 23 | 1 | 0 | 4.382894 | 0.483461 | -0.710516 |
| 24 | 1 | 0 | -4.700941 | -2.206359 | 0.447729 |
| 25 | 1 | 0 | -4.304726 | -0.691105 | 1.301061 |
| 26 | 1 | 0 | -4.618933 | -0.672922 | -0.45935 |

**1F-1-3**

**
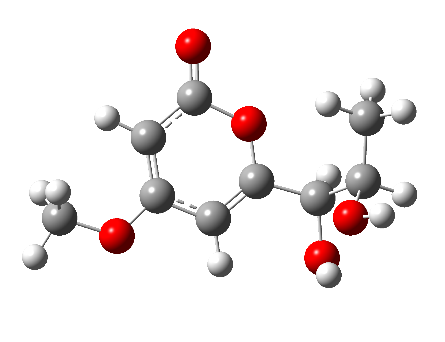
** E **=** -726.5145939 a.u

| **1F-1-3** | | Standard Orientation  (Ångstroms) | | | |
| --- | --- | --- | --- | --- | --- |
| No. | Atom | Type | X | Y | Z |
| 1 | 8 | 0 | 0.230508 | 1.184186 | -0.475416 |
| 2 | 6 | 0 | 0.410854 | -0.151508 | -0.489841 |
| 3 | 6 | 0 | -0.597543 | -1.016283 | -0.264694 |
| 4 | 6 | 0 | -1.899861 | -0.487963 | 0.014456 |
| 5 | 6 | 0 | -2.107755 | 0.864414 | 0.045581 |
| 6 | 6 | 0 | -1.033947 | 1.787641 | -0.195693 |
| 7 | 8 | 0 | -1.066256 | 2.988342 | -0.191168 |
| 8 | 6 | 0 | 1.834138 | -0.543627 | -0.835819 |
| 9 | 6 | 0 | 2.892284 | -0.148903 | 0.217377 |
| 10 | 6 | 0 | 2.97416 | 1.316685 | 0.618554 |
| 11 | 8 | 0 | 1.934673 | -1.935612 | -1.065714 |
| 12 | 8 | 0 | -2.840652 | -1.425194 | 0.227347 |
| 13 | 8 | 0 | 2.601579 | -0.998763 | 1.340309 |
| 14 | 6 | 0 | -4.173197 | -1.001141 | 0.507454 |
| 15 | 1 | 0 | -0.437229 | -2.082004 | -0.316619 |
| 16 | 1 | 0 | -3.067267 | 1.316092 | 0.25009 |
| 17 | 1 | 0 | 2.087566 | -0.042009 | -1.775675 |
| 18 | 1 | 0 | 3.850103 | -0.459422 | -0.223036 |
| 19 | 1 | 0 | 3.832182 | 1.46932 | 1.281654 |
| 20 | 1 | 0 | 3.11209 | 1.960615 | -0.253599 |
| 21 | 1 | 0 | 2.074523 | 1.642077 | 1.138086 |
| 22 | 1 | 0 | 2.090388 | -2.334396 | -0.196751 |
| 23 | 1 | 0 | 3.328025 | -0.947919 | 1.970259 |
| 24 | 1 | 0 | -4.74984 | -1.913692 | 0.639556 |
| 25 | 1 | 0 | -4.210524 | -0.405452 | 1.423788 |
| 26 | 1 | 0 | -4.583627 | -0.422776 | -0.324875 |

**1F-1-4**

**
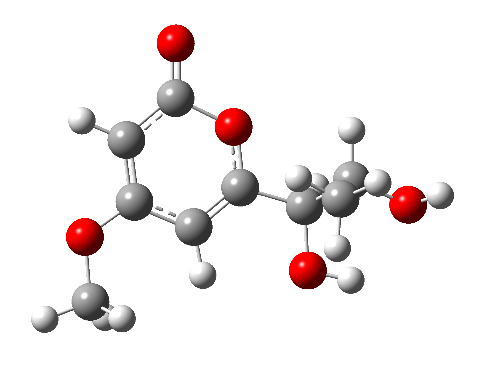
** E **=** -726.5137847 a.u

| **1F-1-4** | | Standard Orientation  (Ångstroms) | | | |
| --- | --- | --- | --- | --- | --- |
| No. | Atom | Type | X | Y | Z |
| 1 | 8 | 0 | 0.062458 | 1.408649 | -0.381983 |
| 2 | 6 | 0 | 0.206799 | 0.078084 | -0.428657 |
| 3 | 6 | 0 | -0.813577 | -0.784283 | -0.221054 |
| 4 | 6 | 0 | -2.108386 | -0.234618 | 0.054702 |
| 5 | 6 | 0 | -2.284427 | 1.122638 | 0.109398 |
| 6 | 6 | 0 | -1.20727 | 2.038694 | -0.100266 |
| 7 | 8 | 0 | -1.205814 | 3.237822 | -0.074689 |
| 8 | 6 | 0 | 1.617094 | -0.328183 | -0.749516 |
| 9 | 6 | 0 | 2.649735 | 0.24017 | 0.247009 |
| 10 | 6 | 0 | 2.404718 | -0.167232 | 1.694666 |
| 11 | 8 | 0 | 1.690649 | -1.74014 | -0.791261 |
| 12 | 8 | 0 | -3.193068 | -1.004328 | 0.271008 |
| 13 | 8 | 0 | 3.888 | -0.296906 | -0.245855 |
| 14 | 6 | 0 | -3.07263 | -2.425452 | 0.220548 |
| 15 | 1 | 0 | -0.611459 | -1.841578 | -0.279992 |
| 16 | 1 | 0 | -3.258725 | 1.543625 | 0.317235 |
| 17 | 1 | 0 | 1.869395 | 0.094218 | -1.733971 |
| 18 | 1 | 0 | 2.649918 | 1.331578 | 0.154297 |
| 19 | 1 | 0 | 3.223164 | 0.180062 | 2.333741 |
| 20 | 1 | 0 | 1.483761 | 0.275542 | 2.081047 |
| 21 | 1 | 0 | 2.334289 | -1.252851 | 1.782651 |
| 22 | 1 | 0 | 2.632416 | -1.949957 | -0.855354 |
| 23 | 1 | 0 | 4.590001 | -0.096051 | 0.381943 |
| 24 | 1 | 0 | -4.070896 | -2.805877 | 0.425079 |
| 25 | 1 | 0 | -2.751029 | -2.763826 | -0.767955 |
| 26 | 1 | 0 | -2.38098 | -2.794559 | 0.982538 |

**1F-1-5**

**
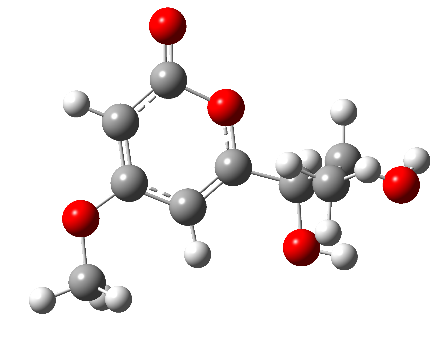
** E **=** -726.5136785 a.u

| **1F-1-5** | | Standard Orientation  (Ångstroms) | | | |
| --- | --- | --- | --- | --- | --- |
| No. | Atom | Type | X | Y | Z |
| 1 | 8 | 0 | 0.045541 | 1.428407 | -0.372029 |
| 2 | 6 | 0 | 0.207318 | 0.099923 | -0.432987 |
| 3 | 6 | 0 | -0.800806 | -0.77867 | -0.234716 |
| 4 | 6 | 0 | -2.102182 | -0.249424 | 0.050717 |
| 5 | 6 | 0 | -2.296056 | 1.104853 | 0.121261 |
| 6 | 6 | 0 | -1.232519 | 2.038106 | -0.081891 |
| 7 | 8 | 0 | -1.247722 | 3.236837 | -0.046619 |
| 8 | 6 | 0 | 1.625721 | -0.276199 | -0.751369 |
| 9 | 6 | 0 | 2.646561 | 0.232183 | 0.301793 |
| 10 | 6 | 0 | 2.375776 | -0.261854 | 1.711652 |
| 11 | 8 | 0 | 1.72094 | -1.679284 | -0.881986 |
| 12 | 8 | 0 | -3.176074 | -1.035727 | 0.259506 |
| 13 | 8 | 0 | 3.93146 | -0.288008 | -0.084219 |
| 14 | 6 | 0 | -3.036319 | -2.454682 | 0.195539 |
| 15 | 1 | 0 | -0.58454 | -1.832351 | -0.307818 |
| 16 | 1 | 0 | -3.275602 | 1.510155 | 0.335481 |
| 17 | 1 | 0 | 1.879912 | 0.212988 | -1.707438 |
| 18 | 1 | 0 | 2.656277 | 1.326367 | 0.279665 |
| 19 | 1 | 0 | 3.188685 | 0.048063 | 2.369991 |
| 20 | 1 | 0 | 1.440443 | 0.148489 | 2.097527 |
| 21 | 1 | 0 | 2.310158 | -1.351244 | 1.733625 |
| 22 | 1 | 0 | 2.666239 | -1.882045 | -0.8256 |
| 23 | 1 | 0 | 4.279483 | 0.247297 | -0.807001 |
| 24 | 1 | 0 | -4.029128 | -2.850548 | 0.397138 |
| 25 | 1 | 0 | -2.710943 | -2.779071 | -0.796399 |
| 26 | 1 | 0 | -2.339103 | -2.821321 | 0.9536 |

2. For **1F-2**

Table S2.1. Gibbs free energies*^a^* and equilibrium populations*^b^* low-energy conformers of **1F-2**.

| Conformers | ∆*G* | *P* (%) |
| --- | --- | --- |
| **1F-2-1** | 0.0028186 | 1.39 |
| **1F-2-2** | 0.0003101 | 19.93 |
| **1F-2-3** | 0.0009411 | 10.21 |
| **1F-2-4** | 0.0002405 | 21.46 |
| **1F-2-5** | 0.0018915 | 3.73 |
| **1F-2-6** | 0 | 27.69 |
| **1F-2-7** | 0.0025575 | 1.84 |
| **1F-2-8** | 0.001731 | 4.42 |
| **1F-2-9** | 0.0028225 | 1.39 |
| **1F-2-10** | 0.0011769 | 7.95 |

*^a^* B3LYP/6-311+G (d), in kcal/mol. *^b^*From ∆*G* values at 298.15K

Table S2.2. Cartesian coordinates for the low-energy reoptimized MMFF conformers of **1F-2** at B3LYP/6-311++G (2d, p) level of theory.

**1F-2-1**


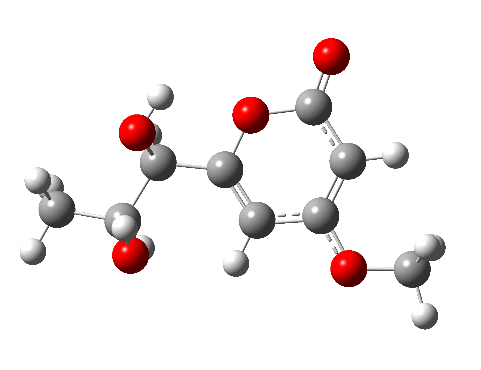
 E = -726.5155629 a.u

| **1F-2-1** | | Standard Orientation  (Ångstroms) | | | |
| --- | --- | --- | --- | --- | --- |
| No. | Atom | Type | X | Y | Z |
| 1 | 8 | 0 | 0.233455 | 1.566495 | -0.1641 |
| 2 | 6 | 0 | -0.348305 | 0.342409 | -0.195121 |
| 3 | 6 | 0 | 0.372971 | -0.792656 | -0.139203 |
| 4 | 6 | 0 | 1.804075 | -0.691831 | -0.065704 |
| 5 | 6 | 0 | 2.412499 | 0.532534 | -0.033179 |
| 6 | 6 | 0 | 1.645532 | 1.74829 | -0.061698 |
| 7 | 8 | 0 | 2.04101 | 2.880546 | -0.014537 |
| 8 | 6 | 0 | -1.848634 | 0.497714 | -0.27444 |
| 9 | 6 | 0 | -2.643465 | -0.810122 | -0.364525 |
| 10 | 6 | 0 | -4.122314 | -0.54867 | -0.648072 |
| 11 | 8 | 0 | -2.319849 | 1.170681 | 0.898417 |
| 12 | 8 | 0 | 2.433819 | -1.877489 | -0.029065 |
| 13 | 8 | 0 | -2.46901 | -1.578081 | 0.820887 |
| 14 | 6 | 0 | 3.858458 | -1.891447 | 0.057457 |
| 15 | 1 | 0 | -0.10411 | -1.760338 | -0.103217 |
| 16 | 1 | 0 | 3.482924 | 0.664207 | 0.026267 |
| 17 | 1 | 0 | -2.06165 | 1.110358 | -1.162489 |
| 18 | 1 | 0 | -2.224205 | -1.41673 | -1.172352 |
| 19 | 1 | 0 | -4.663016 | -1.496573 | -0.650762 |
| 20 | 1 | 0 | -4.265221 | -0.065654 | -1.619822 |
| 21 | 1 | 0 | -4.553584 | 0.096006 | 0.120493 |
| 22 | 1 | 0 | -1.798964 | 1.974716 | 1.013384 |
| 23 | 1 | 0 | -2.599366 | -0.973946 | 1.564658 |
| 24 | 1 | 0 | 4.139874 | -2.941505 | 0.078975 |
| 25 | 1 | 0 | 4.308027 | -1.40631 | -0.813233 |
| 26 | 1 | 0 | 4.198993 | -1.398771 | 0.972184 |

**1F-2-2**


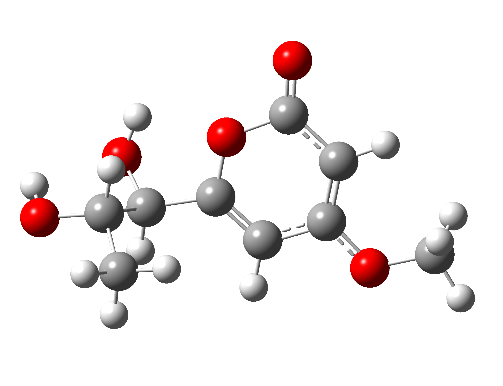
E = -726.5180714 a.u

| **1F-2-2** | | Standard Orientation  (Ångstroms) | | | |
| --- | --- | --- | --- | --- | --- |
| No. | Atom | Type | X | Y | Z |
| 1 | 8 | 0 | -0.117079 | 1.23658 | 0.033285 |
| 2 | 6 | 0 | -0.345572 | -0.018282 | -0.411916 |
| 3 | 6 | 0 | 0.644375 | -0.923526 | -0.54063 |
| 4 | 6 | 0 | 1.979535 | -0.538266 | -0.179013 |
| 5 | 6 | 0 | 2.235193 | 0.728448 | 0.270672 |
| 6 | 6 | 0 | 1.189113 | 1.706231 | 0.392513 |
| 7 | 8 | 0 | 1.266688 | 2.846604 | 0.753435 |
| 8 | 6 | 0 | -1.802071 | -0.237591 | -0.709664 |
| 9 | 6 | 0 | -2.685122 | -0.360465 | 0.556708 |
| 10 | 6 | 0 | -2.379667 | -1.580743 | 1.406846 |
| 11 | 8 | 0 | -2.314888 | 0.820384 | -1.525837 |
| 12 | 8 | 0 | 2.891831 | -1.510958 | -0.332409 |
| 13 | 8 | 0 | -4.035376 | -0.455286 | 0.134386 |
| 14 | 6 | 0 | 4.253985 | -1.228235 | -0.009488 |
| 15 | 1 | 0 | 0.453649 | -1.921529 | -0.909996 |
| 16 | 1 | 0 | 3.2205 | 1.072029 | 0.549265 |
| 17 | 1 | 0 | -1.92239 | -1.149957 | -1.297961 |
| 18 | 1 | 0 | -2.537206 | 0.55506 | 1.151467 |
| 19 | 1 | 0 | -3.095416 | -1.638453 | 2.228908 |
| 20 | 1 | 0 | -1.373459 | -1.538772 | 1.829377 |
| 21 | 1 | 0 | -2.477216 | -2.495422 | 0.815251 |
| 22 | 1 | 0 | -1.98707 | 1.654831 | -1.162917 |
| 23 | 1 | 0 | -4.149694 | 0.208042 | -0.560707 |
| 24 | 1 | 0 | 4.798851 | -2.147217 | -0.211039 |
| 25 | 1 | 0 | 4.643952 | -0.421837 | -0.636174 |
| 26 | 1 | 0 | 4.35748 | -0.960776 | 1.045483 |

**1F-2-3**

**
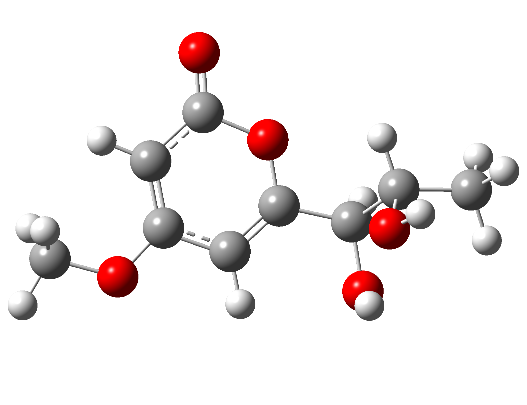
** E = -726.5174404 a.u

| **1F-2-3** | | Standard Orientation  (Ångstroms) | | | |
| --- | --- | --- | --- | --- | --- |
| No. | Atom | Type | X | Y | Z |
| 1 | 8 | 0 | 0.086699 | 1.256647 | -0.327464 |
| 2 | 6 | 0 | 0.311877 | -0.069244 | -0.407148 |
| 3 | 6 | 0 | -0.671688 | -0.980187 | -0.276127 |
| 4 | 6 | 0 | -2.003865 | -0.511048 | -0.03224 |
| 5 | 6 | 0 | -2.260518 | 0.830329 | 0.0591 |
| 6 | 6 | 0 | -1.211414 | 1.801826 | -0.083185 |
| 7 | 8 | 0 | -1.288877 | 2.998686 | -0.022567 |
| 8 | 6 | 0 | 1.7659 | -0.386362 | -0.696717 |
| 9 | 6 | 0 | 2.710915 | 0.266178 | 0.333076 |
| 10 | 6 | 0 | 4.163542 | 0.252612 | -0.127211 |
| 11 | 8 | 0 | 1.993115 | -1.778162 | -0.76171 |
| 12 | 8 | 0 | -2.919876 | -1.488368 | 0.087528 |
| 13 | 8 | 0 | 2.52636 | -0.526594 | 1.516766 |
| 14 | 6 | 0 | -4.277873 | -1.123655 | 0.326603 |
| 15 | 1 | 0 | -0.464662 | -2.035382 | -0.369808 |
| 16 | 1 | 0 | -3.24399 | 1.237604 | 0.241587 |
| 17 | 1 | 0 | 1.996638 | 0.021397 | -1.686708 |
| 18 | 1 | 0 | 2.37781 | 1.292672 | 0.51524 |
| 19 | 1 | 0 | 4.823944 | 0.627033 | 0.661704 |
| 20 | 1 | 0 | 4.303729 | 0.895556 | -1.00058 |
| 21 | 1 | 0 | 4.474371 | -0.76078 | -0.387886 |
| 22 | 1 | 0 | 2.122173 | -2.069045 | 0.153621 |
| 23 | 1 | 0 | 3.144167 | -0.234496 | 2.194681 |
| 24 | 1 | 0 | -4.826929 | -2.060478 | 0.386354 |
| 25 | 1 | 0 | -4.376925 | -0.577147 | 1.268584 |
| 26 | 1 | 0 | -4.670948 | -0.517283 | -0.494119 |

**1F-2-4**

**
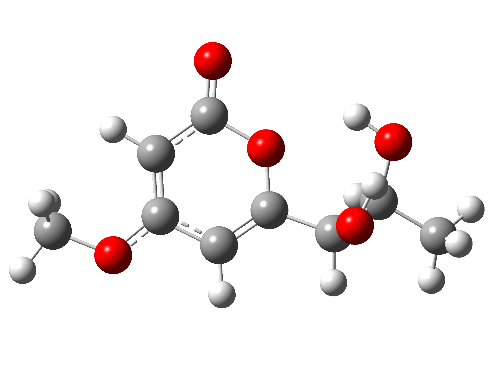
** E = -726.518141 a.u

| **1F-2-4** | | Standard Orientation  (Ångstroms) | | | |
| --- | --- | --- | --- | --- | --- |
| No. | Atom | Type | X | Y | Z |
| 1 | 8 | 0 | 0.191347 | 0.951108 | 0.136948 |
| 2 | 6 | 0 | 0.26706 | -0.401022 | 0.140097 |
| 3 | 6 | 0 | -0.835456 | -1.1687 | 0.036484 |
| 4 | 6 | 0 | -2.11875 | -0.531845 | -0.057296 |
| 5 | 6 | 0 | -2.214745 | 0.833175 | -0.05431 |
| 6 | 6 | 0 | -1.04565 | 1.662495 | 0.031858 |
| 7 | 8 | 0 | -0.970167 | 2.860193 | 0.020448 |
| 8 | 6 | 0 | 1.682426 | -0.912103 | 0.286537 |
| 9 | 6 | 0 | 2.661267 | -0.205579 | -0.691275 |
| 10 | 6 | 0 | 3.97 | -0.96544 | -0.828316 |
| 11 | 8 | 0 | 2.138779 | -0.743712 | 1.618098 |
| 12 | 8 | 0 | -3.150918 | -1.385759 | -0.142571 |
| 13 | 8 | 0 | 2.97917 | 1.086572 | -0.164967 |
| 14 | 6 | 0 | -4.473517 | -0.853638 | -0.226643 |
| 15 | 1 | 0 | -0.771435 | -2.248028 | 0.044745 |
| 16 | 1 | 0 | -3.152786 | 1.363747 | -0.124792 |
| 17 | 1 | 0 | 1.667041 | -1.987303 | 0.087665 |
| 18 | 1 | 0 | 2.183819 | -0.103911 | -1.674646 |
| 19 | 1 | 0 | 4.674101 | -0.384554 | -1.426205 |
| 20 | 1 | 0 | 3.812635 | -1.931752 | -1.314361 |
| 21 | 1 | 0 | 4.411803 | -1.140016 | 0.154667 |
| 22 | 1 | 0 | 2.568201 | 0.12688 | 1.63835 |
| 23 | 1 | 0 | 2.181883 | 1.632551 | -0.20704 |
| 24 | 1 | 0 | -5.132912 | -1.716645 | -0.276906 |
| 25 | 1 | 0 | -4.710806 | -0.2578 | 0.658636 |
| 26 | 1 | 0 | -4.593039 | -0.244449 | -1.12658 |

**1F-2-5**

**
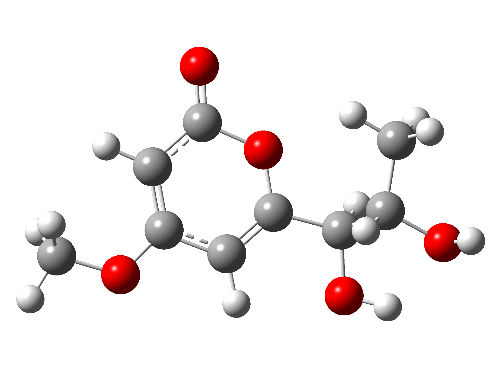
** E = -726.51649 a.u

| **1F-2-5** | | Standard Orientation  (Ångstroms) | | | |
| --- | --- | --- | --- | --- | --- |
| No. | Atom | Type | X | Y | Z |
| 1 | 8 | 0 | 0.101089 | 1.170916 | -0.433659 |
| 2 | 6 | 0 | 0.316237 | -0.157311 | -0.349692 |
| 3 | 6 | 0 | -0.674381 | -1.037068 | -0.10272 |
| 4 | 6 | 0 | -2.004803 | -0.531815 | 0.069377 |
| 5 | 6 | 0 | -2.251099 | 0.812433 | -0.005804 |
| 6 | 6 | 0 | -1.190956 | 1.751147 | -0.24956 |
| 7 | 8 | 0 | -1.255283 | 2.948671 | -0.315551 |
| 8 | 6 | 0 | 1.756372 | -0.532284 | -0.583686 |
| 9 | 6 | 0 | 2.694028 | -0.123346 | 0.577424 |
| 10 | 6 | 0 | 2.927701 | 1.372102 | 0.732268 |
| 11 | 8 | 0 | 1.816267 | -1.931936 | -0.765407 |
| 12 | 8 | 0 | -2.928856 | -1.480763 | 0.299216 |
| 13 | 8 | 0 | 3.924439 | -0.799567 | 0.25393 |
| 14 | 6 | 0 | -4.287216 | -1.081646 | 0.473408 |
| 15 | 1 | 0 | -0.47056 | -2.095875 | -0.060703 |
| 16 | 1 | 0 | -3.232592 | 1.24647 | 0.115849 |
| 17 | 1 | 0 | 2.097308 | -0.007772 | -1.489854 |
| 18 | 1 | 0 | 2.281548 | -0.551885 | 1.501122 |
| 19 | 1 | 0 | 3.637994 | 1.557899 | 1.544371 |
| 20 | 1 | 0 | 2.007338 | 1.910005 | 0.959453 |
| 21 | 1 | 0 | 3.349117 | 1.787398 | -0.186073 |
| 22 | 1 | 0 | 2.744714 | -2.170633 | -0.633544 |
| 23 | 1 | 0 | 4.511979 | -0.76477 | 1.015864 |
| 24 | 1 | 0 | -4.843984 | -2.001146 | 0.638036 |
| 25 | 1 | 0 | -4.394765 | -0.42486 | 1.341155 |
| 26 | 1 | 0 | -4.664651 | -0.576665 | -0.419996 |

**1F-2-6**


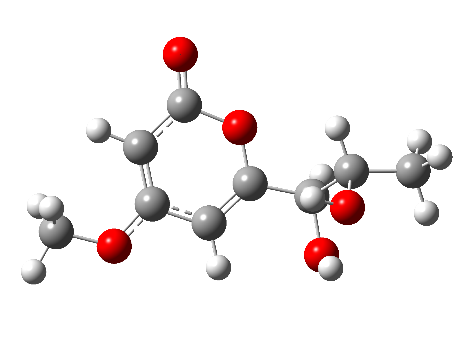
E = -726.5183815 a.u

| **1F-2-6** | | Standard Orientation  (Ångstroms) | | | |
| --- | --- | --- | --- | --- | --- |
| No. | Atom | Type | X | Y | Z |
| 1 | 8 | 0 | 0.077621 | 1.28378 | -0.329556 |
| 2 | 6 | 0 | 0.320975 | -0.039399 | -0.426513 |
| 3 | 6 | 0 | -0.647973 | -0.965571 | -0.283447 |
| 4 | 6 | 0 | -1.98788 | -0.517837 | -0.03241 |
| 5 | 6 | 0 | -2.263857 | 0.819011 | 0.063427 |
| 6 | 6 | 0 | -1.229927 | 1.807931 | -0.07862 |
| 7 | 8 | 0 | -1.325349 | 3.00198 | -0.008069 |
| 8 | 6 | 0 | 1.778611 | -0.334059 | -0.718069 |
| 9 | 6 | 0 | 2.708949 | 0.25192 | 0.376589 |
| 10 | 6 | 0 | 4.165069 | 0.262759 | -0.053697 |
| 11 | 8 | 0 | 2.00748 | -1.719921 | -0.848533 |
| 12 | 8 | 0 | -2.886445 | -1.509644 | 0.088062 |
| 13 | 8 | 0 | 2.616349 | -0.59982 | 1.532488 |
| 14 | 6 | 0 | -4.252401 | -1.168437 | 0.321691 |
| 15 | 1 | 0 | -0.425454 | -2.016131 | -0.394702 |
| 16 | 1 | 0 | -3.253017 | 1.210981 | 0.248815 |
| 17 | 1 | 0 | 2.018851 | 0.13273 | -1.679724 |
| 18 | 1 | 0 | 2.379494 | 1.264423 | 0.62643 |
| 19 | 1 | 0 | 4.795707 | 0.584941 | 0.776333 |
| 20 | 1 | 0 | 4.313739 | 0.948522 | -0.891487 |
| 21 | 1 | 0 | 4.482424 | -0.73428 | -0.365249 |
| 22 | 1 | 0 | 2.222755 | -2.033823 | 0.04438 |
| 23 | 1 | 0 | 1.799949 | -0.398417 | 2.005158 |
| 24 | 1 | 0 | -4.785182 | -2.114706 | 0.377255 |
| 25 | 1 | 0 | -4.364762 | -0.62544 | 1.264139 |
| 26 | 1 | 0 | -4.651149 | -0.567707 | -0.500188 |

**1F-2-7**


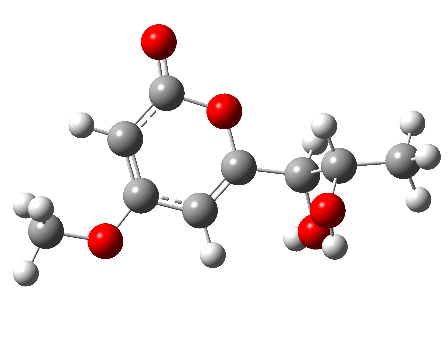
E = -726.515824 a.u

| **1F-2-7** | | Standard Orientation  (Ångstroms) | | | |
| --- | --- | --- | --- | --- | --- |
| No. | Atom | Type | X | Y | Z |
| 1 | 8 | 0 | 0.008517 | 1.40269 | -0.314632 |
| 2 | 6 | 0 | 0.34208 | 0.104108 | -0.43601 |
| 3 | 6 | 0 | -0.557572 | -0.892002 | -0.315813 |
| 4 | 6 | 0 | -1.921981 | -0.547514 | -0.03946 |
| 5 | 6 | 0 | -2.289122 | 0.763302 | 0.093903 |
| 6 | 6 | 0 | -1.328044 | 1.825851 | -0.031661 |
| 7 | 8 | 0 | -1.50914 | 3.008013 | 0.063853 |
| 8 | 6 | 0 | 1.815809 | -0.068928 | -0.706688 |
| 9 | 6 | 0 | 2.663059 | 0.143204 | 0.56968 |
| 10 | 6 | 0 | 4.152068 | 0.252363 | 0.245009 |
| 11 | 8 | 0 | 2.122907 | -1.381302 | -1.164442 |
| 12 | 8 | 0 | -2.746044 | -1.604231 | 0.066621 |
| 13 | 8 | 0 | 2.399962 | -0.894752 | 1.500741 |
| 14 | 6 | 0 | -4.123079 | -1.367513 | 0.357514 |
| 15 | 1 | 0 | -0.257025 | -1.926778 | -0.385787 |
| 16 | 1 | 0 | -3.300598 | 1.080123 | 0.30122 |
| 17 | 1 | 0 | 2.105479 | 0.687529 | -1.448251 |
| 18 | 1 | 0 | 2.320278 | 1.061894 | 1.050605 |
| 19 | 1 | 0 | 4.719406 | 0.350389 | 1.172233 |
| 20 | 1 | 0 | 4.362681 | 1.124321 | -0.381784 |
| 21 | 1 | 0 | 4.500745 | -0.639261 | -0.281909 |
| 22 | 1 | 0 | 1.671729 | -1.542297 | -2.000447 |
| 23 | 1 | 0 | 2.565396 | -1.731683 | 1.047204 |
| 24 | 1 | 0 | -4.58561 | -2.350526 | 0.404918 |
| 25 | 1 | 0 | -4.237563 | -0.858584 | 1.318326 |
| 26 | 1 | 0 | -4.593837 | -0.775694 | -0.432308 |

**1F-2-8**


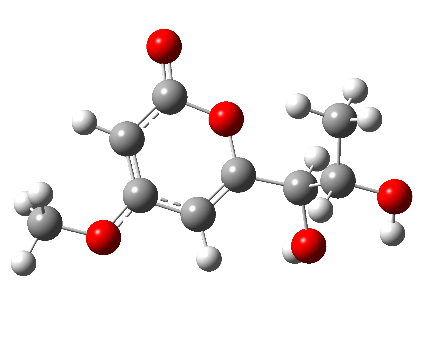
E = -726.5166505 a.u

| **1F-2-8** | | Standard Orientation  (Ångstroms) | | | |
| --- | --- | --- | --- | --- | --- |
| No. | Atom | Type | X | Y | Z |
| 1 | 8 | 0 | 0.062862 | 1.229331 | -0.514677 |
| 2 | 6 | 0 | 0.346213 | -0.08201 | -0.396443 |
| 3 | 6 | 0 | -0.596363 | -1.004181 | -0.108995 |
| 4 | 6 | 0 | -1.947767 | -0.563426 | 0.082827 |
| 5 | 6 | 0 | -2.261913 | 0.763322 | -0.025827 |
| 6 | 6 | 0 | -1.257973 | 1.747549 | -0.32931 |
| 7 | 8 | 0 | -1.39155 | 2.933573 | -0.448688 |
| 8 | 6 | 0 | 1.808342 | -0.384032 | -0.614721 |
| 9 | 6 | 0 | 2.683109 | -0.219112 | 0.649403 |
| 10 | 6 | 0 | 2.728377 | 1.194578 | 1.199698 |
| 11 | 8 | 0 | 1.999169 | -1.746284 | -1.004809 |
| 12 | 8 | 0 | -2.815067 | -1.550888 | 0.363791 |
| 13 | 8 | 0 | 4.012362 | -0.592181 | 0.323052 |
| 14 | 6 | 0 | -4.185086 | -1.213369 | 0.579621 |
| 15 | 1 | 0 | -0.340174 | -2.04991 | -0.022691 |
| 16 | 1 | 0 | -3.261254 | 1.151348 | 0.105758 |
| 17 | 1 | 0 | 2.193859 | 0.298334 | -1.382319 |
| 18 | 1 | 0 | 2.274515 | -0.906081 | 1.406844 |
| 19 | 1 | 0 | 3.421909 | 1.222191 | 2.042192 |
| 20 | 1 | 0 | 1.750162 | 1.532618 | 1.543495 |
| 21 | 1 | 0 | 3.088905 | 1.893753 | 0.441734 |
| 22 | 1 | 0 | 1.67085 | -1.86805 | -1.902635 |
| 23 | 1 | 0 | 3.957284 | -1.453676 | -0.110655 |
| 24 | 1 | 0 | -4.688458 | -2.153619 | 0.791467 |
| 25 | 1 | 0 | -4.291336 | -0.537107 | 1.432159 |
| 26 | 1 | 0 | -4.620106 | -0.754107 | -0.31223 |

**1F-2-9**


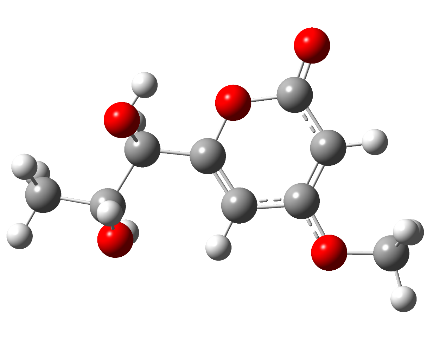
E = -726.515559 a.u

| **1F-2-9** | | Standard Orientation  (Ångstroms) | | | |
| --- | --- | --- | --- | --- | --- |
| No. | Atom | Type | X | Y | Z |
| 1 | 8 | 0 | 0.233462 | 1.566313 | -0.163783 |
| 2 | 6 | 0 | -0.348325 | 0.342254 | -0.19498 |
| 3 | 6 | 0 | 0.373049 | -0.792808 | -0.139149 |
| 4 | 6 | 0 | 1.804202 | -0.691863 | -0.065789 |
| 5 | 6 | 0 | 2.412651 | 0.53258 | -0.033316 |
| 6 | 6 | 0 | 1.645808 | 1.748424 | -0.061712 |
| 7 | 8 | 0 | 2.040909 | 2.880766 | -0.01491 |
| 8 | 6 | 0 | -1.848694 | 0.497725 | -0.274271 |
| 9 | 6 | 0 | -2.643788 | -0.809952 | -0.364708 |
| 10 | 6 | 0 | -4.122634 | -0.548382 | -0.648057 |
| 11 | 8 | 0 | -2.319957 | 1.170539 | 0.898777 |
| 12 | 8 | 0 | 2.434029 | -1.877378 | -0.029197 |
| 13 | 8 | 0 | -2.469319 | -1.578368 | 0.820366 |
| 14 | 6 | 0 | 3.858662 | -1.891519 | 0.057674 |
| 15 | 1 | 0 | -0.10396 | -1.760502 | -0.103012 |
| 16 | 1 | 0 | 3.483099 | 0.664309 | 0.025756 |
| 17 | 1 | 0 | -2.061525 | 1.110787 | -1.162025 |
| 18 | 1 | 0 | -2.224507 | -1.41618 | -1.172873 |
| 19 | 1 | 0 | -4.663387 | -1.49623 | -0.651412 |
| 20 | 1 | 0 | -4.265432 | -0.064648 | -1.619462 |
| 21 | 1 | 0 | -4.553806 | 0.09591 | 0.120892 |
| 22 | 1 | 0 | -1.798097 | 1.973799 | 1.014675 |
| 23 | 1 | 0 | -2.598408 | -0.974358 | 1.564449 |
| 24 | 1 | 0 | 4.139923 | -2.941585 | 0.079782 |
| 25 | 1 | 0 | 4.308466 | -1.406752 | -0.813111 |
| 26 | 1 | 0 | 4.199046 | -1.398289 | 0.972161 |

**1F-2-10**


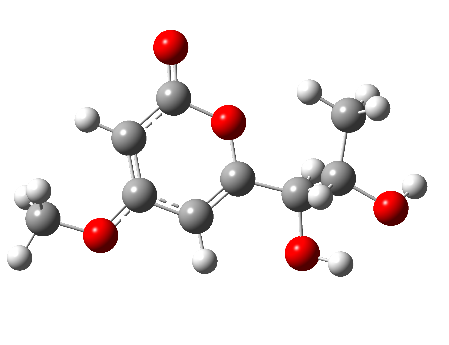
E = -726.5172046 a.u

| **1F-2-10** | | Standard Orientation  (Ångstroms) | | | |
| --- | --- | --- | --- | --- | --- |
| No. | Atom | Type | X | Y | Z |
| 1 | 8 | 0 | 0.095559 | 1.175996 | -0.44596 |
| 2 | 6 | 0 | 0.31465 | -0.152473 | -0.366086 |
| 3 | 6 | 0 | -0.671895 | -1.035708 | -0.115571 |
| 4 | 6 | 0 | -2.002694 | -0.534322 | 0.066051 |
| 5 | 6 | 0 | -2.253337 | 0.809487 | -0.005919 |
| 6 | 6 | 0 | -1.198058 | 1.752006 | -0.255293 |
| 7 | 8 | 0 | -1.266083 | 2.94927 | -0.321231 |
| 8 | 6 | 0 | 1.757526 | -0.516951 | -0.594647 |
| 9 | 6 | 0 | 2.685808 | -0.13795 | 0.594459 |
| 10 | 6 | 0 | 2.926711 | 1.35051 | 0.782785 |
| 11 | 8 | 0 | 1.835135 | -1.909765 | -0.810626 |
| 12 | 8 | 0 | -2.922319 | -1.486012 | 0.300295 |
| 13 | 8 | 0 | 3.925523 | -0.845249 | 0.409667 |
| 14 | 6 | 0 | -4.280503 | -1.091329 | 0.486591 |
| 15 | 1 | 0 | -0.464568 | -2.094053 | -0.077409 |
| 16 | 1 | 0 | -3.23538 | 1.24026 | 0.122656 |
| 17 | 1 | 0 | 2.09945 | 0.034448 | -1.486857 |
| 18 | 1 | 0 | 2.259616 | -0.581003 | 1.498522 |
| 19 | 1 | 0 | 3.610952 | 1.509445 | 1.619048 |
| 20 | 1 | 0 | 2.001973 | 1.893315 | 0.976388 |
| 21 | 1 | 0 | 3.374427 | 1.789288 | -0.1165 |
| 22 | 1 | 0 | 2.739774 | -2.159008 | -0.568145 |
| 23 | 1 | 0 | 4.484368 | -0.330897 | -0.187007 |
| 24 | 1 | 0 | -4.832969 | -2.012881 | 0.654004 |
| 25 | 1 | 0 | -4.382584 | -0.436692 | 1.356571 |
| 26 | 1 | 0 | -4.666839 | -0.585754 | -0.402653 |

3. For **1F-3**

Table S3.1. Gibbs free energies*^a^* and equilibrium populations*^b^* low-energy conformers of **1F-3**.

| Conformers | ∆*G* | *P* (%) |
| --- | --- | --- |
| **1F-3-1** | 0.0028186 | 1.41 |
| **1F-3-2** | 0.0003101 | 20.21 |
| **1F-3-3** | 0.0009411 | 10.35 |
| **1F-3-4** | 0.0002405 | 21.76 |
| **1F-3-5** | 0.0018915 | 3.78 |
| **1F-3-6** | 0 | 28.08 |
| **1F-3-7** | 0.0025575 | 1.87 |
| **1F-3-8** | 0.001731 | 4.48 |
| **1F-3-9** | 0.0011769 | 8.06 |
| *^a^* B3LYP/6-311+G (d), in kcal/mol. *^b^*From ∆*G* values at 298.15K | | |

Table S3.2. Cartesian coordinates for the low-energy reoptimized MMFF conformers of **1F-3** at B3LYP/6-311++G (2d, p) level of theory.

**1F-3-1**

**
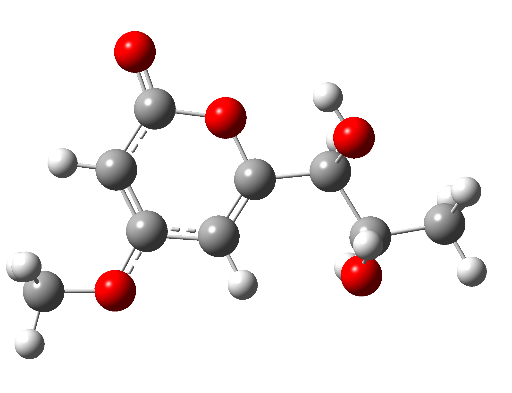
** E = -726.5155629 a.u

| **1F-3-1** | | Standard Orientation  (Ångstroms) | | | |
| --- | --- | --- | --- | --- | --- |
| No. | Atom | Type | X | Y | Z |
| 1 | 8 | 0 | -0.233455 | 1.566495 | -0.1641 |
| 2 | 6 | 0 | 0.348305 | 0.342409 | -0.195121 |
| 3 | 6 | 0 | -0.372971 | -0.792656 | -0.139203 |
| 4 | 6 | 0 | -1.804075 | -0.691831 | -0.065704 |
| 5 | 6 | 0 | -2.412499 | 0.532534 | -0.033179 |
| 6 | 6 | 0 | -1.645532 | 1.74829 | -0.061698 |
| 7 | 8 | 0 | -2.04101 | 2.880546 | -0.014537 |
| 8 | 6 | 0 | 1.848634 | 0.497714 | -0.27444 |
| 9 | 6 | 0 | 2.643465 | -0.810122 | -0.364525 |
| 10 | 6 | 0 | 4.122314 | -0.54867 | -0.648072 |
| 11 | 8 | 0 | 2.319849 | 1.170681 | 0.898417 |
| 12 | 8 | 0 | -2.433819 | -1.877489 | -0.029065 |
| 13 | 8 | 0 | 2.46901 | -1.578081 | 0.820887 |
| 14 | 6 | 0 | -3.858458 | -1.891447 | 0.057457 |
| 15 | 1 | 0 | 0.10411 | -1.760338 | -0.103217 |
| 16 | 1 | 0 | -3.482924 | 0.664207 | 0.026267 |
| 17 | 1 | 0 | 2.06165 | 1.110358 | -1.162489 |
| 18 | 1 | 0 | 2.224205 | -1.41673 | -1.172352 |
| 19 | 1 | 0 | 4.663016 | -1.496573 | -0.650762 |
| 20 | 1 | 0 | 4.265221 | -0.065654 | -1.619822 |
| 21 | 1 | 0 | 4.553584 | 0.096006 | 0.120493 |
| 22 | 1 | 0 | 1.798964 | 1.974716 | 1.013384 |
| 23 | 1 | 0 | 2.599366 | -0.973946 | 1.564658 |
| 24 | 1 | 0 | -4.139874 | -2.941505 | 0.078975 |
| 25 | 1 | 0 | -4.198993 | -1.398771 | 0.972184 |
| 26 | 1 | 0 | -4.308027 | -1.40631 | -0.813233 |

**1F-3-2**


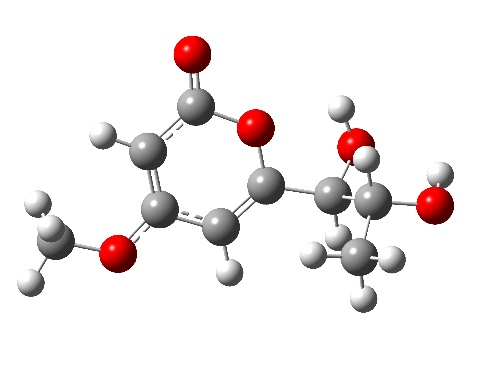
 E = -726.5180714 a.u

| **1F-3-2** | | Standard Orientation  (Ångstroms) | | | |
| --- | --- | --- | --- | --- | --- |
| No. | Atom | Type | X | Y | Z |
| 1 | 8 | 0 | 0.117079 | 1.23658 | 0.033285 |
| 2 | 6 | 0 | 0.345572 | -0.018282 | -0.411916 |
| 3 | 6 | 0 | -0.644375 | -0.923526 | -0.54063 |
| 4 | 6 | 0 | -1.979535 | -0.538266 | -0.179013 |
| 5 | 6 | 0 | -2.235193 | 0.728448 | 0.270672 |
| 6 | 6 | 0 | -1.189113 | 1.706231 | 0.392513 |
| 7 | 8 | 0 | -1.266688 | 2.846604 | 0.753435 |
| 8 | 6 | 0 | 1.802071 | -0.237591 | -0.709664 |
| 9 | 6 | 0 | 2.685122 | -0.360465 | 0.556708 |
| 10 | 6 | 0 | 2.379667 | -1.580743 | 1.406846 |
| 11 | 8 | 0 | 2.314888 | 0.820384 | -1.525837 |
| 12 | 8 | 0 | -2.891831 | -1.510958 | -0.332409 |
| 13 | 8 | 0 | 4.035376 | -0.455286 | 0.134386 |
| 14 | 6 | 0 | -4.253985 | -1.228235 | -0.009488 |
| 15 | 1 | 0 | -0.453649 | -1.921529 | -0.909996 |
| 16 | 1 | 0 | -3.2205 | 1.072029 | 0.549265 |
| 17 | 1 | 0 | 1.92239 | -1.149957 | -1.297961 |
| 18 | 1 | 0 | 2.537206 | 0.55506 | 1.151467 |
| 19 | 1 | 0 | 3.095416 | -1.638453 | 2.228908 |
| 20 | 1 | 0 | 1.373459 | -1.538772 | 1.829377 |
| 21 | 1 | 0 | 2.477216 | -2.495422 | 0.815251 |
| 22 | 1 | 0 | 1.98707 | 1.654831 | -1.162917 |
| 23 | 1 | 0 | 4.149694 | 0.208042 | -0.560707 |
| 24 | 1 | 0 | -4.798851 | -2.147217 | -0.211039 |
| 25 | 1 | 0 | -4.35748 | -0.960776 | 1.045483 |
| 26 | 1 | 0 | -4.643952 | -0.421837 | -0.636174 |

**1F-3-3**

**
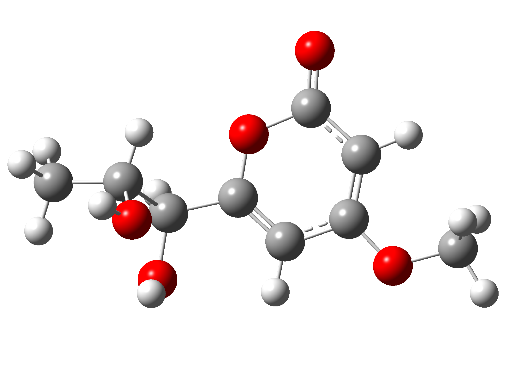
** E = -726.5174404 a.u

| **1F-3-3** | | Standard Orientation  (Ångstroms) | | | |
| --- | --- | --- | --- | --- | --- |
| No. | Atom | Type | X | Y | Z |
| 1 | 8 | 0 | -0.086699 | 1.256647 | -0.327464 |
| 2 | 6 | 0 | -0.311877 | -0.069244 | -0.407148 |
| 3 | 6 | 0 | 0.671688 | -0.980187 | -0.276127 |
| 4 | 6 | 0 | 2.003865 | -0.511048 | -0.03224 |
| 5 | 6 | 0 | 2.260518 | 0.830329 | 0.0591 |
| 6 | 6 | 0 | 1.211414 | 1.801826 | -0.083185 |
| 7 | 8 | 0 | 1.288877 | 2.998686 | -0.022567 |
| 8 | 6 | 0 | -1.7659 | -0.386362 | -0.696717 |
| 9 | 6 | 0 | -2.710915 | 0.266178 | 0.333076 |
| 10 | 6 | 0 | -4.163542 | 0.252612 | -0.127211 |
| 11 | 8 | 0 | -1.993115 | -1.778162 | -0.76171 |
| 12 | 8 | 0 | 2.919876 | -1.488368 | 0.087528 |
| 13 | 8 | 0 | -2.52636 | -0.526594 | 1.516766 |
| 14 | 6 | 0 | 4.277873 | -1.123655 | 0.326603 |
| 15 | 1 | 0 | 0.464662 | -2.035382 | -0.369808 |
| 16 | 1 | 0 | 3.24399 | 1.237604 | 0.241587 |
| 17 | 1 | 0 | -1.996638 | 0.021397 | -1.686708 |
| 18 | 1 | 0 | -2.37781 | 1.292672 | 0.51524 |
| 19 | 1 | 0 | -4.823944 | 0.627033 | 0.661704 |
| 20 | 1 | 0 | -4.303729 | 0.895556 | -1.00058 |
| 21 | 1 | 0 | -4.474371 | -0.76078 | -0.387886 |
| 22 | 1 | 0 | -2.122173 | -2.069045 | 0.153621 |
| 23 | 1 | 0 | -3.144167 | -0.234496 | 2.194681 |
| 24 | 1 | 0 | 4.826929 | -2.060478 | 0.386354 |
| 25 | 1 | 0 | 4.670948 | -0.517283 | -0.494119 |
| 26 | 1 | 0 | 4.376925 | -0.577147 | 1.268584 |

**1F-3-4**

**
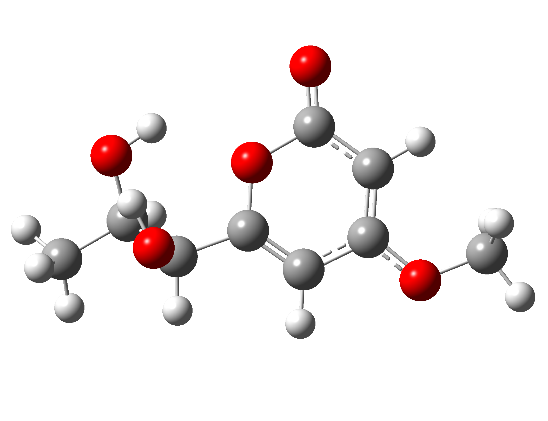
** E = -726.518141 a.u

| **1F-3-4** | | Standard Orientation  (Ångstroms) | | | |
| --- | --- | --- | --- | --- | --- |
| No. | Atom | Type | X | Y | Z |
| 1 | 8 | 0 | -0.191347 | 0.951108 | 0.136948 |
| 2 | 6 | 0 | -0.26706 | -0.401022 | 0.140097 |
| 3 | 6 | 0 | 0.835456 | -1.1687 | 0.036484 |
| 4 | 6 | 0 | 2.11875 | -0.531845 | -0.057296 |
| 5 | 6 | 0 | 2.214745 | 0.833175 | -0.05431 |
| 6 | 6 | 0 | 1.04565 | 1.662495 | 0.031858 |
| 7 | 8 | 0 | 0.970167 | 2.860193 | 0.020448 |
| 8 | 6 | 0 | -1.682426 | -0.912103 | 0.286537 |
| 9 | 6 | 0 | -2.661267 | -0.205579 | -0.691275 |
| 10 | 6 | 0 | -3.97 | -0.96544 | -0.828316 |
| 11 | 8 | 0 | -2.138779 | -0.743712 | 1.618098 |
| 12 | 8 | 0 | 3.150918 | -1.385759 | -0.142571 |
| 13 | 8 | 0 | -2.97917 | 1.086572 | -0.164967 |
| 14 | 6 | 0 | 4.473517 | -0.853638 | -0.226643 |
| 15 | 1 | 0 | 0.771435 | -2.248028 | 0.044745 |
| 16 | 1 | 0 | 3.152786 | 1.363747 | -0.124792 |
| 17 | 1 | 0 | -1.667041 | -1.987303 | 0.087665 |
| 18 | 1 | 0 | -2.183819 | -0.103911 | -1.674646 |
| 19 | 1 | 0 | -4.674101 | -0.384554 | -1.426205 |
| 20 | 1 | 0 | -3.812635 | -1.931752 | -1.314361 |
| 21 | 1 | 0 | -4.411803 | -1.140016 | 0.154667 |
| 22 | 1 | 0 | -2.568201 | 0.12688 | 1.63835 |
| 23 | 1 | 0 | -2.181883 | 1.632551 | -0.20704 |
| 24 | 1 | 0 | 5.132912 | -1.716645 | -0.276906 |
| 25 | 1 | 0 | 4.593039 | -0.244449 | -1.12658 |
| 26 | 1 | 0 | 4.710806 | -0.2578 | 0.658636 |

**1F-3-5**

**
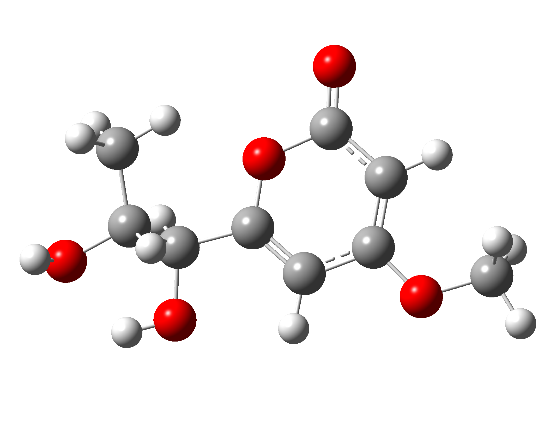
** E = -726.51649 a.u

| **1F-3-5** | | Standard Orientation  (Ångstroms) | | | |
| --- | --- | --- | --- | --- | --- |
| No. | Atom | Type | X | Y | Z |
| 1 | 8 | 0 | -0.101089 | 1.170916 | -0.433659 |
| 2 | 6 | 0 | -0.316237 | -0.157311 | -0.349692 |
| 3 | 6 | 0 | 0.674381 | -1.037068 | -0.10272 |
| 4 | 6 | 0 | 2.004803 | -0.531815 | 0.069377 |
| 5 | 6 | 0 | 2.251099 | 0.812433 | -0.005804 |
| 6 | 6 | 0 | 1.190956 | 1.751147 | -0.24956 |
| 7 | 8 | 0 | 1.255283 | 2.948671 | -0.315551 |
| 8 | 6 | 0 | -1.756372 | -0.532284 | -0.583686 |
| 9 | 6 | 0 | -2.694028 | -0.123346 | 0.577424 |
| 10 | 6 | 0 | -2.927701 | 1.372102 | 0.732268 |
| 11 | 8 | 0 | -1.816267 | -1.931936 | -0.765407 |
| 12 | 8 | 0 | 2.928856 | -1.480763 | 0.299216 |
| 13 | 8 | 0 | -3.924439 | -0.799567 | 0.25393 |
| 14 | 6 | 0 | 4.287216 | -1.081646 | 0.473408 |
| 15 | 1 | 0 | 0.47056 | -2.095875 | -0.060703 |
| 16 | 1 | 0 | 3.232592 | 1.24647 | 0.115849 |
| 17 | 1 | 0 | -2.097308 | -0.007772 | -1.489854 |
| 18 | 1 | 0 | -2.281548 | -0.551885 | 1.501122 |
| 19 | 1 | 0 | -3.637994 | 1.557899 | 1.544371 |
| 20 | 1 | 0 | -2.007338 | 1.910005 | 0.959453 |
| 21 | 1 | 0 | -3.349117 | 1.787398 | -0.186073 |
| 22 | 1 | 0 | -2.744714 | -2.170633 | -0.633544 |
| 23 | 1 | 0 | -4.511979 | -0.76477 | 1.015864 |
| 24 | 1 | 0 | 4.843984 | -2.001146 | 0.638036 |
| 25 | 1 | 0 | 4.664651 | -0.576665 | -0.419996 |
| 26 | 1 | 0 | 4.394765 | -0.42486 | 1.341155 |

**1F-3-6**


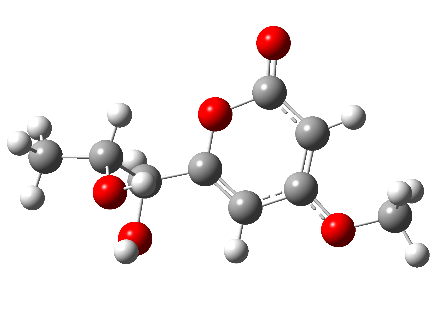
E = -726.5183815 a.u

| **1F-3-5** | | Standard Orientation  (Ångstroms) | | | |
| --- | --- | --- | --- | --- | --- |
| No. | Atom | Type | X | Y | Z |
| 1 | 8 | 0 | -0.077621 | 1.28378 | -0.329556 |
| 2 | 6 | 0 | -0.320975 | -0.039399 | -0.426513 |
| 3 | 6 | 0 | 0.647973 | -0.965571 | -0.283447 |
| 4 | 6 | 0 | 1.98788 | -0.517837 | -0.03241 |
| 5 | 6 | 0 | 2.263857 | 0.819011 | 0.063427 |
| 6 | 6 | 0 | 1.229927 | 1.807931 | -0.07862 |
| 7 | 8 | 0 | 1.325349 | 3.00198 | -0.008069 |
| 8 | 6 | 0 | -1.778611 | -0.334059 | -0.718069 |
| 9 | 6 | 0 | -2.708949 | 0.25192 | 0.376589 |
| 10 | 6 | 0 | -4.165069 | 0.262759 | -0.053697 |
| 11 | 8 | 0 | -2.00748 | -1.719921 | -0.848533 |
| 12 | 8 | 0 | 2.886445 | -1.509644 | 0.088062 |
| 13 | 8 | 0 | -2.616349 | -0.59982 | 1.532488 |
| 14 | 6 | 0 | 4.252401 | -1.168437 | 0.321691 |
| 15 | 1 | 0 | 0.425454 | -2.016131 | -0.394702 |
| 16 | 1 | 0 | 3.253017 | 1.210981 | 0.248815 |
| 17 | 1 | 0 | -2.018851 | 0.13273 | -1.679724 |
| 18 | 1 | 0 | -2.379494 | 1.264423 | 0.62643 |
| 19 | 1 | 0 | -4.795707 | 0.584941 | 0.776333 |
| 20 | 1 | 0 | -4.313739 | 0.948522 | -0.891487 |
| 21 | 1 | 0 | -4.482424 | -0.73428 | -0.365249 |
| 22 | 1 | 0 | -2.222755 | -2.033823 | 0.04438 |
| 23 | 1 | 0 | -1.799949 | -0.398417 | 2.005158 |
| 24 | 1 | 0 | 4.785182 | -2.114706 | 0.377255 |
| 25 | 1 | 0 | 4.651149 | -0.567707 | -0.500188 |
| 26 | 1 | 0 | 4.364762 | -0.62544 | 1.264139 |

**1F-3-7**


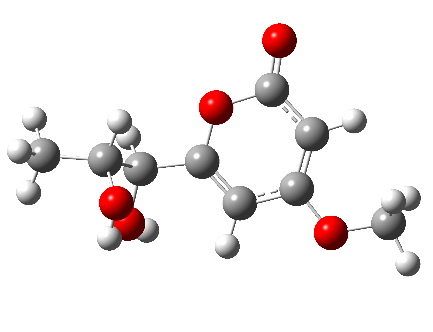
E = -726.515824 a.u

| **1F-3-7** | | Standard Orientation  (Ångstroms) | | | |
| --- | --- | --- | --- | --- | --- |
| No. | Atom | Type | X | Y | Z |
| 1 | 8 | 0 | -0.008517 | 1.40269 | -0.314632 |
| 2 | 6 | 0 | -0.34208 | 0.104108 | -0.43601 |
| 3 | 6 | 0 | 0.557572 | -0.892002 | -0.315813 |
| 4 | 6 | 0 | 1.921981 | -0.547514 | -0.03946 |
| 5 | 6 | 0 | 2.289122 | 0.763302 | 0.093903 |
| 6 | 6 | 0 | 1.328044 | 1.825851 | -0.031661 |
| 7 | 8 | 0 | 1.50914 | 3.008013 | 0.063853 |
| 8 | 6 | 0 | -1.815809 | -0.068928 | -0.706688 |
| 9 | 6 | 0 | -2.663059 | 0.143204 | 0.56968 |
| 10 | 6 | 0 | -4.152068 | 0.252363 | 0.245009 |
| 11 | 8 | 0 | -2.122907 | -1.381302 | -1.164442 |
| 12 | 8 | 0 | 2.746044 | -1.604231 | 0.066621 |
| 13 | 8 | 0 | -2.399962 | -0.894752 | 1.500741 |
| 14 | 6 | 0 | 4.123079 | -1.367513 | 0.357514 |
| 15 | 1 | 0 | 0.257025 | -1.926778 | -0.385787 |
| 16 | 1 | 0 | 3.300598 | 1.080123 | 0.30122 |
| 17 | 1 | 0 | -2.105479 | 0.687529 | -1.448251 |
| 18 | 1 | 0 | -2.320278 | 1.061894 | 1.050605 |
| 19 | 1 | 0 | -4.719406 | 0.350389 | 1.172233 |
| 20 | 1 | 0 | -4.362681 | 1.124321 | -0.381784 |
| 21 | 1 | 0 | -4.500745 | -0.639261 | -0.281909 |
| 22 | 1 | 0 | -1.671729 | -1.542297 | -2.000447 |
| 23 | 1 | 0 | -2.565396 | -1.731683 | 1.047204 |
| 24 | 1 | 0 | 4.58561 | -2.350526 | 0.404918 |
| 25 | 1 | 0 | 4.593837 | -0.775694 | -0.432308 |
| 26 | 1 | 0 | 4.237563 | -0.858584 | 1.318326 |

**1F-3-8**

**
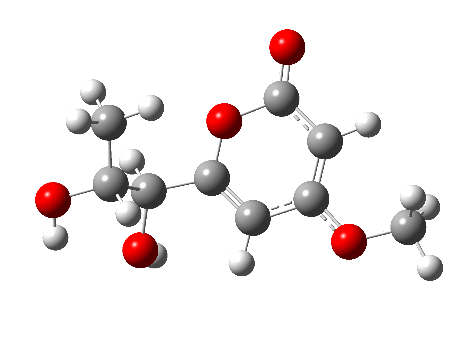
**

E = -726.5166505 a.u

| **1F-3-8** | | Standard Orientation  (Ångstroms) | | | |
| --- | --- | --- | --- | --- | --- |
| No. | Atom | Type | X | Y | Z |
| 1 | 8 | 0 | -0.062862 | 1.229331 | -0.514677 |
| 2 | 6 | 0 | -0.346213 | -0.08201 | -0.396443 |
| 3 | 6 | 0 | 0.596363 | -1.004181 | -0.108995 |
| 4 | 6 | 0 | 1.947767 | -0.563426 | 0.082827 |
| 5 | 6 | 0 | 2.261913 | 0.763322 | -0.025827 |
| 6 | 6 | 0 | 1.257973 | 1.747549 | -0.32931 |
| 7 | 8 | 0 | 1.39155 | 2.933573 | -0.448688 |
| 8 | 6 | 0 | -1.808342 | -0.384032 | -0.614721 |
| 9 | 6 | 0 | -2.683109 | -0.219112 | 0.649403 |
| 10 | 6 | 0 | -2.728377 | 1.194578 | 1.199698 |
| 11 | 8 | 0 | -1.999169 | -1.746284 | -1.004809 |
| 12 | 8 | 0 | 2.815067 | -1.550888 | 0.363791 |
| 13 | 8 | 0 | -4.012362 | -0.592181 | 0.323052 |
| 14 | 6 | 0 | 4.185086 | -1.213369 | 0.579621 |
| 15 | 1 | 0 | 0.340174 | -2.04991 | -0.022691 |
| 16 | 1 | 0 | 3.261254 | 1.151348 | 0.105758 |
| 17 | 1 | 0 | -2.193859 | 0.298334 | -1.382319 |
| 18 | 1 | 0 | -2.274515 | -0.906081 | 1.406844 |
| 19 | 1 | 0 | -3.421909 | 1.222191 | 2.042192 |
| 20 | 1 | 0 | -1.750162 | 1.532618 | 1.543495 |
| 21 | 1 | 0 | -3.088905 | 1.893753 | 0.441734 |
| 22 | 1 | 0 | -1.67085 | -1.86805 | -1.902635 |
| 23 | 1 | 0 | -3.957284 | -1.453676 | -0.110655 |
| 24 | 1 | 0 | 4.688458 | -2.153619 | 0.791467 |
| 25 | 1 | 0 | 4.620106 | -0.754107 | -0.31223 |
| 26 | 1 | 0 | 4.291336 | -0.537107 | 1.432159 |

**1F-3-9**


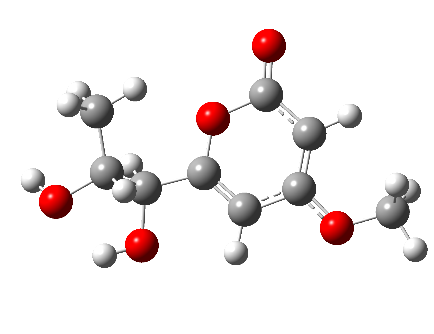
E = -726.5172046 a.u

| **1F-3-9** | | Standard Orientation  (Ångstroms) | | | |
| --- | --- | --- | --- | --- | --- |
| No. | Atom | Type | X | Y | Z |
| 1 | 8 | 0 | -0.095559 | 1.175996 | -0.44596 |
| 2 | 6 | 0 | -0.31465 | -0.152473 | -0.366086 |
| 3 | 6 | 0 | 0.671895 | -1.035708 | -0.115571 |
| 4 | 6 | 0 | 2.002694 | -0.534322 | 0.066051 |
| 5 | 6 | 0 | 2.253337 | 0.809487 | -0.005919 |
| 6 | 6 | 0 | 1.198058 | 1.752006 | -0.255293 |
| 7 | 8 | 0 | 1.266083 | 2.94927 | -0.321231 |
| 8 | 6 | 0 | -1.757526 | -0.516951 | -0.594647 |
| 9 | 6 | 0 | -2.685808 | -0.13795 | 0.594459 |
| 10 | 6 | 0 | -2.926711 | 1.35051 | 0.782785 |
| 11 | 8 | 0 | -1.835135 | -1.909765 | -0.810626 |
| 12 | 8 | 0 | 2.922319 | -1.486012 | 0.300295 |
| 13 | 8 | 0 | -3.925523 | -0.845249 | 0.409667 |
| 14 | 6 | 0 | 4.280503 | -1.091329 | 0.486591 |
| 15 | 1 | 0 | 0.464568 | -2.094053 | -0.077409 |
| 16 | 1 | 0 | 3.23538 | 1.24026 | 0.122656 |
| 17 | 1 | 0 | -2.09945 | 0.034448 | -1.486857 |
| 18 | 1 | 0 | -2.259616 | -0.581003 | 1.498522 |
| 19 | 1 | 0 | -3.610952 | 1.509445 | 1.619048 |
| 20 | 1 | 0 | -2.001973 | 1.893315 | 0.976388 |
| 21 | 1 | 0 | -3.374427 | 1.789288 | -0.1165 |
| 22 | 1 | 0 | -2.739774 | -2.159008 | -0.568145 |
| 23 | 1 | 0 | -4.484368 | -0.330897 | -0.187007 |
| 24 | 1 | 0 | 4.832969 | -2.012881 | 0.654004 |
| 25 | 1 | 0 | 4.666839 | -0.585754 | -0.402653 |
| 26 | 1 | 0 | 4.382584 | -0.436692 | 1.356571 |

4. For **1F-4**

Table S4.1. Gibbs free energies*^a^* and equilibrium populations*^b^* low-energy conformers of **1F-4**.

| Conformers | ∆*G* | *P* (%) |
| --- | --- | --- |
| **1F-4-1** | 0 | 50.54 |
| **1F-4-2** | 0.0001236 | 44.33 |
| **1F-4-3** | 0.0027134 | 2.85 |
| **1F-4-4** | 0.0035226 | 1.21 |
| **1F-4-5** | 0.0036288 | 1.08 |

*^a^* B3LYP/6-311+G (d), in kcal/mol. *^b^*From ∆*G* values at 298.15K

Table S4.2. Cartesian coordinates for the low-energy reoptimized MMFF conformers of **1F-4** at B3LYP/6-311++G (2d, p) level of theory.

**1F-4-1**


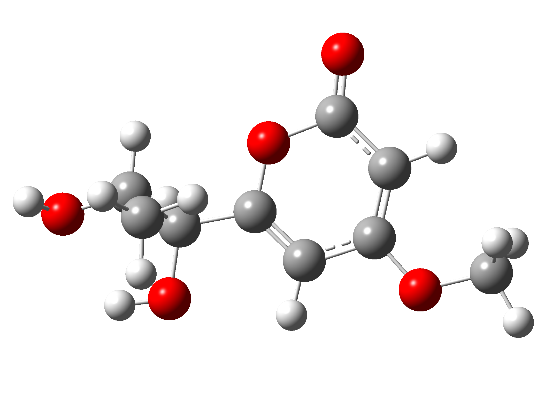
 E = -726.5173073 a.u

| **1F-4-1** | | Standard Orientation  (Ångstroms) | | | |
| --- | --- | --- | --- | --- | --- |
| No. | Atom | Type | X | Y | Z |
| 1 | 8 | 0 | -0.074481 | 1.279794 | -0.39724 |
| 2 | 6 | 0 | -0.335929 | -0.042574 | -0.441734 |
| 3 | 6 | 0 | 0.613181 | -0.9803 | -0.258339 |
| 4 | 6 | 0 | 1.957631 | -0.544379 | -0.01679 |
| 5 | 6 | 0 | 2.254533 | 0.790831 | 0.032581 |
| 6 | 6 | 0 | 1.237367 | 1.789865 | -0.150567 |
| 7 | 8 | 0 | 1.352355 | 2.984569 | -0.120874 |
| 8 | 6 | 0 | -1.783121 | -0.315854 | -0.732734 |
| 9 | 6 | 0 | -2.737635 | 0.317759 | 0.301513 |
| 10 | 6 | 0 | -2.500811 | -0.149811 | 1.732265 |
| 11 | 8 | 0 | -1.980299 | -1.713824 | -0.801088 |
| 12 | 8 | 0 | 2.840872 | -1.544732 | 0.145672 |
| 13 | 8 | 0 | -4.030957 | -0.089246 | -0.174999 |
| 14 | 6 | 0 | 4.208378 | -1.215012 | 0.382329 |
| 15 | 1 | 0 | 0.365879 | -2.029887 | -0.304905 |
| 16 | 1 | 0 | 3.248544 | 1.173968 | 0.210351 |
| 17 | 1 | 0 | -2.020826 | 0.150564 | -1.701035 |
| 18 | 1 | 0 | -2.639927 | 1.406998 | 0.236545 |
| 19 | 1 | 0 | -3.270097 | 0.253567 | 2.399071 |
| 20 | 1 | 0 | -1.534595 | 0.195033 | 2.107754 |
| 21 | 1 | 0 | -2.528012 | -1.239282 | 1.790687 |
| 22 | 1 | 0 | -2.937702 | -1.83967 | -0.848824 |
| 23 | 1 | 0 | -4.695951 | 0.149142 | 0.479322 |
| 24 | 1 | 0 | 4.726878 | -2.166171 | 0.477306 |
| 25 | 1 | 0 | 4.625435 | -0.649381 | -0.45539 |
| 26 | 1 | 0 | 4.31889 | -0.640517 | 1.306214 |

**1F-4-2**

**
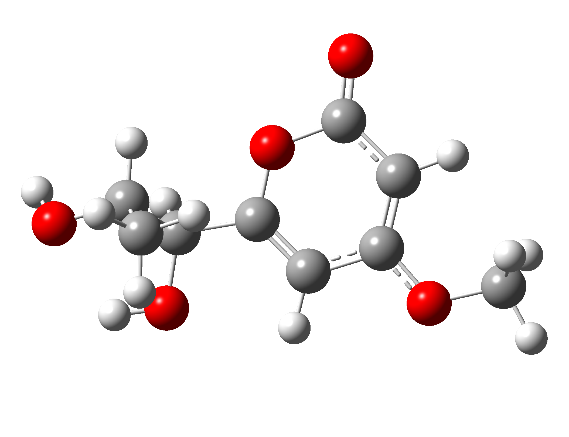
** E = -726.5171837 a.u

| **1F-4-2** | | Standard Orientation  (Ångstroms) | | | |
| --- | --- | --- | --- | --- | --- |
| No. | Atom | Type | X | Y | Z |
| 1 | 8 | 0 | -0.062753 | 1.302815 | -0.381947 |
| 2 | 6 | 0 | -0.337185 | -0.016478 | -0.448931 |
| 3 | 6 | 0 | 0.601992 | -0.967078 | -0.281947 |
| 4 | 6 | 0 | 1.949935 | -0.548748 | -0.028289 |
| 5 | 6 | 0 | 2.259598 | 0.782655 | 0.045155 |
| 6 | 6 | 0 | 1.253618 | 1.795161 | -0.12489 |
| 7 | 8 | 0 | 1.381417 | 2.988114 | -0.078324 |
| 8 | 6 | 0 | -1.789358 | -0.262427 | -0.735884 |
| 9 | 6 | 0 | -2.735877 | 0.296377 | 0.36044 |
| 10 | 6 | 0 | -2.475601 | -0.267754 | 1.745811 |
| 11 | 8 | 0 | -2.005211 | -1.646725 | -0.904444 |
| 12 | 8 | 0 | 2.822723 | -1.560148 | 0.118874 |
| 13 | 8 | 0 | -4.070797 | -0.096621 | -0.006028 |
| 14 | 6 | 0 | 4.192722 | -1.248392 | 0.366946 |
| 15 | 1 | 0 | 0.344168 | -2.01311 | -0.348986 |
| 16 | 1 | 0 | 3.257089 | 1.15214 | 0.232022 |
| 17 | 1 | 0 | -2.026449 | 0.277745 | -1.66907 |
| 18 | 1 | 0 | -2.651068 | 1.387704 | 0.373754 |
| 19 | 1 | 0 | -3.244278 | 0.085656 | 2.434781 |
| 20 | 1 | 0 | -1.500164 | 0.048389 | 2.120714 |
| 21 | 1 | 0 | -2.50088 | -1.358841 | 1.729574 |
| 22 | 1 | 0 | -2.962228 | -1.772143 | -0.827221 |
| 23 | 1 | 0 | -4.382894 | 0.483461 | -0.710516 |
| 24 | 1 | 0 | 4.700941 | -2.206359 | 0.447729 |
| 25 | 1 | 0 | 4.618933 | -0.672922 | -0.45935 |
| 26 | 1 | 0 | 4.304726 | -0.691105 | 1.301061 |

**1F-4-3**

**
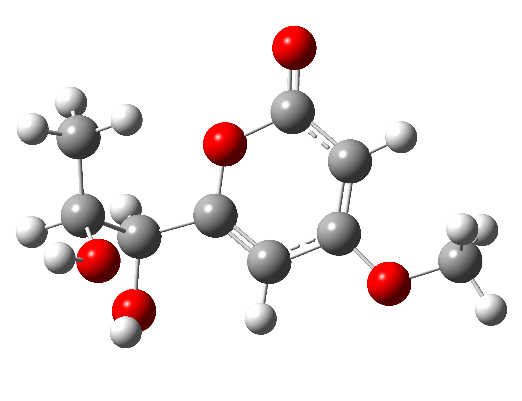
** E = -726.5145939 a.u

| **1F-4-3** | | Standard Orientation  (Ångstroms) | | | |
| --- | --- | --- | --- | --- | --- |
| No. | Atom | Type | X | Y | Z |
| 1 | 8 | 0 | -0.230508 | 1.184186 | -0.475416 |
| 2 | 6 | 0 | -0.410854 | -0.151508 | -0.489841 |
| 3 | 6 | 0 | 0.597543 | -1.016283 | -0.264694 |
| 4 | 6 | 0 | 1.899861 | -0.487963 | 0.014456 |
| 5 | 6 | 0 | 2.107755 | 0.864414 | 0.045581 |
| 6 | 6 | 0 | 1.033947 | 1.787641 | -0.195693 |
| 7 | 8 | 0 | 1.066256 | 2.988342 | -0.191168 |
| 8 | 6 | 0 | -1.834138 | -0.543627 | -0.835819 |
| 9 | 6 | 0 | -2.892284 | -0.148903 | 0.217377 |
| 10 | 6 | 0 | -2.97416 | 1.316685 | 0.618554 |
| 11 | 8 | 0 | -1.934673 | -1.935612 | -1.065714 |
| 12 | 8 | 0 | 2.840652 | -1.425194 | 0.227347 |
| 13 | 8 | 0 | -2.601579 | -0.998763 | 1.340309 |
| 14 | 6 | 0 | 4.173197 | -1.001141 | 0.507454 |
| 15 | 1 | 0 | 0.437229 | -2.082004 | -0.316619 |
| 16 | 1 | 0 | 3.067267 | 1.316092 | 0.25009 |
| 17 | 1 | 0 | -2.087566 | -0.042009 | -1.775675 |
| 18 | 1 | 0 | -3.850103 | -0.459422 | -0.223036 |
| 19 | 1 | 0 | -3.832182 | 1.46932 | 1.281654 |
| 20 | 1 | 0 | -3.11209 | 1.960615 | -0.253599 |
| 21 | 1 | 0 | -2.074523 | 1.642077 | 1.138086 |
| 22 | 1 | 0 | -2.090388 | -2.334396 | -0.196751 |
| 23 | 1 | 0 | -3.328025 | -0.947919 | 1.970259 |
| 24 | 1 | 0 | 4.74984 | -1.913692 | 0.639556 |
| 25 | 1 | 0 | 4.583627 | -0.422776 | -0.324875 |
| 26 | 1 | 0 | 4.210524 | -0.405452 | 1.423788 |

**1F-4-4**

**
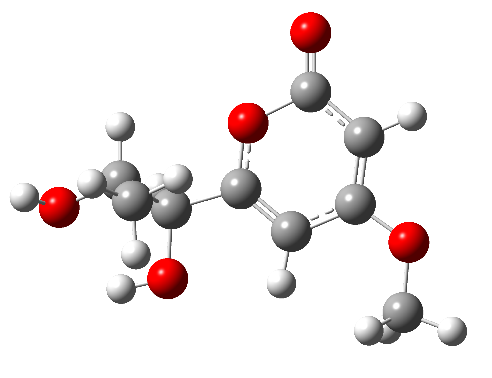
** E = = -726.5137847 a.u

| **1F-4-4** | | Standard Orientation  (Ångstroms) | | | |
| --- | --- | --- | --- | --- | --- |
| No. | Atom | Type | X | Y | Z |
| 1 | 8 | 0 | -0.062458 | 1.408649 | -0.381983 |
| 2 | 6 | 0 | -0.206799 | 0.078084 | -0.428657 |
| 3 | 6 | 0 | 0.813577 | -0.784283 | -0.221054 |
| 4 | 6 | 0 | 2.108386 | -0.234618 | 0.054702 |
| 5 | 6 | 0 | 2.284427 | 1.122638 | 0.109398 |
| 6 | 6 | 0 | 1.20727 | 2.038694 | -0.100266 |
| 7 | 8 | 0 | 1.205814 | 3.237822 | -0.074689 |
| 8 | 6 | 0 | -1.617094 | -0.328183 | -0.749516 |
| 9 | 6 | 0 | -2.649735 | 0.24017 | 0.247009 |
| 10 | 6 | 0 | -2.404718 | -0.167232 | 1.694666 |
| 11 | 8 | 0 | -1.690649 | -1.74014 | -0.791261 |
| 12 | 8 | 0 | 3.193068 | -1.004328 | 0.271008 |
| 13 | 8 | 0 | -3.888 | -0.296906 | -0.245855 |
| 14 | 6 | 0 | 3.07263 | -2.425452 | 0.220548 |
| 15 | 1 | 0 | 0.611459 | -1.841578 | -0.279992 |
| 16 | 1 | 0 | 3.258725 | 1.543625 | 0.317235 |
| 17 | 1 | 0 | -1.869395 | 0.094218 | -1.733971 |
| 18 | 1 | 0 | -2.649918 | 1.331578 | 0.154297 |
| 19 | 1 | 0 | -3.223164 | 0.180062 | 2.333741 |
| 20 | 1 | 0 | -1.483761 | 0.275542 | 2.081047 |
| 21 | 1 | 0 | -2.334289 | -1.252851 | 1.782651 |
| 22 | 1 | 0 | -2.632416 | -1.949957 | -0.855354 |
| 23 | 1 | 0 | -4.590001 | -0.096051 | 0.381943 |
| 24 | 1 | 0 | 4.070896 | -2.805877 | 0.425079 |
| 25 | 1 | 0 | 2.38098 | -2.794559 | 0.982538 |
| 26 | 1 | 0 | 2.751029 | -2.763826 | -0.767955 |

**1F-4-5**

**
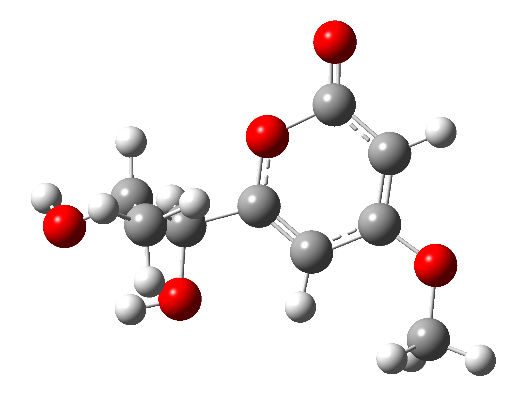
** E = -726.5136785 a.u

| **1F-4-5** | | Standard Orientation  (Ångstroms) | | | |
| --- | --- | --- | --- | --- | --- |
| No. | Atom | Type | X | Y | Z |
| 1 | 8 | 0 | -0.045541 | 1.428407 | -0.372029 |
| 2 | 6 | 0 | -0.207318 | 0.099923 | -0.432987 |
| 3 | 6 | 0 | 0.800806 | -0.77867 | -0.234716 |
| 4 | 6 | 0 | 2.102182 | -0.249424 | 0.050717 |
| 5 | 6 | 0 | 2.296056 | 1.104853 | 0.121261 |
| 6 | 6 | 0 | 1.232519 | 2.038106 | -0.081891 |
| 7 | 8 | 0 | 1.247722 | 3.236837 | -0.046619 |
| 8 | 6 | 0 | -1.625721 | -0.276199 | -0.751369 |
| 9 | 6 | 0 | -2.646561 | 0.232183 | 0.301793 |
| 10 | 6 | 0 | -2.375776 | -0.261854 | 1.711652 |
| 11 | 8 | 0 | -1.72094 | -1.679284 | -0.881986 |
| 12 | 8 | 0 | 3.176074 | -1.035727 | 0.259506 |
| 13 | 8 | 0 | -3.93146 | -0.288008 | -0.084219 |
| 14 | 6 | 0 | 3.036319 | -2.454682 | 0.195539 |
| 15 | 1 | 0 | 0.58454 | -1.832351 | -0.307818 |
| 16 | 1 | 0 | 3.275602 | 1.510155 | 0.335481 |
| 17 | 1 | 0 | -1.879912 | 0.212988 | -1.707438 |
| 18 | 1 | 0 | -2.656277 | 1.326367 | 0.279665 |
| 19 | 1 | 0 | -3.188685 | 0.048063 | 2.369991 |
| 20 | 1 | 0 | -1.440443 | 0.148489 | 2.097527 |
| 21 | 1 | 0 | -2.310158 | -1.351244 | 1.733625 |
| 22 | 1 | 0 | -2.666239 | -1.882045 | -0.8256 |
| 23 | 1 | 0 | -4.279483 | 0.247297 | -0.807001 |
| 24 | 1 | 0 | 4.029128 | -2.850548 | 0.397138 |
| 25 | 1 | 0 | 2.339103 | -2.821321 | 0.9536 |
| 26 | 1 | 0 | 2.710943 | -2.779071 | -0.796399 |
